# Early microbial intervention reshapes phenotypes of newborn *Bos taurus* through metabolic regulations

**DOI:** 10.1093/gigascience/giad118

**Published:** 2024-01-13

**Authors:** Yizhao Shen, Yan Li, Tingting Wu, Quanbin Dong, Qiufeng Deng, Lu Liu, Yanfei Guo, Yufeng Cao, Qiufeng Li, Jing Shi, Huayiyang Zou, Yuwen Jiao, Luoyang Ding, Jianguo Li, Yanxia Gao, Shixian Hu, Yifeng Wang, Lianmin Chen

**Affiliations:** College of Animal Science and Technology, Hebei Agricultural University, Baoding 071000, China; College of Animal Science and Technology, Hebei Agricultural University, Baoding 071000, China; Department of Gastrointestinal Surgery, Changzhou Medical Center, The Affiliated Changzhou No. 2 People's Hospital of Nanjing Medical University, Nanjing Medical University, Changzhou 213164, China; Cardiovascular Research Center, The Affiliated Suzhou Hospital of Nanjing Medical University, Suzhou Municipal Hospital, Gusu School, Nanjing Medical University, Suzhou 215006, China; Department of Cardiology, Nanjing Medical University, The First Affiliated Hospital of Nanjing Medical University, Nanjing 210029, China; Cardiovascular Research Center, The Affiliated Suzhou Hospital of Nanjing Medical University, Suzhou Municipal Hospital, Gusu School, Nanjing Medical University, Suzhou 215006, China; Department of Cardiology, Nanjing Medical University, The First Affiliated Hospital of Nanjing Medical University, Nanjing 210029, China; Department of Cardiology, Nanjing Medical University, The First Affiliated Hospital of Nanjing Medical University, Nanjing 210029, China; Department of Cardiology, Nanjing Medical University, The First Affiliated Hospital of Nanjing Medical University, Nanjing 210029, China; College of Animal Science and Technology, Hebei Agricultural University, Baoding 071000, China; College of Animal Science and Technology, Hebei Agricultural University, Baoding 071000, China; College of Animal Science and Technology, Hebei Agricultural University, Baoding 071000, China; Department of Cardiology, Nanjing Medical University, The First Affiliated Hospital of Nanjing Medical University, Nanjing 210029, China; Department of Cardiology, Nanjing Medical University, The First Affiliated Hospital of Nanjing Medical University, Nanjing 210029, China; Department of Gastrointestinal Surgery, Changzhou Medical Center, The Affiliated Changzhou No. 2 People's Hospital of Nanjing Medical University, Nanjing Medical University, Changzhou 213164, China; College of Animal Science and Technology, Yangzhou University, Yangzhou 225009, China; College of Animal Science and Technology, Hebei Agricultural University, Baoding 071000, China; Hebei Technology Innovation Center of Cattle and Sheep Embryo, Baoding 071000, China; Hebei Research Institute of Dairy Industry Technology, Shijiazhuang 050221, China; College of Animal Science and Technology, Hebei Agricultural University, Baoding 071000, China; Hebei Technology Innovation Center of Cattle and Sheep Embryo, Baoding 071000, China; Hebei Research Institute of Dairy Industry Technology, Shijiazhuang 050221, China; Institute of Precision Medicine, The First Affiliated Hospital, Sun Yat-sen University, Guangzhou 510080, China; Cardiovascular Research Center, The Affiliated Suzhou Hospital of Nanjing Medical University, Suzhou Municipal Hospital, Gusu School, Nanjing Medical University, Suzhou 215006, China; Department of Cardiology, Nanjing Medical University, The First Affiliated Hospital of Nanjing Medical University, Nanjing 210029, China; Department of Gastrointestinal Surgery, Changzhou Medical Center, The Affiliated Changzhou No. 2 People's Hospital of Nanjing Medical University, Nanjing Medical University, Changzhou 213164, China; Department of Cardiology, Nanjing Medical University, The First Affiliated Hospital of Nanjing Medical University, Nanjing 210029, China

**Keywords:** gut microbiome, metagenomics, metabolomics, neonatal calf

## Abstract

**Background:**

The rumen of neonatal calves has limited functionality, and establishing intestinal microbiota may play a crucial role in their health and performance. Thus, we aim to explore the temporal colonization of the gut microbiome and the benefits of early microbial transplantation (MT) in newborn calves.

**Results:**

We followed 36 newborn calves for 2 months and found that the composition and ecological interactions of their gut microbiomes likely reached maturity 1 month after birth. Temporal changes in the gut microbiome of newborn calves are widely associated with changes in their physiological statuses, such as growth and fiber digestion. Importantly, we observed that MT reshapes the gut microbiome of newborns by altering the abundance and interaction of *Bacteroides* species, as well as amino acid pathways, such as arginine biosynthesis. Two-year follow-up of those calves further showed that MT improves their later milk production. Notably, MT improves fiber digestion and antioxidant capacity of newborns while reducing diarrhea. MT also contributes to significant changes in the metabolomic landscape, and with putative causal mediation analysis, we suggest that altered gut microbial composition in newborns may influence physiological status through microbial-derived metabolites.

**Conclusions:**

Our study provides a metagenomic and metabolomic atlas of the temporal development of the gut microbiome in newborn calves. MT can alter the gut microbiome of newborns, leading to improved physiological status and later milk production. The data may help develop strategies to manipulate the gut microbiota during early life, which may be relevant to the health and production of newborn calves.

## Data Description

We followed up on the temporal dynamics of the gut microbiome (104 samples from 3 time points) and plasma metabolome (140 samples from 4 time points) in 36 newborn calves during the first 2 months of life, establishing their relationships with health status and growth/production performance. The metagenomic sequencing data used for the analysis presented in this study are available from the European Nucleotide Archive (ENA) under accession ID PRJEB42631. The metabolic profiles are available from the Metabolomics Workbench under accession ID PR001871.

## Introduction

The colonization and development of the gut microbiota of newborn calves are crucial for the health and performance of the calves later in life [1]. This phenomenon is mainly attributed to the fact that these resident microbes support many functions, including the maturation of the immune system [2, 3], the utilization of nutrients [4], and the prevention of pathogen colonization [5]. Therefore, elucidating the developmental dynamics of gut microbial taxonomy and functionality during early life is important for understanding the relationships between the microbiome and host status and for eventually designing intervention strategies to achieve higher production rates and better health at later stages.

Recent studies have assessed temporal changes in the microbial taxonomic composition of newborn calves during the first week of life [6–8]. For instance, a significant increase in the relative abundance of *Lactobacillus reuteri* was observed during the first week after birth [6]. This fact seems to be very important for calf intestinal health because *L. reuteri* is known to exert bactericidal effects against bacterial pathogens and anti-infective effects against rotaviruses and *Cryptosporidium parvum in vitro* [6]. In addition, comparison of the gut microbial composition between calves (8 weeks after birth) and lactating cows showed that *Bacteroidetes* and *Verrumicrobia* were more abundant in calves, while *Firmicutes, Spirochaetes, Deinococcusthermus, Lentisphaerae, Planctomycetes*, and *Chlorofexi* were more abundant in cows [9]. These observations laid the foundation for targeted mechanistic investigations of the consequences of microbiome colonization for calf health and production.

Nevertheless, several important topics related to the temporal development of the gut microbiome in newborn calves remain unexplored. First, in addition to taxonomy, the functional composition of the gut microbiota can also undergo dynamic changes over time. Microbial functional changes, such as changes in short-chain fatty acid and amino acid metabolic pathways, due to both internal and external disruptions, are implicated in the development of immunity and other systems [10, 11]. However, investigations on the temporal dynamics of gut microbial functionalities in newborn calves are still lacking. Second, the gut microbiome is an ecosystem in which microbes can compete for or exchange nutrients, signaling molecules, or immune evasion mechanisms through complicated ecological interactions that are far from fully understood [12–14]. These interactions can be identified by coabundance network analysis and have been shown to be related to human diseases, including obesity and inflammatory bowel diseases [15]. Investigating temporal changes in microbial interactions during the early life of newborns can enhance our understanding of gut microbial development from an ecological perspective. Third, a favorable microbiome may promote nutrient utilization and immune responses, but it is not clear whether microbial intervention during early life could improve the digestion and health status of newborn calves, as well as their later milk production performance.

To answer the above questions, we conducted the track dairy cattle study (trackDC) in which 36 newborn calves were randomly assigned into 3 groups: a control group, a rumen microbiota transplantation group (RMT), and an autoclaved rumen fluid transplantation group (RFT). All the newborn calves from the 3 groups were followed for 2 months after birth, and intensive phenotype (growth, digestion, and fermentation), blood indicator, plasma metabolome, and stool metagenomic analyses were conducted (Supplementary Fig. S1). In addition, the milk production performance of the calves was recorded during the 2-year follow-up period. We not only investigated the temporal development of the gut microbiome at a metagenomic resolution but also evaluated whether microbiota transplantation (MT) could influence the phenotypes of newborn calves, including their milk production performance later in life.

## Methods

### Animals

The trackDC study is a longitudinal study in northern China that aims to track newborn calves to assess the development of gut microbiota during early life that contributes to cattle health and production. The study was approved by the institutional ethics review board of Hebei Agricultural University (ref. YS19003). In this study, 36 newborn calves were randomly assigned to 3 groups and followed for 2 months after birth. The groups included a control (CON) group, an RMT group, and an RFT group, and intensive data have been collected (Supplementary Fig. S1). The newborn calves were trained to feed milk using a bucket and then transferred to individual calf hutches. Starter was provided *ad libitum* 3 days after birth and once daily in the morning thereafter. Pasteurized whole milk was fed twice daily at 0800 and 1800 h using a bucket, and the calves were weaned 56 days after birth. RMT and RFT were performed by veterinarians, where the ruminal fluid used in RMT and RFT was collected from a healthy cattle (4-year-old, 600 kg, in the dry period) with a permanent rumen cannula 2 hours after the morning feed. Fresh ruminal fluid was mixed with raw milk and fed to the calves in the RMT group immediately after collection. For the RFT group, the ruminal fluid was autoclaved before feeding. A volume of 50 mL, 80 mL, and 110 mL of ruminal fluid was fed from days 7 to 11, days 21 to 25, and days 42 to 46, respectively. Fecal and blood samples were collected at 15, 35, and 56 days after birth. In addition, the milk production performance has also been recorded during the 2-year follow-up.

#### Metagenomic data generation and preprocessing

Fecal samples from newborn calves were collected from rectum by stimulation of the anus and stored in liquid nitrogen after being well mixed by the calf. Aliquots were then made and stored at −80°C until further processing after being transferred to the laboratory. Fecal DNA isolation was performed using the QIAamp Fast DNA Stool Mini Kit (Qiagen, cat. 51604). After DNA extraction, fecal DNA was used for library preparation, and whole-genome shotgun sequencing was performed on the Illumina NovaSeq-6000 platform (RRID:SCR_020150). From the raw metagenomic sequencing data, low-quality reads were discarded by the sequencing facility, and reads belonging to calf and human contaminations were removed by mapping the data to the reference genomes using Bowtie2 (RRID:SCR_016368; v.2.1.0) [16, 17]. After filtering, on average, 36.8 million (SD = 3.6 million) paired reads per sample were obtained for subsequent analysis.

#### Microbial taxonomies

Microbial taxonomic profiles were generated using MetaPhlAn2 (version 2.7.2) [18]. MetaPhlAn2 relies on nearly 1 million unique clade-specific marker genes identified from approximately 17,000 reference genomes, allowing unambiguous taxonomic assignments, accurate estimation of organismal relative abundance, and species-level resolution for bacteria, archaea, eukaryotes, and viruses. Microbial species present in more than 10% of the samples were included for further analyses. This yielded a list of 125 species that accounted for 99% of the original species abundance.

#### Microbial pathways

Microbial pathways were determined using HUMAnN2 (RRID:SCR_016280) [19]. HUMAnN2 reported the abundances of gene families from the UniProt Reference Clusters [20] (UniRef90), which were further mapped to microbial pathways from the MetaCyc metabolic pathway database [21, 22]. In total, we identified 345 pathways that were present in at least 10% of samples, retaining 100% of the original functional composition.

#### Microbial antibiotic resistance genes

The abundance of microbial antibiotic resistance genes in metagenomics was determined using shortBRED (version 0.9.5) [23], with markers generated from the CARD database of bacterial antibiotic resistance genes [24] (01/11/2018 version). In brief, ShortBRED is a platform for identifying a set of protein sequences from a target database (i.e., ResFinder), clustering them into families, building consensus sequences to represent the families, and then reducing these consensus sequences to a set of unique identifying strings (markers). The platform then searches for these markers in metagenomic data and determines the presence and abundance of the protein families of interest. We classified the abundance of 148 antibiotic resistance genes that were present in at least 10% of the samples.

#### Microbial virulence genes

The abundance of microbial virulence genes was detected using shortBRED (version 0.9.5) [23] and markers generated from virulence factors of the pathogenic bacteria database (VFDB, core dataset of DNA sequences, version: November 2018) [25]. Then, we classified the abundance of 55 virulence genes that were present in at least 10% of the samples.

#### Growth

The initial body weight was measured immediately after birth, and the final body weight was measured on day 56 after birth before morning feeding. Body size, including withers height, body length, heart girth, abdominal circumference, and shank circumference, was measured on day 0 and day 56 after birth. The starter offered to and refused by each calf were recorded daily during the experimental period. The starter offered was collected by week, and refusal was collected daily, pooled by calf weekly. Both offered and refused starter samples were oven-dried at 55°C for 48 hours weekly to determine the dry matter (DM) content. The daily starter DM intake was calculated as the difference between daily starter DM offered and starter DM refused.

#### Digestibility and fecal score

Feed digestibility was determined using acid detergent insoluble ash as an internal marker [26]. Briefly, fecal, starter, and milk samples were collected from days 13 to 15, days 33 to 35, and days 54 to 56, and then, the samples from each calf were pooled, dried at 55°C for 48 hours, and then ground through a 1-mm screen for further analyses. The contents of DM (method 930.15) and crude protein (CP; method 996.11) in the starter, milk, and fecal samples were determined according to AOAC International. The contents of neutral detergent fiber (NDF) and acid detergent fiber (ADF) in the starter and feces were measured using heat-stable α-amylase and sodium sulfite as described by Van Soest et al. [27]. The apparent total tract digestibility was estimated as described by Rice et al. [28]. The fecal score was monitored and recorded once daily after morning feed on every calf, using a 4-level scoring system, as described by Larson et al. [29].

#### Blood biomarkers

Plasma samples were used to analyze the concentrations of blood urea nitrogen (BUN), glucose, total cholesterol and triglycerides, and serum samples were used to analyze the concentrations of total protein, albumin, alkaline phosphatase, aspartate aminotransferase (AST), alanine aminotransferase (ALT), total antioxidant capacity, and malonaldehyde. All the blood biomarkers were analyzed using commercial kits from Nanjing Jiancheng Bioengineering Institute. The interassay coefficients of variation were lower than 10%, and the intra-assay coefficients of variation were lower than 12%.

#### Ruminal volatile fatty acids and ammonia

Ruminal fluid was collected at day 56 using an oral stomach tube before morning feeding [30]. Ruminal pH was measured immediately after collection using a pH mater (Starter 300; Ohaus Instruments Co. Ltd.). Two subsamples of 5 mL were transferred into 10-mL screw-lid centrifuge tubes after filtering through 4-layer cheesecloth. One subsample was mixed with 1 mL 25% (wt/vol) HPO_3_ for volatile fatty acid (VFA) analysis, and another subsample was mixed with 1% (wt/vol) H_2_SO_4_ for ammonia analysis. The concentration of ruminal VFAs was measured using gas chromatography (GC-14B, Shimadzu; 30 m × 0.32 mm × 0.25 mm; column temperature, 110°C; injector temperature, 180°C; and detector temperature, 180°C) [31]. The concentration of ruminal ammonia was determined as described by Rhine et al. [32].

#### Untargeted plasma metabolome

Plasma samples resuspended with prechilled 80% methanol. Then the samples were incubated on ice for 5 minutes and centrifuged at 15,000 × *g*, 4°C for 20 minutes. The supernatant was injected into the liquid chromatograph mass spectrometer (LC-MS/MS) system (a ThermoFisher Vanquish UHPLC system coupled with an Orbitrap Q ExactiveTMHF mass spectrometer). The raw data files generated by ultra-high-performance liquid chromatography (UHPLC-MS/MS) were processed using the Compound Discoverer 3.1 (CD3.1; ThermoFisher) to perform peak alignment, peak picking, and quantitation for each metabolite. The normalized data were used to predict the molecular formula based on additive ions, molecular ion peaks, and fragment ions. And then peaks were matched with the mzCloud, mzVault, and MassList database to obtain the accurate qualitative and relative quantitative results. Metabolites were annotated using the KEGG database, HMDB database, and LIPIDMaps database.

#### Microbial diversity

The microbial alpha (Shannon index) and beta (Bray–Curtis dissimilarity) diversities were calculated at the species level by using the R (v3.6.0) package *vegan*.

#### Microbial composition dissimilarity

To compare the differences in overall microbial species composition between and within calves in each group at different time points, dimensionality reduction was carried out by using the t-distributed stochastic neighbor embedding (t-SNE) algorithm with the R package *Rtsne*. Microbiome compositional differences between groups and time points were assessed based on the first and second t-SNE components.

#### Microbial species and pathway coabundance networks

Microbial species and pathway coabundance networks were identified by using the SparCC algorithm [33]. In detail, species composition data from MetaPhlan2 were converted to predicted read counts by multiplying relative abundances by the total sequence counts [15] and then subjected to SparCC. For pathway analysis, the read counts from HUMAnN2 cells were directly used for SparCC. Significant coabundance was controlled at the *P* < 0.01 level using 100 times resampling.

#### Heterogeneity of microbial coabundances

To assess the variability of networks between groups and different time points, we conducted Cochran *Q* tests to assess the heterogeneity of effect sizes and directions for each coabundance (correlation coefficient generated by SparCC). Here, we treated each subgroup as 1 study and conducted Cochran’s *Q* test using the metagen function from the package *meta* (v4.9.5) in R, which calculates the squared difference between individual study effects and the pooled effect using inverse variance weighting [34]. For each coabundance, the *P* values from the Cochran *Q* test were recorded, and coabundances with significant heterogeneity were controlled at the false discovery rate (FDR) 0.05 level determined by Benjamini–Hochberg (BH) correction.

#### Group-specific microbial coabundances

For heterogeneous coabundances (Cochran *Q* test FDR < 0.05), we further assessed whether these relationships showed group specificity, that is, whether the effect size of coabundance (SparCC correlation coefficient) in 1 group was very different from that in the other 2 groups. We adopted interquartile ranges (IQRs) based on the outlier detection method [35]. The IQR was calculated based on the effect size of coabundances in each group, and we assessed whether the smallest or largest effect size fell outside of Q1−0.75 IQR or Q3+0.75 IQR. If only 1 met the condition, we called this coabundance specific and assigned it to the corresponding group.

#### Differential phenotypic, microbial, and metabolic features

The relative abundances of both species and pathway datasets were centered log-ratio transformed, followed by inverse-rank transformation, before subsequent analysis [36]. No transformation was applied to phenotypic data. The ranked-based Kruskal test was then applied to assess (i) whether features within certain groups showed differences between different time points and (ii) whether features within certain time points showed differences between different groups. The FDR was calculated by using the BH method [37].

#### Microbial changes linked to phenotypic and metabolic changes

We calculated microbial and phenotypic changes between days 15 and 35 and between days 15 and 56. We further linked microbial changes to host phenotypic and metabolic changes with Spearman correlation. Associations with *P* < 0.01 were considered significant.

#### Mediation linkage inference

For phenotypic and metabolic associations to the same microbial feature, we first checked whether the human phenotype was associated with the metabolite using Spearman correlation (*P* < 0.01). Next, mediation analysis was carried out using the mediate function from the R package mediation (version 4.5.0) to infer the causal role of the microbiome in contributing to the host phenotype through metabolites.

## Results

### The trackDC study

**Figure 1:**
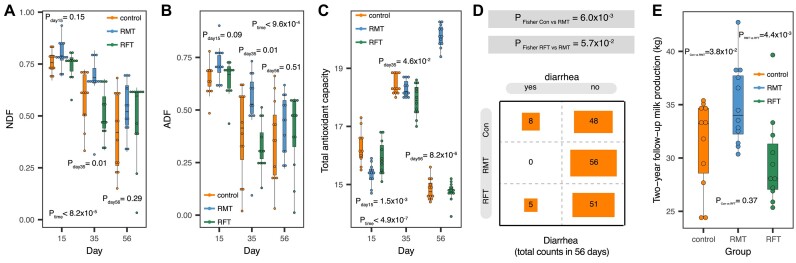
Temporal and microbial transplantation–induced variations of phenotypes in newborn calves. (A) Temporal changes of NDF digestibility. *P* values from Kruskal test are shown accordingly. (B) Temporal changes of ADF digestibility. *P* values from Kruskal test are shown accordingly. (C) Temporal changes of plasma total antioxidant capacity. *P* values from Kruskal test are shown accordingly. (D) Occurrence of diarrhea between groups in the whole experiential period (56 days). *P* values from Fisher exact test are shown accordingly. (E) Differences in the later milk production of those calves during the 2-year follow-up. *P* values from Wilcoxon test are shown accordingly.

To investigate the temporal development of the calf gut microbiome and the potential benefits of early microbial intervention in newborn calves, we collected fecal samples from 36 newborn calves in 3 groups: a control group, an RMT group, and an RFT group, as part of the trackDC. Twelve newborn calves were randomly assigned to each group, and we recorded intensive phenotypes, including growth, digestion, ruminal fermentation, and blood measurements at 1, 15, 35, and 56 days after birth (Supplementary Fig. S1, Supplementary Table S1). We observed that 14 of 17 traits did not exhibit differences between the groups on day 1, indicating a high degree of comparability in the baseline (Supplementary Table S1). We also observed 29 temporal differences and 3 differences between the groups (FDR < 0.05, Supplementary Table S2). For instance, the digestion rates of acid detergent fiber (ADF) and neutral detergent fiber (NDF) showed temporal differences and were significantly higher in the RMT group than in the other groups, indicating that RMT can promote the digestion of fiber by newborn calves (Fig. 1A, B, Supplementary Table S2). Moreover, we also observed potential beneficial effects of RMT, as the blood levels of total antioxidant capacity were the highest in the RMT group compared to the other groups (Fig. 1C, Supplementary Table S2). By comparing the incidence of diarrhea in the 3 groups, we found that the RMT group had significantly fewer cases of diarrhea (Fig. 1D). Notably, the 2-year follow-up showed that the RMT group had significantly higher milk production than the other groups (Fig. 1E). Taken together, these results indicate the beneficial effects of early microbial interventions on the health and growth of newborn calves, as well as their later milk production performance.

### Temporal variations in the gut microbial composition and ecological interaction of newborn calves

**Figure 2:**
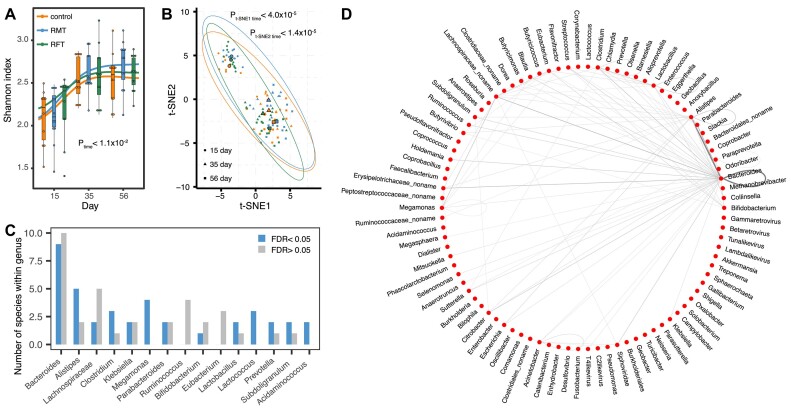
Temporal variations of the gut microbiome in newborn calves. (A) Temporal changes of the Shannon index based on species-level abundance. *P* values from Kruskal test are shown accordingly. (B) Temporal changes of the gut microbial composition on species-level abundance. *P* values from Kruskal test are shown accordingly. (C) Number of species showed temporal differences within different genus. (D) Summary of differential microbial species coabundances between time points at the genus level. Each line represents differential species coabundances between species from either the same or different genera. The width and darkness of the lines represent the relative number of differential coabundances.

To describe the temporal development of the calf gut microbiome, we first evaluated the microbial composition and diversity. A rapid increase in the microbial alpha diversity was observed between 15 and 35 days in all 3 groups (*P*_Kruskal test_ < 1.1 × 10^−2^), but there was no significant difference between 35 and 56 days (*P*_Kruskal test_ > 0.05, Fig. 2A). In addition, dimensionality reduction using the t-SNE algorithm further showed that the microbial composition at day 15 was significantly different from that at days 35 and 56 (*P*_Kruskal test_ < 4.0 × 10^−5^), while no difference was observed between days 35 and 56 (*P*_Kruskal test_ > 0.05, Fig. 2B).

For individual microbial species and pathways, we observed that the abundances of 65 of 125 species (52.0%) and 174 of 345 pathways (50.4%) were significantly different among the 3 time points (FDR < 0.05, Kruskal test, Supplementary Tables S3–S4). Importantly, the temporal development of the gut microbiome was mainly reflected in *Bacteroides* species and the amino acid and nucleotide biosynthesis pathways. In detail, 9 of 65 differential species were from the genus *Bacteroides* (Fig. 2C, Supplementary Table S3), and 50 of 174 pathways were related to amino acid and nucleotide biosynthesis (Supplementary Table S4). In addition, temporal changes in 80 microbial antibiotic resistance genes and 34 virulence genes were observed (FDR < 0.05, Supplementary Table S5). Interestingly, 57 of 80 differential microbial antibiotic resistance genes were from *Escherichia coli* (Supplementary Table S5), a widely recognized pathogenic species. Notably, when comparing the mean abundances of microbial species and pathways between time points, we observed that microbial species abundances at day 35 and day 56 were more similar than those at day 15, while pathway abundances remained relatively stable throughout (Supplementary Fig. S2). We observed that the abundance of 29 species increased with time (*P* < 0.05), including 5 species from *Bacteroides*, 3 species from *Alistipes*, and 3 species from *Lachnospiraceae*. Meanwhile, the abundance of 23 species decreased with time (*P* < 0.05), including 4 species from *Clostridium*, 3 species from *Bacteroides*, and 3 species from *Megamonas* (Supplementary Table S3). Taken together, these results suggested that the gut microbial diversity and composition of newborn calves undergo dynamic changes during the first month of life.

In addition to the microbial composition, we further investigated whether microbial interactions, in terms of microbial species and pathway coabundances, also exhibited differences between different time points in newborn calves. By using the SparCC algorithm [33], we established microbial coabundance relationships in each subgroup separately and identified 2,393 unique species coabundances and 38,964 pathway coabundances (*P*_SparCC_ < 0.01, Supplementary Tables S6–S7). To assess whether microbial coabundance strengths could be different depending on the time after birth, we assessed to what extent the correlation coefficients were variable across subgroups and observed that on average, 42.3% (ranging from 40.5% to 43.3%) of the species coabundances and 2.9% (ranging from 0.2% to 7.0%) of the pathway coabundances showed heterogeneity between different time points (Cochran *Q* test, FDR < 0.05, Supplementary Fig. S3A, Supplementary Tables S6–S7). We next summarized the number of differential coabundances between species from the same genus or from different genera (Fig. 2D). The genus with the most heterogeneous coabundances was *Bacteroides*, and many variable coabundances were observed not only between different *Bacteroides* species but also between *Bacteroides* species and species from other genera such as *Alistipes* (Fig. 2D). A similar observation was found for the pathway coabundances, particularly for the nucleotides and amino acid biosynthesis pathways, which showed variability not only within themselves but also with respect to various pathways related to nucleotide biosynthesis (Supplementary Fig. S3B). These results indicate that the gut microbiome of newborn calves also undergoes dynamic temporal changes in species interactions after birth, while pathway interactions are relatively stable over time.

### Early microbial transplantation reshapes the gut microbiome composition of newborn calves

**Figure 3:**
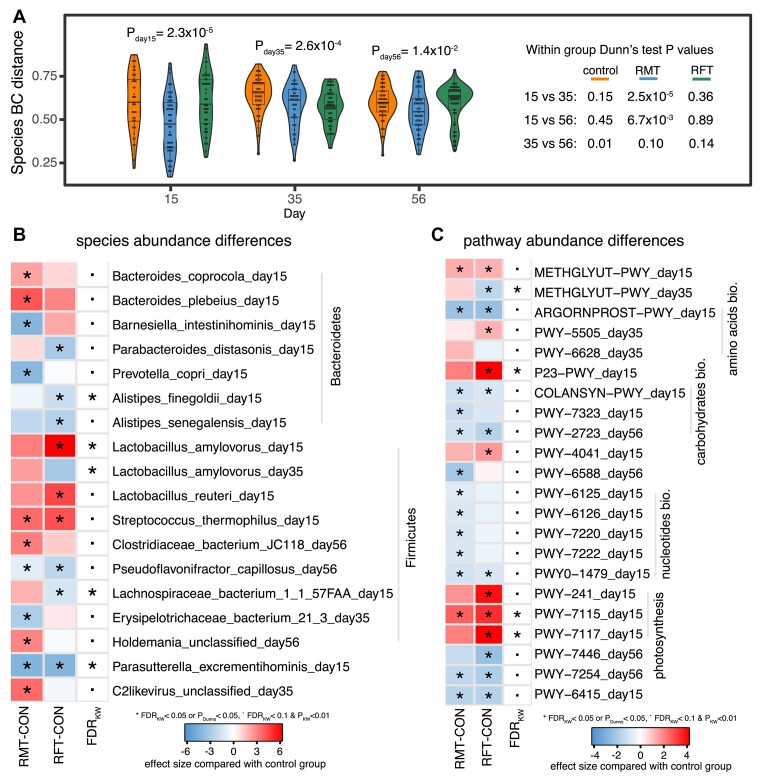
Microbiota transplantation reshapes the gut microbiome composition of newborn calves. (A) Within-group microbial compositional similarity. The Bray–Curtis (BC) distance represents the dissimilarity of microbial species composition between 2 samples. *P* values from Kruskal and Dunn’s tests are shown. (B) Differential species abundance between groups. The darkness of color represents the effect size of microbial abundance when comparing with the control group. *P* and FDR values from Kruskal and Dunn’s tests are shown. (C) Differential pathway abundance between groups. METHGLYUT-PWY, superpathway of methylglyoxal degradation; RGORNPROST-PWY, arginine, ornithine, and proline interconversion; PWY-5505, L-glutamate and L-glutamine biosynthesis; PWY-6628, superpathway of L-phenylalanine biosynthesis; P23-PWY, reductive TCA cycle I; COLANSYN-PWY, colanic acid building block biosynthesis; PWY-7323, superpathway of GDP-mannose–derived O-antigen building block biosynthesis; PWY-2723, trehalose degradation V; PWY-4041, gamma-glutamyl cycle; PWY-6588, pyruvate fermentation to acetone; PWY-6125, superpathway of guanosine nucleotide *de novo* biosynthesis II; PWY-6126, superpathway of adenosine nucleotide *de novo* biosynthesis II; PWY-7220, adenosine deoxyribonucleotide *de novo* biosynthesis II; PWY-7222, guanosine deoxyribonucleotide *de novo* biosynthesis II; PWY0-1479, tRNA processing; PWY-241, C4 photosynthetic carbon assimilation cycle, NADP-ME type; PWY-7115, C4 photosynthetic carbon assimilation cycle, NAD-ME type; PWY-7117, C4 photosynthetic carbon assimilation cycle, PEPCK type; PWY-7446, sulfoquinovose degradation I; PWY-7254, TCA cycle VII (acetate-producers); PWY-6415, L-ascorbate biosynthesis V. The darkness of color represents the effect size of microbial abundance when comparing with the control group. *P* and FDR values from Kruskal and Dunn’s tests are shown.

As the gut microbial composition likely reaches maturity 1 month after birth, the accumulating evidence of the importance of the gut microbiota for overall newborn development indicates the need for early modification of the microbiota. Here, we carried out RMT by the oral infusion of fresh ruminal fluid collected from healthy adult cattle. To overcome the potential bias of metabolites in ruminal fluid, which may also influence the development of gut microbiota, we included a group of calves infused with sterilized ruminal fluid. By calculating the intercalf Bray–Curtis dissimilarity based on the abundances of all the microbial species, we observed that inter-calf dissimilarities within the RMT group were always the lowest among those of all 3 groups at different time points ( *P*_Kruskal test_ < 1.4 × 10^−2^, FDR < 0.05, Fig. 3A). This observation is proof of concept that RMT can reshape the gut microbiome composition of newborn calves, as within-group gut taxonomical compositions appear more identical in the RMT group (Fig. 3A). When comparing individual microbial species and pathways, the relative abundance of 4 species and pathways showed a significant difference at FDR < 0.05 between groups (Kruskal test, Supplementary Tables S3–SS4). We observed 2 species from *Bacteroides* genus, and many nucleotide, amino acid, and carbohydrate biosynthesis pathways (e.g., arginine, ornithine, and proline interconversion and superpathway of GDP-mannose–derived O-antigen building block biosynthesis pathways) were differentially abundant in the RMT group (FDR < 0.1, Fig.   3B, C). Notably, differential microbial abundances between groups were mainly observed at day 15, and most of them were driven by RMT (Fig. 3B, C), suggesting that RMT had pronounced effects in reshaping the gut microbial composition of newborn calves in the early days of life.

### Microbial interactions show specificity in the transplantation group

For microbial coabundances, we have also checked to what extent the correlation coefficients were variable between groups, and the numbers were 40.4% (ranging from 37.1% to 43.5%) and 0.3% (ranging from 0.1% to 0.6%) for species and pathway coabundances, respectively (Cochran *Q* test, FDR < 0.05, Supplementary Fig. S4A, Supplementary Tables S6–S7). These results indicate that microbial interventions can alter the interactions that may potentially contribute to the development of host phenotypes. Interestingly, heterogeneous species coabundances characterized by comparing different groups were widely distributed in many genera (Supplementary Fig. S4B), and these coabundances were far more complex than those characterized by comparing temporal differences between different time points (Supplementary Fig. S3A). However, this was not the case in the pathway coabundances (Supplementary Fig. S4C).

**Figure 4:**
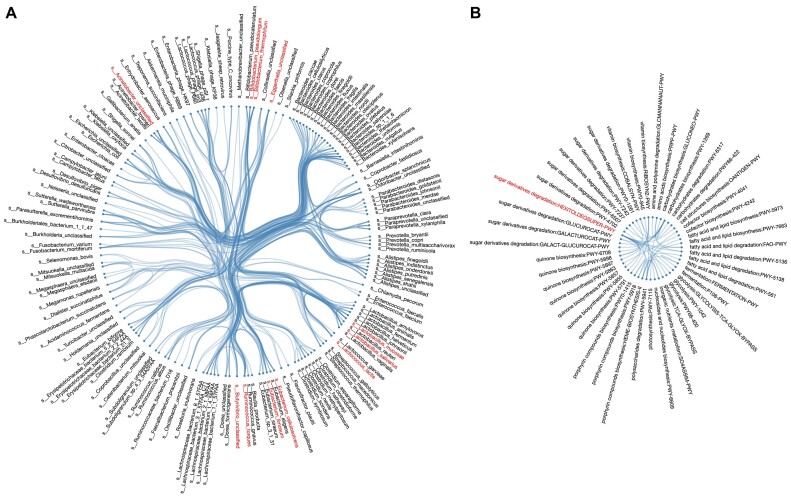
Microbiota transplantation–specific species and pathway coabundances. (A) In total, 248 microbial intervention-specific species coabundances. Each dot indicates 1 species while each line represents a microbial intervention–specific correlation between 2 species. Species in red are hub nodes with hub score larger than 0.5. (B) Forty-two microbial intervention–specific pathway coabundances. Each dot indicates 1 pathway while each line represents a microbial intervention–specific correlation between 2 pathways. Pathway in red is the hub node with hub score larger than 0.5.

As many of the species and pathway coabundances showed heterogeneity between groups at different time points, we further analyzed whether those heterogeneous coabundance relationships were driven by a particular group, that is, whether the coabundance strength in 1 group was very different from those in the other 2 groups at each time point. In general, we identified 633 and 101 unique group-specific species and pathway coabundances, respectively (Supplementary Tables S6–S7). Notably, 248 of 633 (39.3%) species coabundances and 42 of 101 (41.6%) pathway coabundances showed specificity for the RMT group (Fig. 4A, B, Supplementary Tables S6–S7), indicating that RMT can also alter the gut microbiome of newborn calves at the ecological level.

For those group-specific coabundances, we further evaluated potential hub species and pathways by calculating their hub score (Supplementary Tables S8–S9). Interestingly, we observed a substantial amount of RMT-specific species coabundances related to *Bifidobacterium* (Fig. 4A), a common genus that colonized calves early in life [38, 39]. In the meantime, those species also had high hub scores (Supplementary Table S8). For instance, *Bifidobacterium thermophilum* was one of the species with the most RMT-specific coabundances (10 in total, Supplementary Table S6) and with a high hub score of 0.98 (Supplementary Table S8). *B. thermophilum* constitutes 80% of the infant microbiota and less than 10% of the human adult microbiota, and the presence of *Bifidobacterium* in the gut is often associated with health-promoting effects [40]. In addition, RMT-specific pathway coabundances mainly involved sugar derivative degradation and quinone biosynthesis pathways (Fig. 4B), but only the hexitol degradation pathway had a high hub score of 1.00 (Supplementary Table S9). The sugar derivative degradation pathway-related coabundances showed specificity for the RMT group, which was reasonable as we observed that the digestion rates of NDF and ADF were relatively higher in the RMT group than in the other 2 groups (Fig. 1A, B).

### Microbial changes associated with plasma metabolites in newborn calves

To understand the potential mechanisms by which the gut microbiota could influence host physiology, we thought that metabolites are an important class of molecules that are involved in the host–microbe interaction. By profiling plasma levels of 736 metabolites at different time points using untargeted LC-MS (Supplementary Table S10), we observed that the plasma metabolome shifts with time in newborn calves (Supplementary Fig. S5), and 50.3% of individual metabolites (370 in total) showed significant differences between groups in at least 1 time point with FDR <0 .05 (Kruskal test, Supplementary Table S11).

**Figure 5:**
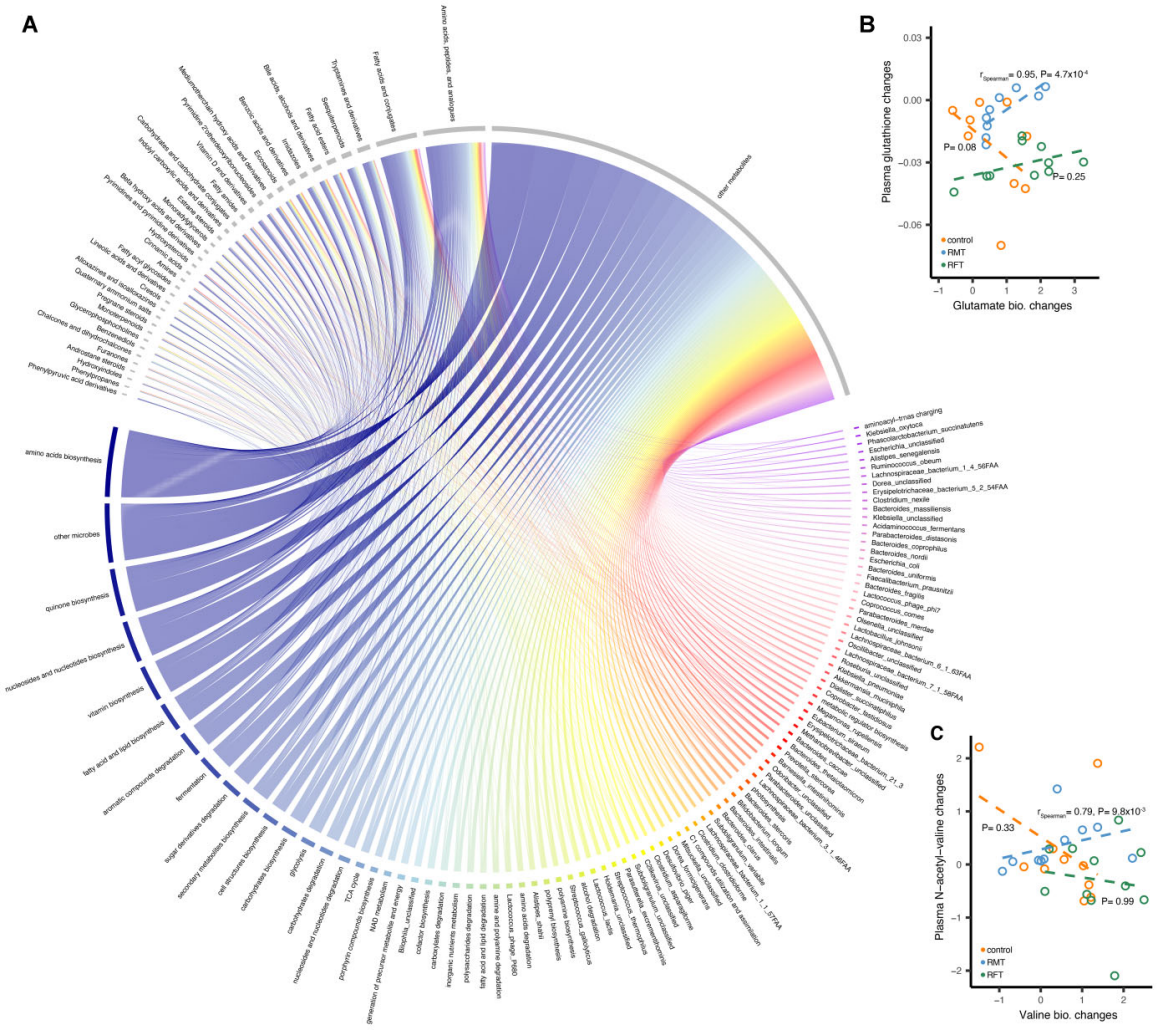
Microbial compositional changes associated with plasma metabolic changes. (A) Overview of 5,221 microbial intervention–specific microbe–metabolite associations between days 15 and 35, as well as days 15 and 56. The associated microbial factors are colored gray, and the associated metabolites are colored by other colors. (B) Positive association between plasma glutathione and microbial glutamate biosynthesis pathway changes in the microbial intervention group between days 15 and 35. (C) Positive association between plasma N-acetyl-valine and microbial valine biosynthesis pathway changes in the microbial intervention group between days 20 and 35. Spearman correlation is applied to assess the associations between microbial and phenotypic changes.

We then checked metabolic changes specifically in relation to changes in microbial composition between days 15 and 56 and between days 15 and 35. In total, we observed 17,602 associations between microbial and metabolite changes, and 5,221 of them were RMT specific (Spearman correlation, r_absolute_ > 0.7, *P* < 0.01, Fig. 5A, Supplementary Tables S12–S13). Notably, various metabolites associated with the microbiome are already known to be related to the gut microbiome, including animal essential amino acids, bile acids, organic acids, and others [3]. For instance, increased abundance of the microbial L-glutamate biosynthesis pathway (PWY-5505) was associated with increased levels of plasma glutathione (r_RMT_ = 0.95, *P*_RMT_ = 4.7 × 10^−4^, Fig. 5B), a tripeptide compound consisting of glutamate attached via its side chain to the N-terminus of cysteinyl glycine. Glutathione is an antioxidant that prevents oxidative damage through the reduction of methemoglobin and peroxides [41]. We also observed that increased abundance of the microbial L-valine biosynthesis pathway (VALSYN-PWY) was associated with the increased levels of plasma N-acetyl-valine (r_RMT_ = 0.79, *P*_RMT_ = 9.8 × 10^−3^, Fig. 5C). Valine is a branched-chain amino acid that cannot be biosynthesized by animals and plays important roles in insulin resistance and hematopoietic stem cell self-renewal [42, 43]. Taken together, those results suggested that RMT-induced temporal changes of the gut microbiome in newborn calves may contribute to their metabolic changes.

### Microbial changes associated with the phenotypes of newborn calves

To examine the role of gut microbiota colonization in newborn calves, we explored the associations between microbial compositional changes and host phenotypes. To this end, we first calculated the microbial differences between days 15 and 56 and between days 15 and 35. Next, microbial changes were associated with their corresponding phenotypic changes, including changes in growth, digestion, fermentation, and blood indicators (Supplementary Table S1). In general, we observed 381 significant associations that involved 79 species, 185 pathways, and 16 phenotypes (Spearman correlation, r_absolute_ > 0.7, *P* < 0.01, Supplementary Tables S14–S15). Interestingly, we observed that 66.7% and 50.0% differential species and pathways between groups also had at least 1 significant association to temporal changes in phenotypes (Supplementary Tables S14–S15). Such species mainly included *Lactobacillus* (*Lactobacillus amylovorus* and *Lactobacillus reuteri*) and *Alistipes* species (*Alistipes finegoldii* and *Alistipes senegalensis*), while pathways mainly included guanosine and adenosine nucleotides, as well as L-ascorbate biosynthesis. Most of the microbial associations to host phenotypes were related to changes in serum cholesterol, total protein, globulin, and albumin levels, suggesting the importance of gut microbial development to newborn calf lipid metabolism and immunity. For instance, changes in the abundance of the microbial saturated fatty acid elongation pathway were associated with changes in serum cholesterol levels (r_Spearman_= −0.85, *P* = 1.6 × 10^−3^, Supplementary Table S14). In addition, we observed that changes in serum globulin were associated with 4 prokaryotic ubiquinol biosynthesis pathways (r_Spearman_ > 0.79, *P* < 6.1 × 10^−3^, Supplementary Table S15). Ubiquinols can promote coenzyme activities to enhance lipophilic antioxidants and thus simulate host immunity [44].

**Figure 6:**
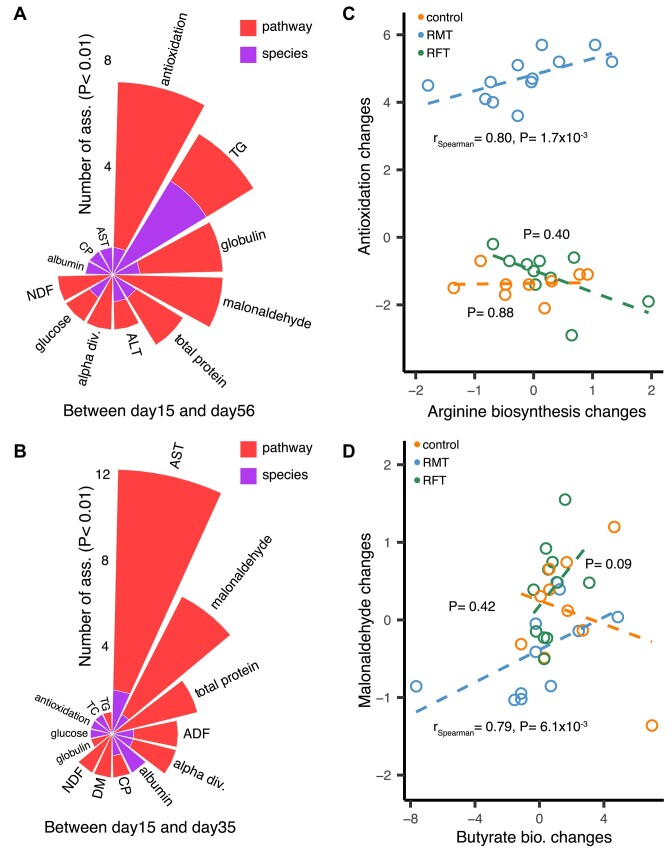
Microbial compositional changes associated with phenotypic changes in newborn calves. (A) Microbial changes between days 15 and 56 associated with the corresponding phenotypic changes. (B) Microbial changes between days 15 and 35 associated with the corresponding phenotypic changes. (C) Positive association between antioxidation capacity and microbial arginine biosynthesis pathway changes in the microbial intervention between days 15 and 56. (D) Positive association between blood malonaldehyde and microbial butyrate biosynthesis pathway changes in the microbial intervention group between days 15 and 35.

Notably, 77 of 381 associations were RMT specific (i.e., only showed significance in the RMT group or change in the opposite direction when compared with other groups, r_absolute_ > 0.7, *P* < 0.01, Supplementary Tables S14–S15), and 54 associations were related to microbial pathways (Fig. 6A, B). Among those associations, many were related to serum AST, malonaldehyde, total antioxidation capacity, and digestion of fiber (ADF and NDF). This suggests that RMT may potentially influence liver health, energy homeostasis, antioxidation, and digestion, as reflected by changes the traits listed above (Supplementary Tables S3–S4). For example, we observed that an increased abundance of microbial arginine biosynthesis was also associated with serum antioxidation capacity (r_Spearman_= 0.80, *P* = 1.7 × 10^−3^, Fig. 6C). Arginine can effectively reduce oxidative stress through the arginine/nitric oxide pathways [45]. We also observed that an increased abundance of microbial butyrate biosynthesis was associated with serum malonaldehyde levels (r_Spearman_ = 0.79, *P* = 6.1 × 10^−3^, Fig. 6D). Supplementation with butyrate induced a marked shift in superoxide dismutase and catalase activities, along with a decrease in malonaldehyde levels, thereby attenuating oxidative stress [46]. These results suggest that RMT may potentially promote phenotypes of newborn calves by modulating microbial functionalities.

### RMT alters phenotypic changes of newborn calves through metabolites

**Figure 7:**
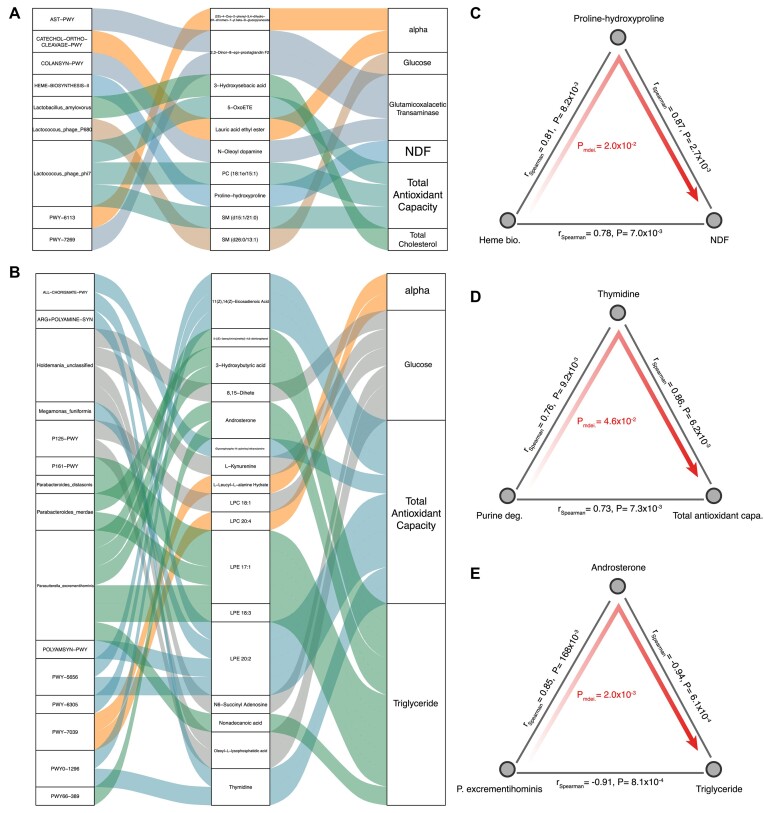
Mediation linkages among the gut microbial, metabolic, and phenotypic changes. (A) Sankey plot showing the 11 significant mediation effects of plasma metabolites between days 15 and 35. (B) Sankey plot showing the 29 significant mediation effects of plasma metabolites between days 15 and 56. The left panel shows the microbial factors, the middle panel shows the plasma metabolites, and the right panel shows the phenotypes. The curved lines across panels indicate the mediation effects, while the colors correspond to different phenotypes. (C) Proline–hydroxyproline mediates the effect of microbial heme biosynthesis pathway on NDF digestion. (D) Thymidine mediates the effect of microbial purine degradation pathway on total antioxidant capacity. (E) Androsterone mediates the effect of *Parasutterella excrementihominis* on triglyceride.

Since microbial changes can be linked to changes in both phenotypes and metabolites in newborn calves, we hypothesized that microbial impacts on host phenotypes may mediate by metabolites. To evaluate whether metabolites can mediate the microbial impact on host phenotypes, we applied mediation analysis focusing on 46 microbial features that are associated with both phenotypic and metabolic changes, which revealed 40 mediation linkages (*P*_mediation_ < 0.05, Fig. 7A, B, Supplementary Tables S16–S17). Those linkages were related to microbial impact on various phenotypes, including fiber digestion, antioxidant capacity, and lipid and glucose metabolism via a variable category of metabolites (Fig. 7A, B). For example, we showed that the microbial heme biosynthesis pathway may contribute to an increase in NDF digestion by increasing plasma proline–hydroxyproline levels (*P*_mediation_ = 0.02, Fig. 7C). Proline-rich proteins are known as negative regulators that participate in modulating fiber [47]. For antioxidant capacity, we showed that the microbial purine degradation pathway may contribute to an increase in antioxidant capacity by increasing plasma thymidine levels (*P*_mediation_ = 0.02, Fig. 7D). Thymidine catabolism may promote NADPH oxidase–derived reactive oxygen species to induce oxidative stress [48]. We also found that a Proteobacteria species *Parasutterella excrementihominis* may contribute to the decrease in plasma triglyceride by increasing plasma androsterone levels (*P*_mediation_ = 0.02, Fig. 7E). Androsterone is an effective lipid-lowing agent [49]. In summary, these results suggest that microbial changes induced by RMT may reshape phenotypes of newborn calves through modulating host metabolism.

## Discussion

A diverse microbial population colonizes the mammalian gastrointestinal tract during/after birth, and increasing evidence now suggests that this complex microbiome plays a crucial role in the development of the mucosal immune system and influences newborn health [1, 50]. Recent studies have tracked the temporal changes of the gastrointestinal microbiota in newborn calves during the first several weeks after birth at a 16S rRNA resolution, which is limited on taxonomic composition [6–8, 51]. However, the key to understanding the importance of microbial development to the host is to investigate whether within-calf microbial differences can be associated with changes in various phenotypes. We therefore systematically characterized the microbial changes at both the taxonomic and functional levels by using fecal metagenomic sequencing data from 36 newborn calves in the trackDC study.

Previous investigations on the temporal development (within 2 months) of the microbial composition of newborn calves with 16S rRNA sequencing have revealed a list of microbial genera that exhibit significant temporal differences, including *Enterococcus, Lactobacillus, Escherichia, Bifidobacterium, Clostridium*, and so on [6–8, 51]. Our in-depth metagenomic sequencing of the gut microbiome extends this observation at the species resolution, which enhances the current understanding. In addition, characterization of the temporal changes in microbial pathway abundances further showed that microbial functionalities, such as amino acid, organic acid, and carbohydrate metabolism, undergo dynamic changes in newborn calves.

This result was mainly attributed to the fact that microbe colonization of the gastrointestinal tract and their functionalities contribute to changing the ruminant digestive system from a monogastric system to a fully functional foregut rumen fermenter system, with an ability to digest fibrous feed, postweaning.

The period from birth to weaning is important for rumen microbial colonization and adaptation. Once the development and maturation of the rumen and the microbiome are complete, it is difficult to permanently manipulate or change the rumen ecosystem due to microbial adaptation and resilience to external mediators [52]. In early life, a favorable microbiome can be implanted via dietary or management interventions and has potentially a long-lasting effect [1, 53, 54].

Here, we showed that the gut microbiome of newborn calves can be altered by RMT at both taxonomic and functional levels, as indicated by species and pathway abundances, respectively. For instance, we observed a higher abundance of the beneficial species *Parabacteroides distasonis* in the RMT group, and this species can alleviate metabolic dysfunctions by generating succinate and secondary bile acids, which activate the intestinal gluconeogenesis pathway and farnesoid X receptor, in the gut [55]. In addition, we showed that microbial interactions in terms of coabundances showed heterogeneity between groups and characterized many RMT-specific species and pathway coabundances. The diverse microbial communities in the gut make up a complicated ecosystem in which microbes can exchange or compete for nutrients, signaling molecules, or immune evasion mechanisms through ecological interactions that are far from fully understood [12, 13]. Our analyses show that microbial alterations by RMT may not be driven solely by differences in abundance level; it may also reflect shifts in microbial interactions that are mirrored in coabundance analyses. Particularly when applied to metagenomics sequence data, pathway-based coabundance networks provide further insights into the functional alterations caused by RMT, as many RMT-specific pathway coabundances have been identified.

Characterization of the temporal changes in the gut microbiome is crucial for understanding the role of the gut microbiome in phenotypic development of newborn calves. By linking microbial changes to phenotypic changes in newborn calves in different groups, we observed thousands of associations between the microbiome, phenotypes, and metabolism, including digestion, lipid metabolism, and immunity. Interestingly, many of those associations were only present in the RMT group. For example, RMT may increase arginine production, which further enhances the antioxidation capacity. Another example is increased butyrate production in response to the increased amount of malonaldehyde in the RMT group. With mediation analysis by linking microbial, phenotypic, and metabolic changes, our analysis suggests that microbial changes induced by RMT may reshape phenotypes of newborn calves through modulating host metabolism. Thus, our longitudinal analysis of microbial association with calf phenotypes and blood indicators revealed functional insights and putative causality of the role of the gut microbiome in newborn calf health status. These observations are of great importance for guiding further studies to develop strategies that may be used to manipulate the early microbiome to improve production and health during the time when newborn calves are most susceptible to enteric disease.

We acknowledge several limitations in the present study. First, the trackDC study sampled fecal and blood samples from 36 newborn calves during the first 2 months after birth, making it the first metagenomics- and metabolomics-based longitudinal study with the largest sample size to date. However, despite our random assignment of calves to various groups, the absence of baseline fecal microbiome profiles could potentially introduce bias to the observed differences. And the conducted analysis, such as mediation analysis, may underpower. Therefore, replicating these findings in independent studies with larger sample sizes like human cohort studies [56] could significantly enhance the robustness of our observations and emphasize their biological significance. Second, we employed various intervention volumes with the intention of mitigating potential bias arising from the natural growth of the gastrointestinal tract in newborn calves over time. However, it is important to note that this approach could introduce new biases, as we did not directly measure the actual size of their gastrointestinal tracts. Third, the reported results are association based, which means that the underlying causalities and mechanisms of action remain unexplored. Functional studies are thus essential to further reveal the underlying mechanisms of the reported associations. Finally, we primarily focused on the gut microbiome of newborn calves. However, given the fact that the temporal development of their rumen microbiome is also important but not easily sampled, further studies linking the gut and rumen microbiomes in newborn calves may provide a more systematic understanding of the importance of the microbiome in newborn calves.

## Availability of Source Code and Requirements

- Project name: The trackDC study
- Project homepage: https://github.com/MicrobiomeCardioMetaLab/trackDC.abundance_project
- Operating system(s): Platform independent
- Programming language: R
- Other requirements: None
- Any restrictions to use by nonacademics: license GPL-3
- RRID: SCR_020150; SCR_016368; SCR_016280

## Supplementary Material

giad118_GIGA-D-23-00086_Original_Submission

giad118_GIGA-D-23-00086_Revision_1

giad118_GIGA-D-23-00086_Revision_2

giad118_Response_to_Reviewer_Comments_Original_Submission

giad118_Response_to_Reviewer_Comments_Revision_1

giad118_Reviewer_1_Report_Original_Submission Huanzi Zhong, Ph.D. -- 5/29/2023 Reviewed

giad118_Reviewer_1_Report_Revision_1 Huanzi Zhong, Ph.D. -- 9/14/2023 Reviewed

giad118_Reviewer_2_Report_Original_Submission Zhipeng Li -- 6/1/2023 Reviewed

giad118_Reviewer_2_Report_Revision_1 Zhipeng Li -- 9/5/2023 Reviewed

giad118_Supplemental_Tables

## Data Availability

The metagenomic sequencing data used for the analysis presented in this study are available from the European Nucleotide Archive (ENA) under accession ID PRJEB42631. The metabolic profiles are available from the Metabolomics Workbench under accession ID PR001871. All supporting data and materials are available in the *GigaScience* database, GigaDB [57].

## Additional Files

**Supplementary Fig. S1**. Study design and overview of the data that had been collected during the first 2 months.

**Supplementary Fig. S2**. Comparison of microbial feature abundances between groups at different time points. Spearman correlation is applied to assess the associations of microbial abundances between different time points.

**Supplementary Fig. S3**. Temporal differences in microbial coabundance networks. (A) Summary of microbial species and pathway coabundances in each group at different time points. The number of significant coabundances has been listed and the heterogeneity of coabundances is assessed by Cochran *Q* test. (B) Summary of differential microbial pathway coabundances between time points at metabolic category level. Each line represents differential pathway coabundances between pathways from either the same or different metabolic categories. The width and darkness of the lines represent the relative number of differential coabundances.

**Supplementary Fig. S4**. Differential microbial coabundance networks between groups. (A) Summary of microbial species and pathway coabundances in each group at different time points. The number of significant coabundances has been listed and the heterogeneity of coabundances is assessed by Cochran *Q* test. (B) Summary of differential microbial species coabundances between groups at the genus level. Each line represents differential species coabundances between species from either the same or different genera. The width and darkness of the lines represent the relative number of differential coabundances. (C) Summary of differential microbial pathway coabundances between groups. Each line represents differential pathway coabundances between pathways from either the same or different metabolic categories. The width and darkness of the lines represent the relative number of differential coabundances.

**Supplementary Fig. S5**. The composition of plasma metabolites shows temporal and group differences. *P* values from Kruskal test are shown accordingly.

**Supplementary Table S1**. Summary of phenotypes collected in this study.

**Supplementary Table S2**. Phenotypic differences between groups and time points.

**Supplementary Table S3**. Differential microbial species abundances between groups and time points.

**Supplementary Table S4**. Differential microbial pathway abundances between groups and time points.

**Supplementary Table S5**. Differential microbial antibiotic resistance and virulence gene abundances between groups and time points.

**Supplementary Table S6**. Microbial species coabundances.

**Supplementary Table S7**. Microbial pathway coabundances.

**Supplementary Table S8**. Hub scores of species in coabundance networks.

**Supplementary Table S9**. Hub scores of pathways in coabundance networks.

**Supplementary Table S10**. Summary of plasma metabolome.

**Supplementary Table S11**. Differential plasma metabolites between groups.

**Supplementary Table S12**. Microbial changes between days 15 and 35 associated with metabolic changes.

**Supplementary Table S13**. Microbial changes between days 15 and 56 associated with metabolic changes.

**Supplementary Table S14**. Microbial changes between days 15 and 35 associated with phenotypic changes.

**Supplementary Table S15**. Microbial changes between days 15 and 56 associated with phenotypic changes.

**Supplementary Table S16**. Metabolites mediate microbial impacts on phenotypic changes between days 15 and 35.

**Supplementary Table S17**. Metabolites mediate microbial impacts on phenotypic changes between days 15 and 56.

## Abbreviations

ADF: acid detergent fiber; ALT: alanine aminotransferase; AST: aspartate aminotransferase; BUN: blood urea nitrogen; CON: control group; CP: crude protein; DM: dry matter; FDR: false discovery rate; MT: microbiota transplantation; NDF: neutral detergent fiber; RFT: autoclaved rumen fluid transplantation group; RMT: rumen microbiota transplantation group; trackDC: the track dairy cattle study; VAF: volatile fatty acid.
