# Supplementary material for: Early microbial intervention reshapes phenotypes of newborn Bos taurus through metabolic regulations
Source: Gigascience. 2024 Jan 13;13:giad118. doi: 10.1093/gigascience/giad118 (PMC10787367; doi:10.1093/gigascience/giad118)
Supplement: giad118_GIGA-D-23-00086_Revision_1 [file giad118_giga-d-23-00086_revision_1.pdf]

GigaScience

Early microbial intervention reshapes phenotypes of newborn Bos taurus through metabolic regulations

--Manuscript Draft--

**Manuscript Number:**	GIGA-D-23-00086R1
**Full Title:**	Early microbial intervention reshapes phenotypes of newborn Bos taurus through metabolic regulations
**Article Type:**	Research
**Funding Information:**	
**Abstract:**	Background<br><br>The rumen of neonatal calves has limited functionality and establishing intestinal microbiota may play a crucial role in their health and performance. We aim to explore the temporal colonization of the gut microbiome and the benefits of early microbial intervention in newborn calves. We evaluated the impact of microbiota transplantation (MT) on metabolism and later milk production performance.<br><br>Results<br><br>We followed 36 newborn calves for two months and found that the composition and ecological interactions of their gut microbiomes likely reached maturity one month after birth. Temporal changes in the gut microbiome of newborn calves are widely associated with changes in their physiological statuses, such as growth and fiber digestion. Importantly, we observed that MT reshapes the gut microbiome of newborns by altering the abundance and interaction of Bacteroides species, as well as amino acid pathways, such as arginine biosynthesis. Two-year follow-up of those calves further showed that MT improves their later milk production. Notably, MT improves fiber digestion, antioxidant capacity of newborns while reducing diarrhea. MT also contributes to significant changes in the metabolomic landscape, and with putative causal mediation analysis, we suggest that altered gut microbial composition in newborns may influence physiological status through microbial-derived metabolites.<br><br>Conclusions<br><br>Our study provides metagenomic and metabolomic atlas of the temporal development of the gut microbiome in newborn calves. MT can alter the gut microbiome of newborns, leading to improved physiological status and later milk production. The data may help develop strategies to manipulate the gut microbiota during early life, which may be relevant to the health and production of newborn calves.
**Corresponding Author:**	Lianmin Chen, Ph.D.<br>Nanjing Medical University<br>Nanjing, Jiangsu CHINA
**Corresponding Author Secondary Information:**	
**Corresponding Author's Institution:**	Nanjing Medical University
**Corresponding Author's Secondary Institution:**	
**First Author:**	Yizhao Shen
**First Author Secondary Information:**	
**Order of Authors:**	Yizhao Shen
	Yan Li
	Tingting Wu
	quanbin dong

Powered by Editorial Manager® and ProduXion Manager® from Aries Systems Corporation

	Qiufeng Deng
	Lu Liu
	Yanfei Guo
	Yufeng Cao
	Qiufeng Li
	Jing Shi
	Huayiyang Zou
	Yuwen Jiao
	Luoyang Ding
	Jianguo Li
	Yanxia Gao
	Shixian Hu
	Yifeng Wang
	Lianmin Chen
**Order of Authors Secondary Information:**	
**Response to Reviewers:**	detailed responses have been uploaded.
**Additional Information:**	
**Question**	**Response**
Are you submitting this manuscript to a special series or article collection?	No
**Experimental design and statistics**<br><br>Full details of the experimental design and statistical methods used should be given in the Methods section, as detailed in our Minimum Standards Reporting Checklist. Information essential to interpreting the data presented should be made available in the figure legends.<br><br>Have you included all the information requested in your manuscript?	Yes
**Resources**<br><br>A description of all resources used, including antibodies, cell lines, animals and software tools, with enough information to allow them to be uniquely identified, should be included in the Methods section. Authors are strongly encouraged to cite Research Resource	Yes

Identifiers (RRIDs) for antibodies, model organisms and tools, where possible.<br><br>Have you included the information requested as detailed in our Minimum Standards Reporting Checklist?	
**Availability of data and materials**<br><br>All datasets and code on which the conclusions of the paper rely must be either included in your submission or deposited in publicly available repositories (where available and ethically appropriate), referencing such data using a unique identifier in the references and in the “Availability of Data and Materials” section of your manuscript.<br><br>Have you have met the above requirement as detailed in our Minimum Standards Reporting Checklist?	Yes

Reply to reviewers

Click here to access/download;Manuscript;reviewer_comments.doc

Reply to comments raised by reviewers:

Reviewer1:

In this manuscript, the authors applied shotgun metagenomics and metabolomics to investigate the temporal development of gut microbiota (GM) in neonatal calves and the effects of rumen microbiota transplantation (RMT) on the calves' GM, metabolism, and later milk production performance. The manuscript is well-written. The study is carefully designed, with an extensive collection of omics data from fecal samples, blood samples, ruminal fluid, and the calves themselves. Comprehensive analyses linking host characteristics, fecal microbial features, and blood metabolites are conducted. The descriptions of the metagenomic methods and statistical approaches are clear and understandable. I have the following suggestions to improve the manuscript.

Reply: We would like to thank the reviewer for those kind words, and now we have addressed the comments accordingly.

Methods:

1） Line 102-Line 118. Two different transplantation methods (RMT and RFT) were applied in this study. The RMT group was treated with fresh ruminal fluid and the RFT group was treated with autoclaved ruminal fluid. Additionally, the intervention volume and feeding period varied a lot during the whole experiment, (e.g., a volume of 50 mL, 80 mL, and 110 mL of ruminal fluid was fed from day 7 to day 11, day 21 to day 25, and day 42 to day 46, respectively). I suggest the author should revise Figure S1 to include more details, specifically highlighting the differences between the two transplantation methods, the intervention periods, and the volume of transplantation. This addition, together with the sampling and measurements, would enhance the clarity of the experimental pipeline for readers.

Reply: Thank you for this suggestion, we have now revised the figure (**Figure S1**) by adding details regarding the differences between groups (ruminal fluid VS autoclaved ruminal fluid), intervention period (day 7 to day 11, day 21 to day 25, and day 42 to day 46) and the volumes (a volume of 50 ml, 80 ml and 110 ml, respectively) that have been used. We used increased volumes with the idea to potentially omit the bias of the increased size of gastrointestinal tract in newborn calves, but we realized that this could also raise new bias since we didn't really measure the size of their gastrointestinal tract. Thus, we further acknowledged this limitation in the discussion "*Secondly, we employed various intervention volumes with the intention of mitigating potential bias arising from the natural growth of the gastrointestinal tract in newborn calves over time. However, it's important to note that this approach could introduce new biases, as we did not directly measure the actual size of their gastrointestinal tracts.*".

Figure S1. Study design and overview of the data that has been collected during the first two months.

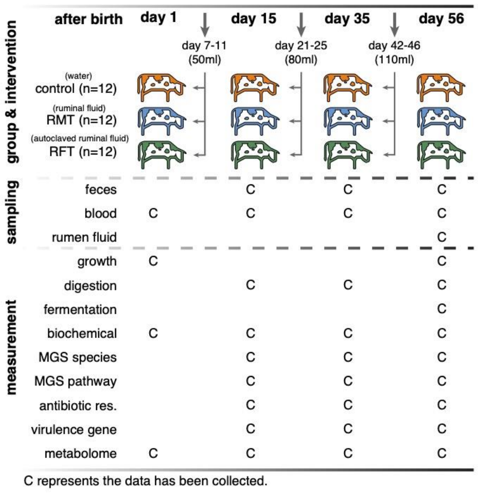

2）Line 112-Line 128. Most previous metagenomic studies have investigated the rumen microbiota and revealed a highly complex microbial community within this specific environment. For this study, the fecal samples of calves yielded an average of 36.8 million (sd= 3.6 million) paired reads per sample. It is important to note that this sequencing depth is significantly lower than what is typically required for rumen samples in ruminant studies. which is much lower than the that required by the rumen samples of ruminants. Therefore, it is unclear whether the authors evaluated the sequencing depth saturation specifically for the fecal samples and whether they consider the obtained depth to be sufficient for their research.

Reply: We agree with the reviewer that our sequencing depth is lower than ruminal samples, for instance an average of 42 million paired reads were obtained from rumen sample in a recent rumen microbial genome assembly study (Tong et al., Nature Communications, 2022). As our read depth of fecal samples rang from 29 to 46 million paired reads, we checked if the increase of read depth positively correlated with number of species detected in each sample and observed non-significant correlation (r=0.17, P=0.09, **Rebuttal Figure 1**). This suggests that our sequencing depth might be sufficient in profiling microbial species abundance in fecal samples.

Rebuttal Figure 1. Correlation between fecal sample sequencing depth and number of detectable species

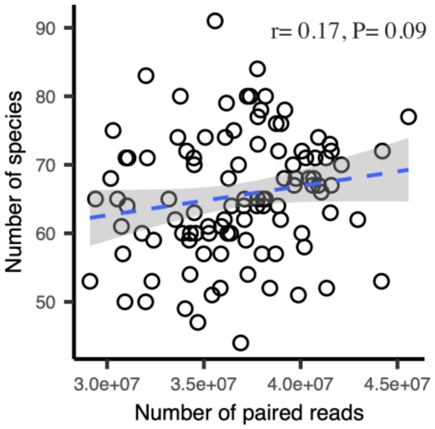

Result:

First, one of the major concerns of the design is the lack of baseline samples before intervention. As shown in the Fig. S1, the first sampling point of the feces was day 15 while the first intervention period was from day 7- day 11. None of baseline microbial data could be acquired to show whether the three groups have overall similar or different fecal microbial community. The second concern could be the lack of rumen microbiota of the fresh ruminal fluid from the healthy donor. Without the data, we are not able to answer how the specific donor affected the receptors' gut microbiota, thus we could hardly genialize the observations/conclusions to larger animal populations. The authors should discuss both as the possible limitations in the manuscript.

Reply: Regarding the microbiome profiles of donor, indeed we have the data and based on the PCoA analysis (**Rebuttal Figure 2**), the microbial composition in rumen fluid (4 samples in red collected during the study) is much different from fecal samples. This is as expected since they are from different locations of the gastrointestinal tract. Thus, we didn't focus too much on it.

We agree with the reviewer that we didn't collect baseline samples to check their fecal microbiome profiles before randomization, which may induce potential bias. Now we have acknowledged this limitation in the discussion "*However, despite our random assignment of calves to various groups, the absence of baseline fecal microbiome profiles could potentially introduce bias to the observed differences. Therefore, replicating these findings in independent studies with larger sample sizes could significantly enhance the robustness of our observations and emphasize their biological significance.*".

Rebuttal Figure 2. The microbial compositional differences between rumen fluid samples from donor and fecal samples from newborn calves.

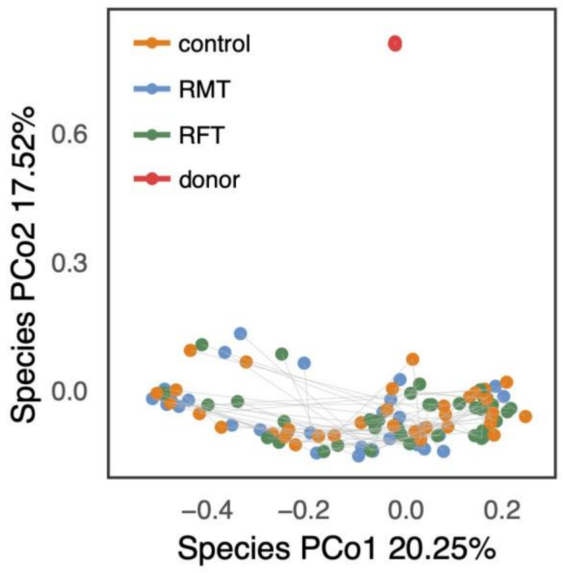

1）Line 257-273. The authors should add some descriptions to show that there were no baseline differences in host characterizes measured at day 1. Line267-269, the results showed that the plasma total antioxidant capacity increased from day 15 to day 35 while dramatically decreased from day 35 to day 56, even reached a level lower than day 15. Why? Could the authors provide any explanations on this temporal changes? Comparisons between different timepoints (day 15, 35 and 56) within each group would be helpful to improve the conclusions for this part (Early microbial interventions using RMT could provide beneficial effects on the health and growth of newborn calves, as well as their later milk production performance). PS, in FigS6, they showed few baseline differences in plasma metabolites between the three groups. I would suggest authors to put some baseline results in the first section.

Reply: By following reviewer's suggestions, we checked whether there was significant difference between groups at day 1 and observed that 14 out of 17 traits showed no difference (**Table S1**), indicating that the baseline indeed very comparable between groups. We have now added those results in the main text "*We observed that 14 out of 17 traits did not exhibit differences between the groups on day 1, indicating a high degree of comparability in the baseline (Table S1).*" Besides, this was also the case as the overall plasma metabolome composition was comparable between groups.

For the total antioxidant capacity, we observed that in the RMT group it increased with time, but there was significant decrease from day 35 to day 56 in control & RFT group (**Rebuttal Figure 3**). As antioxidant capacity is a major reason for diarrhea prevention in calves (*Aydin et al, Microbial Pathogenesis, 2022*). A potential explanation is that we observed more incidence of diarrhea in those two groups (**Figure 1D**), especially at the later stage (from day 35 to day 56).

To further strength our conclusions, we have provided comparison results between different timepoints within each group in the left panel of **Table S2**.

Rebuttal Figure 3. Total antioxidant capacity differences between different day within each group.

phenotype	mean_day15	mean_day35	mean_day56	P.kru	Q.kru
TotalAntioxidantCapacity_rft	15.9181818	17.8916667	14.7583333	3.12E-07	0.0006498
TotalAntioxidantCapacity_rmt	15.3666667	18.3	20.1166667	2.63E-07	0.0006498
TotalAntioxidantCapacity_control	16.29	18.425	14.8333333	4.89E-07	0.0006782

2）Line 274-312. The authors dedicated a lot of effort to elucidate the temporal variations in the gut microbial composition of calves from day 15 to day 56. Overall, their findings are reliable. Across all three intervention groups, they observed significant differences in the gut microbiota between day 15 and either day 35 or day 56, while the gut microbial structure between day 35 and day 56 showed no significant differences (or much smaller differences). However, all of these results are presented in supplementary figures or tables, which makes it inconvenient for readers to access comprehensive information. I would suggest that the authors consolidate the relevant results from the supplementary figures/tables as a main figure (e.g, Fig S2, Fig S3-specise-level based results, Fig S4B and related Stable3-5) to clearly describe the directions of specific temporal changes, e.g, the RA changes of Bacteroides members and the amino acid and nucleotide biosynthesis pathways (increase or decrease), instead of give the number of microbial features differed between timepoints, and the changes of networks for Bacteroides members.

Reply: Thanks for this suggestion, indeed in the previous version we aimed to more focused on group differences instead of temporal differences. Now we have made a main figure (**Figure 2**) for temporal differences by combing Figure S2, Table S3 and Figure S4B.

Figure 2. Temporal variations of the gut microbiome in newborn calves. A. Temporal changes of the Shannon index based on species level abundance. P values from Kruskal test are shown accordingly. **B.** Temporal changes of the gut microbial composition on species level abundance. P values from Kruskal test are shown accordingly. **C.** Number of species showed temporal differences in different genus. **D.** Summary of differential microbial species co-abundances between time points. Each line represents differential species co-abundances between species from either the same or different genera. The width and darkness of the lines represent the relative number of differential co-abundances.

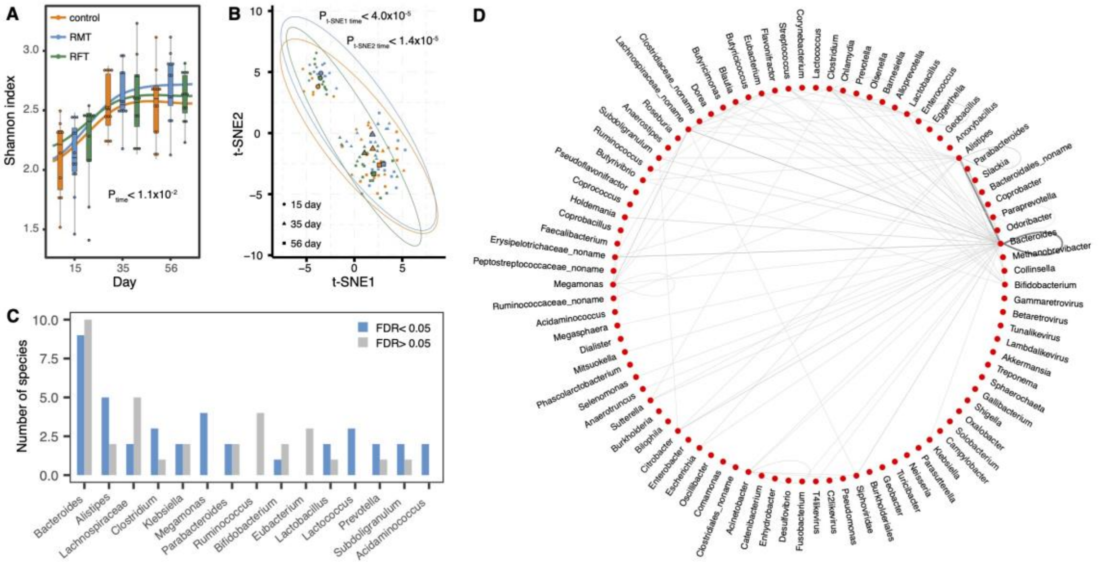

3）Line 290-292. Data from Fig S3 and Fig S4 both suggested that unlike species community, the microbial functional pathways between three timepoints were highly similar (rho>0.9 and ~3% heterogeneity). SO, I suggest that the authors modify their results "Notably, when comparing the mean abundances of microbial species and pathways between time points, we observed that microbial abundances at Day 35 and Day 56 were more similar than those at Day 15 (Figure S3). " Because in Fig SC, the association results at the pathway level did not support the text "pathway abundances at Day 35 and Day 56 were more similar than those at Day 15". You should cite it together with Fig S4A (Line 299-304) to indicate that the overall pathway RA matrix and pathway interactions were relatively stable over time.

Reply: We agree with the reviewer and have revised the sentence "*Notably, when comparing the mean abundances of microbial species and pathways between time points, we observed*

that microbial species abundances at Day 35 and Day 56 were more similar than those at Day 15, while pathway abundances remained relatively stable throughout (Figure S2)."

4）Line 313-331. Fig.2. All conclusions were based on between-group comparisons. However, from this figure, we could hardly get the conclusion that "differential microbial abundances between groups were mainly observed at Day 15, and most of them were driven by RMT". If we look at the data, we did see that in the RMT group, the species BC distance increased from day 15 to day 35 and then decreased from day 35 to day 56. But that patterns seems did not exist in other groups. Could the authors please provide between-timepoint comparisons (BC distance, species changes in the RMT group) within each intervention group together in this figure (instead of putting it in the Stables) to complete their findings? Also, a Dunn's post-hoc test would be needed after the KW test (Fig. 2A-C). For the Fig. 2B-C, the authors utilize the transformed RA as the color key for the heatmap, making it difficult for readers to discern the differences (enrichment directions) among the three intervention groups. I would suggest that they consider using effect size (compared to the control group) as a more suitable measure for the figure. This approach would enhance the clarity of the heatmap and facilitate a clearer understanding of the differences between the control and intervention groups.

Reply: We have now added within group comparisons of BC distance between timepoints (Dunn's test, **Figure 3A**) and observed that indeed there were no difference between day 35 and 56 in both RMT and RFT, but in the RMT group the difference of microbial species composition between timepoints (15 vs 35 and 15 vs 56) were larger than other two groups. We have also revised the heatmaps by using effect size and Dunn's post-hoc test P values have been added (**Figure 3B-C**).

Figure 3. Microbiota transplantation reshapes the gut microbiome composition of newborn calves. A. Within group microbial compositional similarity. The Bray-Curtis (BC) distance represents the dissimilarity of microbial species composition between two samples. P values from Kruskal and Dunn's tests are shown. **B.** Differential species abundance between groups. The darkness of color represents the effect size of microbial abundance when comparing with the control group. P and FDR values from Kruskal and Dunn's tests are shown. **C.** Differential pathway abundance between groups. The darkness of color represents the effect size of microbial abundance when comparing with the control group. P and FDR values from Kruskal and Dunn's tests are shown.

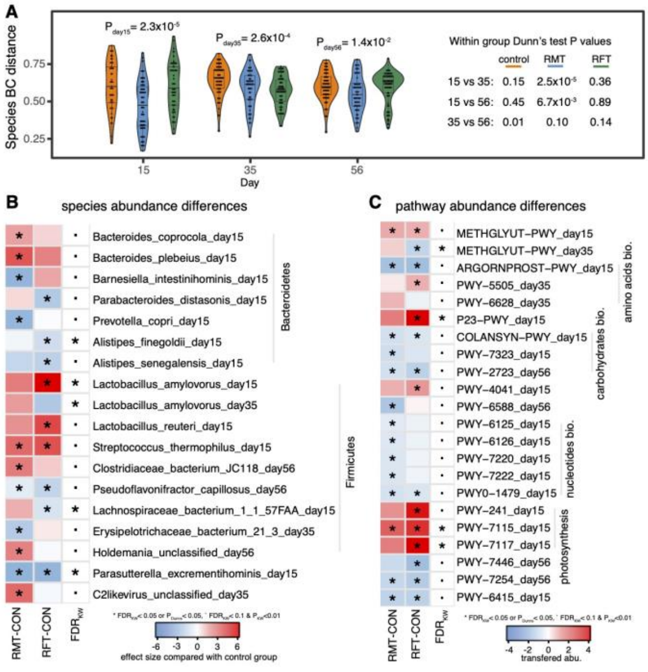

5）Line 332-359. The authors described the differences in species or pathway co-abundance networks between groups, primarily focusing on the number of unique connections within

the RMT group. Could they conduct further analysis to identify potential hub nodes (hub species/pathways) under different conditions? Additionally, highlighting the hub nodes in the figures would greatly enhance the clarity and aid readers in understanding the results more effectively. This additional information would provide valuable insights into the central species or pathways that play critical roles in the observed differences.

Reply: Thanks for this suggestion, now we have utilized the group specific species and pathway co-abundances to calculate the hub score of each node, and those new results have been added in **Table S8** and **Table S9**. We have also highlighted the 11 species (hub score > 0.5) and one pathway (hub score > 0.5) hub node in the RMT group (**Figure 4**). For the main text, we have added *"For those group specific co-abundances, we further evaluated potential hub species and pathways by calculating their hub score (Table S8–9). Interestingly, we observed a substantial amount of RMT-specific species co-abundances related to Bifidobacterium (Figure 4A), a common genus that colonized calves early in life [38, 39]. In the meanwhile, those species also have high hub scores (Table S8). For instance, Bifidobacterium thermophilum was one of the species with the most RMT-specific co-abundances (10 in total, Table S6), and with a high hub score of 0.98 (Table S8). B. thermophilum constitutes 80% of the infant microbiota and less than 10% of the human adult microbiota, and the presence of Bifidobacterium in the gut is often associated with health-promoting effects [40]. In addition, RMT-specific pathway co-abundances mainly involved sugar derivative degradation and quinone biosynthesis pathways (Figure 4B), but only hexitol degradation pathway had a high hub score of 1.00 (Table S9). The sugar derivative degradation pathway-related co-abundances showed specificity for the RMT group, which was reasonable as we observed that the digestion rates of NDF and ADF were relatively higher in the RMT group than in the other two groups (Figure 1A-B).*"

Figure 4. Microbiota transplantation specific species and pathway co-abundances. A. 248 microbial intervention specific species co-abundances. Each dot indicates one species while each line represents a microbial intervention specific correlation between two species. Species in red are hub nodes with hub score larger than 0.5. **B.** 42 microbial intervention specific pathway co-abundances. Each dot indicates one pathway while each line represents a microbial intervention specific correlation between two pathways. Pathway in red is the hub node with hub score larger than 0.5.

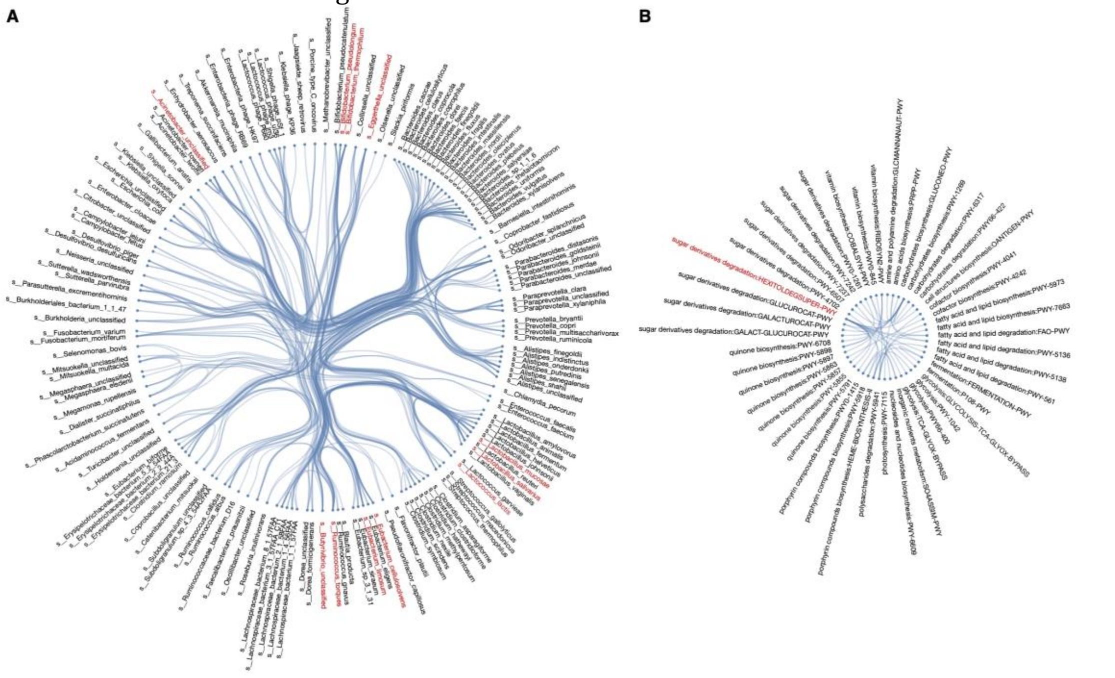

6) Line 360-399. The main issue is the interpretation of association results. As the authors also discussed in their manuscript, they might overinterpret their association results: "we observed that an increased abundance of Megamonas funiformis may potentially promote

the antioxidation capacity of newborn calves" "An increased abundance of M. funiformis may contribute to arginine biosynthesis (rSpearman= 0.65, P=Figure 4E), indicating that RMT may increase M. funiformis abundance to potentially promote arginine production, which further enhances the antioxidation capacity." We further…., indicating that increased abundance of R. gnavus may potentially promote butyrate production in response to the increased amount of malonaldehyde in the RMT group. These results suggest that RMT may potentially promote phenotypes of newborn calves by modulating microbial functionalities." From a logical perspective, the functions attributed to a particular species or taxon should be supported by evidence from potential coding genes and pathways within their genomes (considered as first-level evidence). Therefore, it is recommended that the authors avoid solely attributing changes in metabolites to their associations with the gut microbiota, specifically relying on correlations between alterations in the relative abundances of microbial species and blood metabolites. Furthermore, it is worth noting that the increased RAs of M. funiformis and R. gnavus have previously been reported to be enriched in humans with obesity or inflammatory bowel disease (IBD). Considering this, it raises questions as to why the authors concluded that these microbial changes might be beneficial in the RMT group. Although MP2/MP3 has the potential to annotate the microbial community in human samples well, it remains uncertain to what extent it can accurately represent the complexity of the gastrointestinal microbial community in ruminants. This is also why most current published studies prefer to employ an assembly-based strategy to investigate this type of microbial community. It is advisable for the authors to exercise caution and refrain the results for this particular section. (e.g., the changes in several specific blood metabolites should reflect more directly to a beneficial outcome of the RMT group. Think you could spend more words on these points.)

Reply: We totally agree with the reviewer that interprets such results with only significant associations is not rigor. Besides, we realized that M. funiformis and R. gnavus stories might be also not that suitable. By taking those concerns into account, we have now revised the examples to only keep microbial arginine biosynthesis pathway associated with antioxidation changes and microbial butyrate biosynthesis pathway associated with antioxidation changes (**Figure 6C-D**).

Figure 6. Microbial compositional changes associated with phenotypic changes in newborn calves. A. Microbial changes between day 15 and day 56 associated with the corresponding phenotypic changes. **B.** Microbial changes between day 15 and day 35 associated with the corresponding phenotypic changes. **C.** Positive association between antioxidation capacity and microbial arginine biosynthesis pathway changes in the microbial intervention between day 15 and day 56. **D.** Positive association between blood malonaldehyde and microbial butyrate biosynthesis pathway changes in the microbial intervention group between day 15 and day 35.

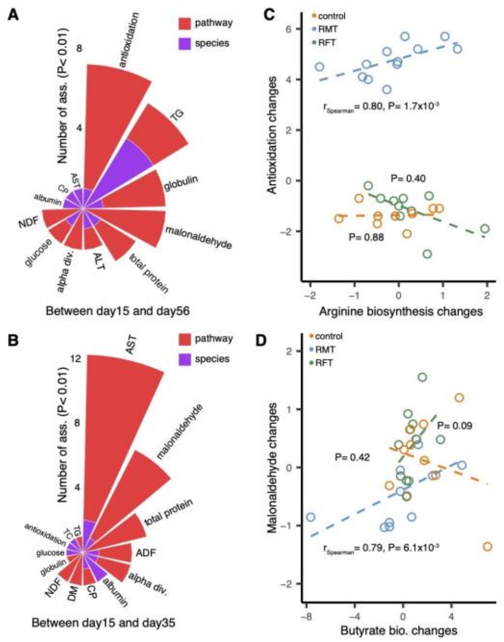

And the main text has also been revised "*Notably, 77 out of 381 associations were RMT specific (i.e., only showed significance in the RMT group or change in the opposite direction when compared with other groups, rabsolute> 0.7, P< 0.01, Table S14–15), and 54 associations were related to microbial pathways (Figure 6A-B). Among those associations, many were related to serum aspartate transaminase (AST), malonaldehyde, total antioxidation capacity, and digestion of fiber (ADF and NDF). This suggests that RMT may potentially influence liver health, energy homeostasis, antioxidation and digestion, as reflected by changes the traits listed above (Table S3–4). For example, we observed that an increased abundance of microbial arginine biosynthesis was also associated with serum antioxidation capacity (rSpearman= 0.80, P= 1.7×10-3, Figure 6C). Arginine can effectively reduce oxidative stress through the arginine/nitric oxide pathways [45]. We also observed that an increased abundance of microbial butyrate biosynthesis was associated with serum malonaldehyde levels (rSpearman= 0.79, P= 6.1×10-3, Figure 6D). Supplementation with butyrate induced a marked shift in superoxide dismutase and catalase activities, along with a decrease in malonaldehyde levels, thereby attenuating oxidative stress [46]. These results suggest that RMT may potentially promote phenotypes of newborn calves by modulating microbial functionalities.*".

Regarding the taxonomic profiling tools, we agree with the reviewer that MP2/MP3 frequently used in human studies, but indeed the background taxonomic database was based on all genomes from NCBI (*Truong et al, Nature Methods, 2015*). In this case, MP2/MP3 shouldn't be human specific. But the unclassified microbes in ruminants also attractive and we have done such a work (*Dong et al, bioRxiv, 2023.03. 30.535011*).

7) Line 360-399. Microbial changes associated with the phenotypes of newborn calves
Line 400-421. Microbial changes associated with plasma metabolites in newborn calves
I would suggest combining the two sections and adjusting their order as follows: First, for the relationship between changes in gut microbiota and metabolites. Then, explore the relationships between changes in gut microbiota and phenotypes. Currently, in the section titled "Microbial changes associated with the phenotypes of newborn calves," the authors have already integrated some information regarding the changes in metabolites and gut microbiota. By reorganizing the sections in this manner, the flow of information will be improved, providing a clearer understanding of the relationships between three parties.

Reply: Thanks, now we have adjusted the order of those two parts and please check it in the main text.

8) Line 422-439. The main concern for this section is the relatively small samples size for mediation analysis. It is crucial to conduct a power analysis to determine the minimum sample size required for mediation analysis.

Reply: We agree with the reviewer that power analysis for the mediation analysis is important. Now we have checked the minimum sample size required for mediation analysis in this study by using *wp.mediation()* function from the WebPower R package. With a minimal power of 0.7, based on the current data (effect sizes of x~m and m~y), we need on average 25 samples, thus our reported mediations results may under power. However, we still think those results are valuable despite the small sample size. Thus, we further acknowledged this limitation in the discussion *"the trackDC study sampled fecal and blood samples from 36 newborn calves during the first two months after birth, making it the first metagenomics and metabolomics-based longitudinal study with the largest sample size to date. However, despite our random assignment of calves to various groups, the absence of baseline fecal microbiome profiles could potentially introduce bias to the observed differences. And the conducted analysis, such as mediation analysis may under power. Therefore, replicating these findings in independent studies with larger sample sizes could significantly enhance the robustness of our observations and emphasize their biological significance."*.

9）Discussion: Please revise it according to the above suggestions.
Reply: We have now revised the discussion accordingly, please check the text marked in red.

Reviewer2:

In this study, Shen et al. investigated the gut microbiota colonization, and evaluated the effect of rumen microbiota and metabolites transplantation. Although many studies have examined the gut microbiota development of different ruminant, however, the RMT is interesting. The present version limited to the large part of description in difference or association. More analysis is need to answer why RMT can reshape the gut microbiota and host metabolism based on metagenome and metabolome.
Reply: We would like to thank the reviewer for those kind suggestions. Now we have added some new analysis e g., microbial co-abundance network hub analysis & summary analysis in differential microbial traits between group. Together with the mediation analysis to show how microbial shifts may potentially influence host phenotypic changes though regulating plasma metabolite, we think now the revised the manuscript may give the field more information and results. But we have also acknowledged that the current study is mainly association based and in-depth mechanistic exploration may further strength the conclusions. Thus, we further changed the title to "*Early microbial intervention reshapes phenotypes of newborn Bos taurus through metabolic regulations*" with the idea to downplay a little bit.

L59. What is modification of nutrition? Please explain.
Reply: Sorry for the confusion, we have now revised this sentence in the main text "*This phenomenon is mainly attributed to the fact that these resident microbes support many functions, including the maturation of the immune system [2], the utilization of nutrients [3], and the prevention of pathogen colonization [4].*"

L77. However, investigations of the temporal dynamics of microbial functionalities of newborn calves are still lacking. Malmuthuge et al. (2019) has examined the development of rumen microbiome through metagenomes.
Reply: We agree with the reviewer and have revised this sentence by focus on the gut microbiome of newborn calves "*However, investigations on the temporal dynamics of gut microbial functionalities in newborn calves are still lacking.*"

L85. it is not clear at what time the gut microbiome reaches maturity in calves. The authors conducted the rumen microbiota transplantation but examined the gut microbiota maturity. Make no sense. Moreover, the gut microbiota development has reported in jejunum, cecum, and colon, such as Zhuang et al. (2020) and Fan et al. (2021).
Reply: We agree with the reviewer that it is confusion. We have involved three groups, one control group and two intervention groups (RMT & RFT) and indeed the control group can

be used to study the maturity of the gut microbiome in newborn calves. We have now downplayed the sentence "*Third, studying the maturity of the gut microbiome in newborn calves is important because it may indicate a suitable time frame for reshaping the gut microbiome through targeted intervention before maturation.*".

L115. Why the authors choose 50 mL, 80 mL, and 110 mL?
Reply: Sorry for the confusion. We used increased volumes with the idea to potentially omit the bias of the increased size of gastrointestinal tract in newborn calves, but we realized that this could also raise new bias since we didn't really measure the size of their gastrointestinal tract. Thus, we further acknowledged this limitation in the discussion "*Secondly, we employed various intervention volumes with the intention of mitigating potential bias arising from the natural growth of the gastrointestinal tract in newborn calves over time. However, it's important to note that this approach could introduce new biases, as we did not directly measure the actual size of their gastrointestinal tracts.*".

L138. Please define customized database.
Reply: We have now give more explanations about this in the main text "*Microbial pathways were determined using HUMAnN2 [17]. HUMAnN2 reported the abundances of gene families from the UniProt Reference Clusters [18] (UniRef90), which were further mapped to microbial pathways from the MetaCyc metabolic pathway database [19, 20].*"

L245. Since we observed that microbial differences between Day 35 and Day 56 were minor. This is a result before conducing statistics analysis. So rewrite the sentence.
Reply: Agree, we have now revised it to "*We calculated microbial and phenotypic changes between Day 15 and Day 35 and between Days 15 and 56. We further linked microbial changes to host phenotypic and metabolic changes with Spearman correlation.*"

L251. Why the author examined the human phenotype?
Reply: Sorry, it's a typo, now we have changed it to "*Next, mediation analysis was carried out using the mediate function from the R package mediation (version 4.5.0) to infer the causal role of the microbiome in contributing to the host phenotype through metabolites.*"

L277. Change x.
Reply: We have now changed it to "×" throughout the main text.

L320. Although the inter-calf dissimilarities within the RMT group were always the lowest among those of all three groups at different time points, however, the t-SNE results showed that the gut microbiota in the two treatment groups is not significant different, and only 4 species and pathways showed a significant difference. These limited results can not provide the solid support to reshape the gut microbiome composition.
Reply: We agree with the reviewer that indeed not that much differences have been characterized at strict FDR<0.05 level, but indeed we observed hundreds of microbial difference at P<0.01. This may due to the fact that the sample size of our study is still limited, even though our study is the largest sample size with a wide range of phenotypes and paired metagenomics and metabolomics data. Thus, we further acknowledge this in the discussion "*Therefore, replicating these findings in independent studies with larger sample sizes could significantly enhance the robustness of our observations and emphasize their biological significance.*"

L326-328. Differential microbial species were enriched in the Bacteroidetes and Firmicutes families, while differential pathways were mostly enriched in nucleotide, amino acid and carbohydrate biosynthesis pathways. Bacteroidetes and Firmicutes were families? Most of microbial species can be classified as the phyla Bacteroidetes and Firmicutes. Moreover, the nucleotide, amino acid and carbohydrate biosynthesis pathways were too general.
Reply: Sorry for the confusion, to avoid such general conclusions, now we have revised the sentences by pointing to specific species and pathways "*We observed two species from*

Bacteroides genus and many nucleotide, amino acid and carbohydrate biosynthesis pathways (e.g., arginine, ornithine and proline interconversion and superpathway of GDP-mannose-derived O-antigen building blocks biosynthesis pathways) were differential abundant in the RMT group (FDR < 0.1, Figure 3B-C). Notably, differential microbial abundances between groups were mainly observed at Day 15, and most of them were driven by RMT (Figure 3B-C), suggesting that RMT had pronounced effects in reshaping the gut microbial composition of newborn calves in the early days of life.".

L342. many of the species and pathway co-abundances showed heterogeneity between groups at different time points. This may be Ture, as stochastic colonization in early life has long-lasting impacts on the development of animal microbiomes (Furman, 2020).

Reply: We agree with the reviewer that microbial interactions may shift with time during early stage and microbial intervention may potentially reshapes it.

L366. 79 species, 185 pathways were identified in the associations. Is there any difference for these species and pathways among the groups? Is there any trend or distribution in these species and pathways? More analysis is need. The present results contributed to limited novel insights for understanding the relationship between gut microbiota and host phenotype.

Reply: Thanks for those suggestions, to make the results more detailed instead of just report numbers, we have checked 1) whether microbial traits have significant associations to phenotypes also differential abundant between groups. 2) Is there any trend or distribution in these species and pathways. The new information now have been added in the main text "*Interestingly, we observed that 66.7% and 50.0% differential species and pathways between groups also had at least one significant association to temporal changes in phenotypes (Table S14–15). Such species mainly including Lactobacillus (Lactobacillus amylovorus and Lactobacillus reuteri) and Alistipes species (Alistipes finegoldii and Alistipes senegalensis), while pathways mainly including guanosine and adenosine nucleotides, as well as L-ascorbate biosynthesis. Most of the microbial associations to host phenotypes were related to changes in serum cholesterol, total protein, globulin and albumin levels, suggesting the importance of gut microbial development to newborn calf lipid metabolism and immunity. For instance, changes in the abundance of the microbial saturated fatty acid elongation pathway were associated with changes in serum cholesterol levels (rSpearman= -0.85, P= 1.6×10-3, Table S14). In addition, we observed that changes in serum globulin were associated with 4 prokaryotic ubiquinol biosynthesis pathways (rSpearman> 0.79, P< 6.1×10-3, Table S15). Ubiquinols can promote coenzyme activities to enhance lipophilic antioxidants and thus simulate host immunity [44].*"

L405. The results showed that the metabolites at day 35 was different from that at day 56. However, this result is different from the observed results in gut microbiota. Why?
The font size in figures 3, 5 and 6 is too small.

Reply: The composition of the gut microbiome in newborn calves is comparable between day 36 and 56, but the plasma metabolome seems different. We thought a potential reason is that the host metabolome is only partially dependent on the gut microbiome, other factors including environment, genetics and diet are also important (*Chen et al, Nature Medicine, 2022*).

For the small font size in figures, now we have revised them according to the journal guidance.

Early microbial intervention reshapes phenotypes of newborn *Bos taurus* through metabolic regulations

Yizhao Shen[1†], Yan Li[1†], Tingting Wu[2,3,4†], Quanbin Dong[3,4†], Qiufeng Deng[4], Lu Liu[4], Yanfei Guo[1], Yufeng Cao[1], Qiufeng Li[1], Jing Shi[4], Huayiyang Zou[4], Yuwen Jiao[2], Luoyang Ding[5], Jianguo Li[1,6,7], Yanxia Gao[1,6,7]*, Shixian Hu[8]*, Yifeng Wang[3,4]* & Lianmin Chen[2,4]*

[1] College of Animal Science and Technology, Hebei Agricultural University, Baoding, China

[2] Changzhou Medical Center, The Affiliated Changzhou No.2 People's Hospital of Nanjing Medical University, Nanjing Medical University, Changzhou, China

[3] Cardiovascular Research Center, The Affiliated Suzhou Hospital of Nanjing Medical University, Suzhou Municipal Hospital, Gusu School, Nanjing Medical University, Suzhou, China

[4] Department of Cardiology, Nanjing Medical University, The First Affiliated Hospital of Nanjing Medical University, Nanjing, China

[5] College of Animal Science and Technology, Yangzhou University, Yangzhou, China

[6] Hebei Technology Innovation Center of Cattle and Sheep Embryo, Baoding, China

[7] Hebei Research Institute of Dairy Industry Technology, Shijiazhuang, China

[8] Institute of Precision Medicine, The First Affiliated Hospital, Sun Yat-sen University, Guangzhou, Guangdong, China

[†]These authors contributed equally

*Correspondence: yxgaohebau@126.com, dhu.sxhu@hotmail.com, yifengwang@njmu.edu.cn & lianminchen@njmu.edu.cn

ABSTRACT

Background

The rumen of neonatal calves has limited functionality and establishing intestinal microbiota may play a crucial role in their health and performance. We aim to explore the temporal colonization of the gut microbiome and the benefits of early microbial intervention in newborn calves. We evaluated the impact of microbiota transplantation (MT) on metabolism and later milk production performance.

Results

We followed 36 newborn calves for two months and found that the composition and ecological interactions of their gut microbiomes likely reached maturity one month after birth. Temporal changes in the gut microbiome of newborn calves are widely associated with changes in their physiological statuses, such as growth and fiber digestion. Importantly, we observed that MT reshapes the gut microbiome of newborns by altering the abundance and interaction of *Bacteroides* species, as well as amino acid pathways, such as arginine biosynthesis. Two-year follow-up of those calves further showed that MT improves their later milk production. Notably, MT improves fiber digestion, antioxidant capacity of newborns while reducing diarrhea. MT also contributes to significant changes in the metabolomic landscape, and with putative causal mediation analysis, we suggest that altered gut microbial composition in newborns may influence physiological status through microbial-derived metabolites.

Conclusions

Our study provides metagenomic and metabolomic atlas of the temporal development of the gut microbiome in newborn calves. MT can alter the gut microbiome of newborns, leading to improved physiological status and later milk production. The data may help develop strategies to manipulate the gut microbiota during early life, which may be relevant to the health and production of newborn calves.

Key words: gut microbiome, metagenomics, metabolomics, neonatal calf

Data Description

We followed up on the temporal dynamics of the gut microbiome (104 samples from 3 time points) and plasma metabolome (140 samples from 4 time points) in 36 newborn calves during the first two months of life, establishing their relationships with health status and growth/production performance. The metagenomic sequencing data used for the analysis presented in this study is available from the European Nucleotide Archive (ENA) under accession id PRJEB42631. The metabolic profiles are available from the MetaboLights under accession id MTBLS7674.

Introduction

The colonization and development of the gut microbiota of newborn calves are crucial for the health and performance of the calves later in life [1]. This phenomenon is mainly attributed to the fact that these resident microbes support many functions, including the maturation of the immune system [2, 3], the utilization of nutrients [4], and the prevention of pathogen colonization [5]. Therefore, elucidating the developmental dynamics of gut microbial taxonomy and functionality during early life is important for understanding the relationships between the microbiome and host status and for eventual designing intervention strategies to achieve higher production rates and better health at later stages.

Recent studies have assessed temporal changes in the microbial taxonomic composition of newborn calves during the first week of life [6-8]. For instance, a significant increase in the relative abundance of *Lactobacillus reuteri* was observed during the first week after birth [6]. This fact seems to be very important for calf intestinal health because *L. reuteri* is known to exert bactericidal effects against bacterial pathogens and anti-infective effects against rotaviruses and *Cryptosporidium parvum in vitro* [6]. In addition, comparison of the gut microbial composition between calves (8 weeks after birth) and lactating cows showed that *Bacteroidetes* and *Verrumicrobia* were more abundant in calves, while *Firmicutes*, *Spirochaetes*, *Deinococcusthermus*, *Lentisphaerae*, *Planctomycetes*, and *Chlorofexi* were more abundant in cows [9]. These observations laid the foundation for targeted mechanistic investigations of the consequences of microbiome colonization for calf health and production.

Nevertheless, several important topics related to the temporal development of the gut microbiome in newborn calves remain unexplored. First, in addition to taxonomy, the functional composition of the gut microbiota can also undergo dynamic changes over time. Microbial functional changes, such as changes in short-chain fatty acid and amino acid metabolic pathways, due to both internal and external disruptions are implicated in the development of immunity and other systems [10, 11]. However, investigations on the temporal dynamics of gut microbial functionalities in newborn calves are still lacking. Second, the gut microbiome is an ecosystem in which microbes can compete for or exchange nutrients, signaling

molecules, or immune evasion mechanisms through complicated ecological interactions that are far from fully understood [12-14]. These interactions can be identified by co-abundance network analysis and have been shown to be related to human diseases, including obesity and inflammatory bowel diseases [15]. Investigating temporal changes in microbial interactions during the early life of newborns can enhance our understanding of gut microbial development from an ecological perspective. Third, studying the maturity of the gut microbiome in newborn calves is important because it may indicate a suitable time frame for reshaping the gut microbiome through targeted intervention before maturation. Fourth, a favorable microbiome may promote nutrient utilization and immune responses, but it is not clear whether microbial intervention during early life could improve the digestion, health status of newborn calves, as well as their later milk production performance.

To answer the above questions, we conducted the track dairy cattle study (trackDC) in which 36 newborn calves were randomly assigned into three groups: a control group, a rumen microbiota transplantation group (RMT) and an autoclaved rumen fluid transplantation group (RFT). All the newborn calves from the three groups were followed for 2 months after birth, and intensive phenotype (growth, digestion and fermentation), blood indicator, plasma metabolome and stool metagenomic analyses were conducted (**Figure S1**). In addition, the milk production performance of the calves was recorded during the two-year follow-up period. We not only investigated the temporal development of the gut microbiome at a metagenomic resolution but also evaluated whether MT could influence the phenotypes of newborn calves, including their milk production performance later in life.

Methods

Animals

The Track Dairy Cattle (trackDC) study is a longitudinal study in northern China that aims to track newborn calves to assess the development of gut microbiota during early life that contributes to cattle health and production. The study was approved by the institutional ethics review board of Hebei Agricultural University (ref. YS19003). In this study, 36 newborn calves were randomly assigned to three groups and followed for two months after birth. The groups included a control group (CON), a rumen microbiota transplantation group (RMT), and a rumen fluid transplantation group (RFT) and intensive data has been collected (**Figure S1**). The newborn calves were trained to feed milk using a bucket and then transferred to individual calf hutches. Starter was provided ad libitum three days after birth and once daily in the morning thereafter. Pasteurized whole milk was fed twice daily at 0800 and 1800 h using a bucket, and the calves were weaned 56 days after birth. RMT and RFT were performed by veterinarians, where the ruminal fluid used in RMT and RFT was collected from a healthy cattle (4-year-old, 600kg, in

the dry period,) with a permanent rumen cannula 2 hours after the morning feed. Fresh ruminal fluid was mixed with raw milk and fed to the calves in the RMT group immediately after collection. For the RFT group, the ruminal fluid was autoclaved before feeding. A volume of 50 mL, 80 mL, and 110 mL of ruminal fluid was fed from day 7 to day 11, day 21 to day 25, and day 42 to day 46, respectively. Fecal and blood samples were collected at 15, 35, and 56 days after birth. Besides, the milk production performance has also been recorded during the two-year follow-up.

Metagenomic data generation and preprocessing

Fecal samples from newborn calves were collected from rectum by stimulation of the anus and stored in liquid nitrogen after well mixed by calf. Aliquots were then made and stored at -80 °C until further processing after transferred to the laboratory. Fecal DNA isolation was performed using the QIAamp Fast DNA Stool Mini Kit (Qiagen, cat.51604). After DNA extraction, fecal DNA was used for library preparation and whole genome shotgun sequencing were performed on the Illumina NovaSeq-6000 platform. From the raw metagenomic sequencing data, low-quality reads were discarded by the sequencing facility, and reads belonging to calf and human contaminations were removed by mapping the data to the reference genomes using Bowtie2 (v.2.1.0) [16, 17]. After filtering, on average, 36.8 million (sd= 3.6 million) paired reads per sample were obtained for subsequent analysis.

Microbial taxonomies

Microbial taxonomic profiles were generated using MetaPhlAn2 (version 2.7.2) [18]. MetaPhlAn2 relies on nearly one million unique clade-specific marker genes identified from approximately 17,000 reference genomes, allowing unambiguous taxonomic assignments, accurate estimation of organismal relative abundance and species-level resolution for bacteria, archaea, eukaryotes and viruses. Microbial species present in more than 10% of the samples were included for further analyses. This yielded a list of 125 species that accounted for 99% of the original species abundance.

Microbial pathways

Microbial pathways were determined using HUMAnN2 [19]. HUMAnN2 reported the abundances of gene families from the UniProt Reference Clusters [20] (UniRef90), which were further mapped to microbial pathways from the MetaCyc metabolic pathway database [21, 22]. In total, we identified 345 pathways that were present in at least 10% of samples, retaining 100% of the original functional composition.

Microbial antibiotic resistance genes

The abundance of microbial antibiotic resistance genes in metagenomics was determined using shortBRED (version 0.9.5) [23], with markers generated from the CARD database of bacterial antibiotic resistance genes[24] (01/11/2018 version). In brief, ShortBRED is a platform for identifying a set of protein sequences from a target database (i.e., ResFinder), clustering them into families, building consensus sequences to represent the families, and then reducing these consensus sequences to a set of unique identifying strings (markers). The platform then searches for these markers in metagenomic data and determines the presence and abundance of the protein families of interest. We classified the abundance of 148 antibiotic resistance genes that were present in at least 10% of the samples.

Microbial virulence genes

The abundance of microbial virulence genes was detected using shortBRED (version 0.9.5) [23] and markers generated from virulence factors of the pathogenic bacteria database (VFDB, core dataset of DNA sequences, version: November 2018) [25]. Then, we classified the abundance of 55 virulence genes that are present in at least 10% of the samples.

Growth

The initial body weight was measured immediately after birth, and the final body weight was measured on Day 56 after birth before morning feeding. Body size, including withers height, body length, heart girth, abdominal circumference, and shank circumference, was measured on Day 0 and Day 56 after birth. The starter offered to and refused by each calf were recorded daily during the experimental period. The starter offered was collected by week and refusal was collected daily, pooled by calf weekly. Both offered and refused starter samples were oven-dried at 55 °C for 48 h weekly to determine the dry matter (DM) content. The daily starter DM intake was calculated as the difference between daily starter DM offered and starter DM refused.

Digestibility and fecal score

Feed digestibility was determined using acid detergent insoluble ash as an internal marker [26]. Briefly, fecal, starter and milk samples were collected from Day 13 to Day 15, Day 33 to Day 35 d and Day 54 to Day 56, and then, the samples from each calf were pooled, dried at 55 °C for 48 h, and then ground through a 1-mm screen for further analyses. The contents of dry matter (DM, method 930.15) and crude protein (CP, method 996.11) in the starter, milk and fecal samples were determined according to AOAC International. The contents of neutral detergent fiber (NDF) and acid detergent fiber (ADF) in the starter and feces were measured using heat stable α-amylase and sodium sulfite as described by Van Soest et al. [27]. The apparent total tract digestibility was estimated as described by Rice et al. [28]. The fecal score

was monitored and recorded once daily after morning feed on every calf, using a 4-level scoring system, as described by Larson et al. [29].

Blood biomarkers

Plasma samples were used to analyze the concentrations of blood urea nitrogen (BUN), glucose, total cholesterol and triglycerides, and serum samples were used to analyze the concentrations of total protein, albumin, alkaline phosphatase, aspartate aminotransferase (AST), alanine aminotransferase (ALT), total antioxidant capacity and malonaldehyde. All the blood biomarkers were analyzed using commercial kits from Nanjing Jiancheng Bioengineering Institute (Nanjing, China). The interassay coefficients of variation were lower than 10%, and the intra-assay coefficients of variation were lower than 12%.

Ruminal volatile fatty acids and ammonia

Ruminal fluid was collected at Day 56 using an oral stomach tube before morning feeding [30]. Ruminal pH was measured immediately after collection using a pH mater (Starter 300, Ohaus Instruments Co. Ltd., Shanghai, China). Two subsamples of 5 mL were transferred into 10 mL screw-lid centrifuge tubes after filtering through 4-layer cheesecloth. One subsample was mixed with 1 mL of 25% (wt/vol) HPO_3 for volatile fatty acid (VFA) analysis, and another subsample was mixed with 1% (wt/vol) H_2SO_4 for ammonia analysis. The concentration of ruminal VFAs was measured using gas chromatography (GC-14B, Shimadzu, Japan; 30 m × 0.32 mm × 0.25 mm; column temperature, 110 °C; injector temperature, 180 °C; and detector temperature, 180 °C) [31]. The concentration of ruminal ammonia was determined as described by Rhine et al. [32].

Un-targeted plasma metabolome

Plasma samples resuspended with prechilled 80% methanol. Then the samples were incubated on ice for 5 min and centrifuged at 15,000 g, 4°C for 20 min. The supernatant was injected into the LC-MS/MS system (a ThermoFisher Vanquish UHPLC system coupled with an Orbitrap Q ExactiveTMHF mass spectrometer). The raw data files generated by UHPLC-MS/MS were processed using the Compound Discoverer 3.1 (CD3.1, ThermoFisher) to perform peak alignment, peak picking, and quantitation for each metabolite. The normalized data was used to predict the molecular formula based on additive ions, molecular ion peaks and fragment ions. And then peaks were matched with the mzCloud (https://www.mzcloud.org/) , mzVaultand MassList database to obtain the accurate qualitative and relative quantitative results. The annotation of metabolites using the KEGG database (https://www.genome.jp/kegg/pathway.html), HMDB database (https://hmdb.ca/metabolites) and LIPIDMaps database (http://www.lipidmaps.org/).

Microbial diversity

The microbial alpha (Shannon index) and beta (Bray-Curtis dissimilarity) diversities were calculated at the species level by using the R (v3.6.0) package *vegan*.

Microbial composition dissimilarity

To compare the differences in overall microbial species composition between and within calves in each group at different time points, dimensionality reduction was carried out by using the t-distributed stochastic neighbor embedding (t-SNE) algorithm with the R package *Rtsne*. Microbiome compositional differences between groups and time points were assessed based on one the first and second t-SNE components.

Microbial species and pathway co-abundance networks

Microbial species and pathway co-abundance networks were identified by using the SparCC algorithm [33]. In detail, species composition data from MetaPhlan2 were converted to predicted read counts by multiplying relative abundances by the total sequence counts [15] and then subjected to SparCC. For pathway analysis, the read counts from HUMAnN2 cells were directly used for SparCC. Significant co-abundance was controlled at the $P< 0.01$ level using 100 times resampling.

Heterogeneity of microbial co-abundances

To assess the variability of networks between groups and different time points, we conducted Cochran-Q tests to assess the heterogeneity of effect sizes and directions for each co-abundance (correlation coefficient generated by SparCC). Here, we treated each subgroup as one study and conducted Cochran's Q test using the metagen function from the package *meta* (v4.9.5) in R, which calculates the squared difference between individual study effects and the pooled effect using inverse variance weighting [34]. For each co-abundance, the P values from the Cochran-Q test were recorded, and co-abundances with significant heterogeneity were controlled at the FDR 0.05 level determined by BH correction.

Group specific microbial co-abundances

For heterogeneous co-abundances (Cochran-Q test FDR< 0.05), we further assessed whether these relationships showed group specificity, i.e., whether the effect size of co-abundance (SparCC correlation coefficient) in one group was very different from that in the other two. We adopted interquartile ranges based on the outlier detection method [35]. The interquartile range (IQR) was calculated based on the effect size of co-abundances in each group, and we assessed whether the smallest or largest effect size fell outside of Q1-0.75IQR or Q3+0.75IQR. If only one met the condition, we called this co-abundance

specific and assigned it to the corresponding group.

Differential phenotypic, microbial and metabolic features

The relative abundances of both species and pathway datasets were centered log-ratio transformed, followed by inverse-rank transformation, before subsequent analysis [36]. No transformation was applied to phenotypic data. The ranked-based Kruskal test was then applied to assess 1) whether features within certain groups showed differences between different time points and 2) whether features within certain time points showed differences between different groups. The false discovery rate (FDR) was calculated by using the Benjamini-Hochberg (BH) method [37].

Microbial changes linked to phenotypic and metabolic changes

We calculated microbial and phenotypic changes between Day 15 and Day 35 and between Days 15 and 56. We further linked microbial changes to host phenotypic and metabolic changes with Spearman correlation. Association with $P< 0.01$ were considered significant.

Mediation linkage inference

For phenotypic and metabolic associations to the same microbial feature, we first checked whether the human phenotype was associated with the metabolite using Spearman correlation ($P<0.01$). Next, mediation analysis was carried out using the mediate function from the R package mediation (version 4.5.0) to infer the causal role of the microbiome in contributing to the host phenotype through metabolites.

Results

The Track Dairy Cattle study

To investigate the temporal development of the calf gut microbiome and the potential benefits of early microbial intervention in newborn calves, we collected fecal samples from 36 newborn calves in three groups: a control group, a rumen microbiota transplantation group (RMT), and an autoclaved rumen fluid transplantation group (RFT), as part of the Track Dairy Cattle study (trackDC). Twelve newborn calves were randomly assigned to each group, and we recorded intensive phenotypes, including growth, digestion, ruminal fermentation, and blood measurements at 1, 15, 35, and 56 days after birth (**Figure S1**, **Table S1**). We observed that 14 out of 17 traits did not exhibit differences between the groups on day 1, indicating a high degree of comparability in the baseline (**Table S1**). We also observed 29 temporal differences and three differences between the groups (FDR < 0.05, **Table S2**). For instance, the digestion rates of acid detergent fiber (ADF) and neutral detergent fiber (NDF) showed temporal differences and were significantly higher in the RMT group than in the other groups, indicating that RMT can promote the digestion of fiber by newborn calves (**Figure 1A-B**, **Table S2**). Moreover, we also observed potential beneficial effects of RMT, as the blood levels of total antioxidant capacity were the highest in the RMT group compared to the other groups (**Figure 1C**, **Table S2**). By comparing the incidence of diarrhea in the three groups, we found that the RMT group had significantly fewer cases of diarrhea (**Figure 1D**). Notably, the two-year follow-up showed that the RMT group had significantly higher milk production than the other groups (**Figure 1E**). Taken together, these results indicate the beneficial effects of early microbial interventions on the health and growth of newborn calves, as well as their later milk production performance.

Temporal variations in the gut microbial composition and ecological interaction of newborn calves

To describe the temporal development of the calf gut microbiome, we first evaluated the microbial composition and diversity. A rapid increase in the microbial alpha diversity was observed between 15 and 35 days in all three groups ($P_{\text{Kruskal test}} < 1.1\times10^{-2}$), but there was no significant difference between 35 and 56 days ($P_{\text{Kruskal test}} > 0.05$, **Figure 2A**). In addition, dimensionality reduction using the t-distributed stochastic neighbor embedding (t-SNE) algorithm further showed that the microbial composition at Day 15 was significantly different from that at Days 35 and 56 ($P_{\text{Kruskal test}} < 4.0\times10^{-5}$), while no difference was observed between Days 35 and 56 ($P_{\text{Kruskal test}} > 0.05$, **Figure 2B**).

For individual microbial species and pathways, we observed that the abundance of 65 out of 125 species (52.0%) and 174 out of 345 pathways (50.4%) were significantly different among the three time points (FDR < 0.05, Kruskal test, **Table S3–4**). Importantly, the temporal development of the gut microbiome

was mainly reflected in *Bacteroides* species and the amino acid and nucleotide biosynthesis pathways. In detail, 9 out of 65 differential species were from the genus *Bacteroides* (**Figure 2C**, **Table S3**), and 50 out of 174 pathways were related to amino acid and nucleotide biosynthesis (**Table S4**). In addition, temporal changes in 80 microbial antibiotic resistance genes and 34 virulence genes were observed (FDR< 0.05, **Table S5**). Interestingly, 57 out of 80 differential microbial antibiotic resistance genes were from *Escherichia coli* (**Table S5**), a widely recognized pathogenic species. Notably, when comparing the mean abundances of microbial species and pathways between time points, we observed that microbial species abundances at Day 35 and Day 56 were more similar than those at Day 15, while pathway abundances remained relatively stable throughout (**Figure S2**). Taken together, these results suggested that the gut microbial diversity and composition of newborn calves likely reached maturity during the first month of life.

In addition to the microbial composition, we further investigated whether microbial interactions, in terms of microbial species and pathway co-abundances, also exhibited differences between different time points in newborn calves. By using the SparCC algorithm [33], we established microbial co-abundance relationships in each subgroup separately and identified 2,393 unique species co-abundances and 38,964 pathway co-abundances ($P_{SparCC} < 0.01$, **Table S6–7**). To assess whether microbial co-abundance strengths could be different depending on the time after birth, we assessed to what extent the correlation coefficients were variable across subgroups and observed that on average, 42.3% (ranging from 40.5% to 43.3%) of the species co-abundances and 2.9% (ranging from 0.2% to 7.0%) of the pathway co-abundances showed heterogeneity between different time points (Cochran-Q test, FDR< 0.05, **Figure S3A**, **Table S6–7**). We next summarized the number of differential co-abundances between species from the same genus or from different genera (**Figure 2E**). The genus with the most heterogeneous co-abundances was *Bacteroides*, and many variable co-abundances were observed not only between different *Bacteroides* species but also between *Bacteroides* species and species from other genera such as *Alistipes* (**Figure 2E**). A similar observation was found for the pathway co-abundances, particularly for the nucleotides and amino acid biosynthesis pathways, which showed variability not only within themselves but also with respect to various pathways related to nucleotide biosynthesis (**Figure S3B**). These results indicate that the gut microbiome of newborn calves also undergoes dynamic temporal changes in species interactions after birth, while pathway interactions were relatively stable over time.

Early microbial transplantation reshapes the gut microbiome composition of newborn calves

As the gut microbial composition likely reaches maturity one month after birth, the accumulating evidence of the importance of the gut microbiota for overall newborn development indicates the need for

early modification of the microbiota. Here, we carried out RMT by the oral infusion of fresh ruminal fluid collected from healthy adult cattle. To overcome the potential bias of metabolites in ruminal fluid, which may also influence the development of gut microbiota, we included a group of calves infused with sterilized ruminal fluid. By calculating the inter-calf Bray-Curtis dissimilarity based on the abundances of all the microbial species, we observed that inter-calf dissimilarities within the RMT group were always the lowest among those of all three groups at different time points ($P_{Kruskal\ test} < 1.4\times10^{-2}$, FDR< 0.05, **Figure 3A**). This observation is proof of concept that RMT can reshape the gut microbiome composition of newborn calves, as within group gut taxonomical compositions appear more identical in the RMT group (**Figure 3A**). When comparing individual microbial species and pathways, the relative abundance of 4 species and pathways showed a significant difference at FDR < 0.05 between groups (Kruskal test, **Table S3–4**). We observed two species from *Bacteroides* genus an

d many nucleotide, amino acid and carbohydrate biosynthesis pathways (e.g., arginine, ornithine and proline interconversion and superpathway of GDP-mannose-derived O-antigen building blocks biosynthesis pathways) were differential abundant in the RMT group (FDR < 0.1, **Figure 3B-C**). Notably, differential microbial abundances between groups were mainly observed at Day 15, and most of them were driven by RMT (**Figure 3B-C**), suggesting that RMT had pronounced effects in reshaping the gut microbial composition of newborn calves in the early days of life.

Microbial interactions show specificity in the transplantation group

For microbial co-abundances, we have also checked to what extent the correlation coefficients were variable between groups, and the numbers were 40.4% (ranging from 37.1% to 43.5%) and 0.3% (ranging from 0.1% to 0.6%) for species and pathway co-abundances, respectively (Cochran-Q test, FDR< 0.05, **Figure S4A**, **Table S6–7**). These results indicate that microbial interventions can alter the interactions that may potentially contribute to the development of host phenotypes. Interestingly, heterogeneous species co-abundances characterized by comparing different groups were widely distributed in many genera (**Figure S4B**), and these co-abundances were far more complex than those characterized by comparing temporal differences between different time points (**Figure S3A**). However, this was not the case in the pathway co-abundances (**Figure S4C**).

As many of the species and pathway co-abundances showed heterogeneity between groups at different time points, we further analyzed whether those heterogeneous co-abundance relationships were driven by a particular group, i.e., whether the co-abundance strength in one group was very different from those in the other two groups at each time point. In general, we identified 633 and 101 unique group-specific species and pathway co-abundances, respectively (**Table S6–7**). Notably, 248 out of 633 (39.3%) species co-abundances and 42 out of 101 (41.6%) pathway co-abundances showed specificity for the RMT group (**Figure 4A-B**, **Table S6–7**), indicating that RMT can also alter the gut microbiome of newborn calves at the ecological level.

For those group specific co-abundances, we further evaluated potential hub species and pathways by calculating their hub score (**Table S8–9**). Interestingly, we observed a substantial amount of RMT-specific species co-abundances related to *Bifidobacterium* (**Figure 4A**), a common genus that colonized calves early in life [38, 39]. In the meanwhile, those species also have high hub scores (**Table S8**). For instance, *Bifidobacterium thermophilum* was one of the species with the most RMT-specific co-abundances (10 in total, **Table S6**), and with a high hub score of 0.98 (**Table S8**). *B. thermophilum* constitutes 80% of the infant microbiota and less than 10% of the human adult microbiota, and the presence of *Bifidobacterium* in the gut is often associated with health-promoting effects [40]. In addition,

RMT-specific pathway co-abundances mainly involved sugar derivative degradation and quinone biosynthesis pathways (**Figure 4B**), but only hexitol degradation pathway had a high hub score of 1.00 (**Table S9**). The sugar derivative degradation pathway-related co-abundances showed specificity for the RMT group, which was reasonable as we observed that the digestion rates of NDF and ADF were relatively higher in the RMT group than in the other two groups (**Figure 1A-B**).

Microbial changes associated with plasma metabolites in newborn calves

To understand the potential mechanisms by which the gut microbiota could influence host physiology, we thought that metabolites are an important class of molecules that are involved in the host-microbe interaction. By profiling plasma levels of 736 metabolites at different time points using untargeted LC-MS (**Table S10**), we observed that the plasma metabolome shifts with time in newborn calves (**Figure S5**) and 50.3% of individual metabolites (370 in total) showed significant differences between groups in at least one time point with FDR <0.05 (Kruskal test, **Table S11**).

We then checked metabolic changes specifically in relation to changes in microbial composition between Day 15 and Day 56 and between Day 15 and Day 35. In total, we observed 17,602 associations between microbial and metabolite changes, and 5,221 of them were RMT specific (Spearman correlation, $r_{absolute}$> 0.7, P< 0.01, **Figure 5A**, **Table S12–13**). Notably, various metabolites that associated with the microbiome are already known to be related to the gut microbiome, including animal essential amino acids, bile acids, organic acids and others [3]. For instance, increased abundance of microbial L-glutamate biosynthesis pathway (PWY-5505) associated with the increased levels of plasma glutathione (r_{RMT}= 0.95, P_{RMT}= 4.7×10^{-4}, **Figure 5B**), a tripeptide compound consisting of glutamate attached via its side chain to the N-terminus of cysteinyl glycine. Glutathione is an antioxidant that prevent oxidative damage through the reduction of methemoglobin and peroxides [41]. We also observed that increased abundance of microbial L-valine biosynthesis pathway (VALSYN-PWY) associated with the increased levels of plasma N-acetyl-valine (r_{RMT}= 0.79, P_{RMT}= 9.8×10^{-3}, **Figure 5C**). Valine is a branched-chain amino acid that cannot be biosynthesized by animals and plays important roles in insulin resistance and hematopoietic stem cell self-renewal [42, 43]. Taken together, those results suggested that RMT induced temporal changes of the gut microbiome in newborn calves may contribute their metabolic changes.

Microbial changes associated with the phenotypes of newborn calves

To examine the role of gut microbiota colonization in newborn calves, we explored the associations between microbial compositional changes and host phenotypes. To this end, we first calculated the microbial differences between Day 15 and Day 56 and between Day 15 and Day 35. Next, microbial

changes were associated with their corresponding phenotypic changes, including changes in growth, digestion, fermentation and blood indicators (**Table S1**). In general, we observed 381 significant associations that involved 79 species, 185 pathways and 16 phenotypes (Spearman correlation, $r_{absolute}> 0.7$, $P< 0.01$, **Table S14–15**). Interestingly, we observed that 66.7% and 50.0% differential species and pathways between groups also had at least one significant association to temporal changes in phenotypes (**Table S14–15**). Such species mainly including *Lactobacillus* (*Lactobacillus amylovorus* and *Lactobacillus reuteri*) and *Alistipes* species (*Alistipes finegoldii* and *Alistipes senegalensis*), while pathways mainly including guanosine and adenosine nucleotides, as well as L-ascorbate biosynthesis. Most of the microbial associations to host phenotypes were related to changes in serum cholesterol, total protein, globulin and albumin levels, suggesting the importance of gut microbial development to newborn calf lipid metabolism and immunity. For instance, changes in the abundance of the microbial saturated fatty acid elongation pathway were associated with changes in serum cholesterol levels ($r_{Spearman}= -0.85$, $P= 1.6\times10^{-3}$, **Table S14**). In addition, we observed that changes in serum globulin were associated with 4 prokaryotic ubiquinol biosynthesis pathways ($r_{Spearman}> 0.79$, $P< 6.1\times10^{-3}$, **Table S15**). Ubiquinols can promote coenzyme activities to enhance lipophilic antioxidants and thus simulate host immunity [44].

Notably, 77 out of 381 associations were RMT specific (i.e., only showed significance in the RMT group or change in the opposite direction when compared with other groups, $r_{absolute}> 0.7$, $P< 0.01$, **Table S14–15**), and 54 associations were related to microbial pathways (**Figure 6A-B**). Among those associations, many were related to serum aspartate transaminase (AST), malonaldehyde, total antioxidation capacity, and digestion of fiber (ADF and NDF). This suggests that RMT may potentially influence liver health, energy homeostasis, antioxidation and digestion, as reflected by changes the traits listed above (**Table S3–4**). For example, we observed that an increased abundance of microbial arginine biosynthesis was also associated with serum antioxidation capacity ($r_{Spearman}= 0.80$, $P= 1.7\times10^{-3}$, **Figure 6C**). Arginine can effectively reduce oxidative stress through the arginine/nitric oxide pathways [45]. We also observed that an increased abundance of microbial butyrate biosynthesis was associated with serum malonaldehyde levels ($r_{Spearman}= 0.79$, $P= 6.1\times10^{-3}$, **Figure 6D**). Supplementation with butyrate induced a marked shift in superoxide dismutase and catalase activities, along with a decrease in malonaldehyde levels, thereby attenuating oxidative stress [46]. These results suggest that RMT may potentially promote phenotypes of newborn calves by modulating microbial functionalities.

RMT alters phenotypic changes of newborn calves through metabolites

Since microbial changes can be linked to changes in both phenotypes and metabolites in newborn calves,

we hypothesized that microbial impacts on host phenotypes may mediate by metabolites. To evaluate whether metabolites can mediate the microbial impact on host phenotypes, we applied mediation analysis focusing on 46 microbial features that are associated with both phenotypic and metabolic changes, which revealed 40 mediation linkages ($P_{mediation} < 0.05$, **Figure 7A-B**, **Table S16–17**). Those linkages were related to microbial impact on various phenotypes including fiber digestion, antioxidant capacity, lipid and glucose metabolism via a variable category of metabolites (**Figure 7A-B**). For example, we showed that the microbial heme biosynthesis pathway may contribute to an increase in NDF digestion by increasing plasma proline-hydroxyproline levels ($P_{mediation} = 0.02$, **Figure 7C**). Proline-rich proteins were known as negative regulator that participates in modulating fiber [47]. For antioxidant capacity, we showed that the microbial purine degradation pathway may contribute to an increase in antioxidant capacity by increasing plasma thymidine levels ($P_{mediation} = 0.02$, **Figure 7D**). Thymidine catabolism may promote NADPH oxidase-derived reactive oxygen species to induce oxidative stress [48]. We also found that a Proteobacteria species *Parasutterella excrementihominis* may contribute to the decrease in plasma triglyceride by increasing plasma androsterone levels ($P_{mediation} = 0.02$, **Figure 7E**). Androsterone is an effective lipid-lowing agent [49]. In summary, these results suggest that microbial changes induced by RMT may reshape phenotypes of newborn calves through modulating host metabolism.

Discussion

A diverse microbial population colonizes the mammalian gastrointestinal tract during/after birth, and increasing evidence now suggests that this complex microbiome plays a crucial role in the development of the mucosal immune system and influences newborn health [1, 50]. Recent studies have tracked the temporal changes of the gastrointestinal microbiota in newborn calves during the first several weeks after birth at a 16S rRNA resolution, which is limited on taxonomic composition [6-8, 51]. However, the key to understanding the importance of microbial development to the host is to investigate whether within-calf microbial differences can be associated with changes in various phenotypes. We therefore systematically characterized the microbial changes at both the taxonomic and functional levels by using fecal metagenomic sequencing data from 36 newborn calves in the trackDC study.

Previous investigations on the temporal development (within 2 months) of the microbial composition of newborn calves with 16S rRNA sequencing have revealed a list of microbial genera that exhibit significant temporal differences, including *Enterococcus*, *Lactobacillus*, *Escherichia*, *Bifidobacterium*, *Clostridium*, etc. [6-8, 51]. Our in-depth metagenomic sequencing of the gut microbiome extends this observation at the species resolution, which enhances the current understanding. In addition, characterization of the temporal changes in microbial pathway abundances further showed that microbial

functionalities, such as amino acid, organic acid and carbohydrate metabolism, undergo dynamic changes in newborn calves.

This result was mainly attributed to the fact that microbe colonization of the gastrointestinal tract and their functionalities contribute to changing the ruminant digestive system from a monogastric system to a fully functional foregut rumen fermenter system, with an ability to digest fibrous feed, postweaning. The period from birth to weaning is important for rumen microbial colonization and adaptation. Once the development and maturation of the rumen and the microbiome are complete, it is difficult to permanently manipulate or change the rumen ecosystem due to microbial adaptation and resilience to external mediators [52]. In early life, a favorable microbiome can be implanted via dietary or management interventions and have potentially a long-lasting effect [1, 53, 54]. Thus, it is important to determine at what time the gut microbiome of newborn calves reaches maturity. Here, with a longitudinal study design, our data showed that the gut microbiome of newborn calves likely reaches maturity one month after birth, as indicated by the identical microbial diversity, composition and interactions between Day 35 and Day 56 after birth.

Importantly, we further showed that the gut microbiome of newborn calves can be altered by RMT at both taxonomic and functional levels, as indicated by species and pathway abundances, respectively. For instance, we observed a higher abundance of the beneficial species *Parabacteroides distasonis* in the RMT group, and this species can alleviate metabolic dysfunctions by generating succinate and secondary bile acids, which activate the intestinal gluconeogenesis pathway and farnesoid X receptor, in the gut [55]. In addition, we showed that microbial interactions in terms of co-abundances showed heterogeneity between groups and characterized many RMT-specific species and pathway co-abundances. The diverse microbial communities in the gut make up a complicated ecosystem in which microbes can exchange or compete for nutrients, signaling molecules, or immune evasion mechanisms through ecological interactions that are far from fully understood [12, 13]. Our analyses show that microbial alterations by RMT may not be driven solely by differences in abundance level; it may also reflect shifts in microbial interactions that are mirrored in co-abundance analyses. Particularly when applied to metagenomics sequence data, pathway-based co-abundance networks provide further insights into the functional alterations caused by RMT, as many RMT-specific pathway co-abundances have been identified.

Characterization of the temporal changes in the gut microbiome is crucial for understanding the role of the gut microbiome in phenotypic development of newborn calves. By linking microbial changes to phenotypic changes in newborn calves in different groups, we observed thousands of associations between the microbiome, phenotypes and metabolism, including digestion, lipid metabolism, and

immunity. Interestingly, many of those associations were only present in the RMT group. For example, RMT may increase arginine production, which further enhances the antioxidation capacity. Another example is that increased butyrate production in response to the increased amount of malonaldehyde in the RMT group. With mediation analysis by linking microbial, phenotypic and metabolic changes, our analysis suggests that microbial changes induced by RMT may reshape phenotypes of newborn calves through modulating host metabolism. Thus, our longitudinal analysis of microbial association with calf phenotypes and blood indicators revealed functional insights and putative causality of the role of the gut microbiome in newborn calf health status. These observations are of great importance for guiding further studies to develop strategies that may be used to manipulate the early microbiome to improve production and health during the time when newborn calves are most susceptible to enteric disease.

We acknowledge several limitations in the present study. Firstly, the trackDC study sampled fecal and blood samples from 36 newborn calves during the first two months after birth, making it the first metagenomics and metabolomics-based longitudinal study with the largest sample size to date. However, despite our random assignment of calves to various groups, the absence of baseline fecal microbiome profiles could potentially introduce bias to the observed differences. And the conducted analysis, such as mediation analysis may under power. Therefore, replicating these findings in independent studies with larger sample sizes like human cohort studies [56] could significantly enhance the robustness of our observations and emphasize their biological significance. Secondly, we employed various intervention volumes with the intention of mitigating potential bias arising from the natural growth of the gastrointestinal tract in newborn calves over time. However, it's important to note that this approach could introduce new biases, as we did not directly measure the actual size of their gastrointestinal tracts. Thirdly, the reported results are association-based, which means that the underlying causalities and mechanisms of action remain unexplored. Functional studies are thus essential to further reveal the underlying mechanisms of the reported associations. Finally, we primarily focused on the gut microbiome of newborn calves. However, given the fact that the temporal development of their rumen microbiome is also important but not easily sampled, further studies linking the gut and rumen microbiomes in newborn calves may provide a more systematic understanding of the importance of the microbiome in newborn calves.

Data and code availability

The metagenomic sequencing data used for the analysis presented in this study are available from the European Nucleotide Archive (ENA) under accession id PRJEB42631. The metabolic profiles are available from the MetaboLights under accession id MTBLS7674.

Code used for the analyses is publicly available via: https://github.com/MicrobiomeCardioMetaLab/trackDC.abundance_project (Licence: GPL-3).

Acknowledgements

We thank the management staff of the trackDC for their supports. This project was funded by the National Natural Science Foundation of China (NSFC, 32302756 & 32270077); The Earmarked Fund (CARS36); Hebei Dairy Cattle Innovation Team of Modern Agro-industry Technology Research System (HBCT2023180207); The Top Talent Project of Hebei Province (6012018); Precision Animal Husbandry Discipline Group Construction Project of Hebei Agricultural University (1090064); Natural Science Foundation of Hebei (C2022204248 & C2022204247); Natural Science Foundation of Jiangsu (BK20220709); Jiangsu Shuangchuang Project (Medical Expert & JSSCBS20221815); Nanjing Medical University, Changzhou Medical Centre Grant (CMCM202204); Nanjing Medical University (303073572NC21 & YNRCZN0301); Development of Jiangsu Higher Education Institutions Priority Academic Program (PAPD). The funders had no role in the study design, data collection and analysis, decision to publish, or preparation of the manuscript.

Author contributions

Y.S. and L.C. conceptualized and managed the study. Y.S., Y.L., Q.D, L.L., Y.G., Y.C., Q.L., J.S., H.Z., Y.G., L.D., J.L., Y.G., S.H., Y.W., and L.C. collected the samples and generated the data. Y.S. and L.C. analysed the data. Y.S. and L.C. drafted the manuscript. Y.S., Y.L., T.W., Q.D., Q.D, L.L., Y.G., Y.C., Q.L., J.S., H.Z., Y.G., L.D., J.L., Y.G., S.H., Y.W., and L.C. reviewed and edited the manuscript.

Competing Interests

The authors declare no competing interests.

Figure Legends

Figure 1. Temporal and microbial transplantation induced variations of phenotypes in newborn calves. A. Temporal changes of neutral detergent fiber (NDF) digestibility. P values from Kruskal test are shown accordingly. **B.** Temporal changes of acid detergent fiber (ADF) digestibility. P values from Kruskal test are shown accordingly. **C.** Temporal changes of plasma total antioxidant capacity. P values from Kruskal test are shown accordingly. **D.** Occurrence of diarrhea between groups in the whole experiential period (56 days). P values from Fisher exact test are shown accordingly. **E.** Differences in the later milk production of those calves during the two-year follow-up. P values from Wilcoxon test are shown accordingly.

Figure 2. Temporal variations of the gut microbiome in newborn calves. A. Temporal changes of the Shannon index based on species level abundance. P values from Kruskal test are shown accordingly. **B.** Temporal changes of the gut microbial composition on species level abundance. P values from Kruskal test are shown accordingly. **C.** Number of species showed temporal differences in different genus. **D.** Summary of differential microbial species co-abundances between time points. Each line represents differential species co-abundances between species from either the same or different genera. The width and darkness of the lines represent the relative number of differential co-abundances.

Figure 3. Microbiota transplantation reshapes the gut microbiome composition of newborn calves. A. Within group microbial compositional similarity. The Bray-Curtis (BC) distance represents the dissimilarity of microbial species composition between two samples. P values from Kruskal and Dunn's tests are shown. **B.** Differential species abundance between groups. The darkness of color represents the effect size of microbial abundance when comparing with the control group. P and FDR values from Kruskal and Dunn's tests are shown. **C.** Differential pathway abundance between groups. The darkness of color represents the effect size of microbial abundance when comparing with the control group. P and FDR values from Kruskal and Dunn's tests are shown.

Figure 4. Microbiota transplantation specific species and pathway co-abundances. A. 248 microbial intervention specific species co-abundances. Each dot indicates one species while each line represents a microbial intervention specific correlation between two species. Species in red are hub nodes with hub score larger than 0.5. **B.** 42 microbial intervention specific pathway co-abundances. Each dot indicates one pathway while each line represents a microbial intervention specific correlation between two pathways. Pathway in red is the hub node with hub score larger than 0.5.

Figure 5. Microbial compositional changes associated with plasma metabolic changes. A. Overview of 5,221 microbial intervention-specific microbe-metabolite associations between day 15 and day 35, day 15 and day 56. The associated microbial factors are colored gray, and the associated metabolites are colored by other colors. **B.** Positive association between plasma glutathione and microbial glutamate biosynthesis pathway changes in the microbial intervention group between day 15 and day 35. **C.** Positive association between plasma N-acetyl-valine and microbial valine biosynthesis pathway changes in the microbial intervention group between day 20 and day 35. Spearman correlation is applied to assess the associations between microbial and phenotypic changes.

Figure 6. Microbial compositional changes associated with phenotypic changes in newborn calves. A. Microbial changes between day 15 and day 56 associated with the corresponding phenotypic changes. **B.** Microbial changes between day 15 and day 35 associated with the corresponding phenotypic changes. **C.** Positive association between antioxidation capacity and microbial arginine biosynthesis pathway changes in the microbial intervention between day 15 and day 56. **D.** Positive association between blood malonaldehyde and microbial butyrate biosynthesis pathway changes in the microbial intervention group between day 15 and day 35.

Figure 7. Mediation linkages among the gut microbial, metabolic and phenotypic changes. A. Sankey plot showing the 11 significant mediation effects of plasma metabolites between day 15 and day 35. **B.** Sankey plot showing the 29 significant mediation effects of plasma metabolites between day 15 and day 56. The left panel shows the microbial factors, the middle panel shows the plasma metabolites, and the right panel shows the phenotypes. The curved lines across panels indicate the mediation effects, while the colors correspond to different phenotypes. **C.** Proline-hydroxyproline mediates the effect of microbial heme biosynthesis pathway on NDF digestion. **D.** Thymidine mediates the effect of microbial purine degradation pathway on total antioxidant capacity. **E.** Androsterone mediates the effect of *Parasutterella excrementihominis* on triglyceride.

Figure S1. Study design and overview of the data that has been collected during the first two months.

Figure S2. Comparison of microbial feature abundances between groups at different time points. Spearman correlation is applied to assess the associations of microbial abundances between different time points.

Figure S3. Temporal differences in microbial co-abundance networks. A. Summary of microbial

species and pathway co-abundances in each group at different time points. The number of significant co-abundances has been listed and the heterogeneity of co-abundances is assessed by Cochran-Q test. **B.** Summary of differential microbial pathway co-abundances between time points. Each line represents differential pathway co-abundances between pathways from either the same or different metabolic categories. The width and darkness of the lines represent the relative number of differential co-abundances.

Figure S4. Differential microbial co-abundance networks between groups. A. Summary of microbial species and pathway co-abundances in each group at different time points. The number of significant co-abundances has been listed and the heterogeneity of co-abundances is assessed by Cochran-Q test. **B.** Summary of differential microbial species co-abundances between groups. Each line represents differential species co-abundances between species from either the same or different genera. The width and darkness of the lines represent the relative number of differential co-abundances. **C.** Summary of differential microbial pathway co-abundances between groups. Each line represents differential pathway co-abundances between pathways from either the same or different metabolic categories. The width and darkness of the lines represent the relative number of differential co-abundances.

Figure S5. The composition of plasma metabolites shows temporal and group differences. P values from Kruskal test are shown accordingly.

Table Legends

Table S1. Summary of phenotypes collected in this study

Table S2. Phenotypic differences between groups and time points

Table S3. Differential microbial species abundances between groups and time points

Table S4. Differential microbial pathway abundances between groups and time points

Table S5. Differential microbial antibiotic resistance and virulence gene abundances between groups and time points

Table S6. Microbial species co-abundances

Table S7. Microbial pathway co-abundances

Table S8. Hub scores of species in co-abundance networks

Table S9. Hub scores of pathways in co-abundance networks

Table S10. Summary of plasma metabolome

Table S11. Differential plasma metabolites between groups

Table S12. Microbial changes between day15 and day35 associated with metabolic changes

Table S13. Microbial changes between day15 and day56 associated with metabolic changes

Table S14. Microbial changes between day15 and day35 associated with phenotypic changes

Table S15. Microbial changes between day15 and day56 associated with phenotypic changes

Table S16. Metabolites mediate microbial impacts on phenotypic changes between day15 and day35

Table S17. Metabolites mediate microbial impacts on phenotypic changes between day15 and day56

References

1. Arshad MA, Hassan F-u, Rehman MS, Huws SA, Cheng Y, Din AU: **Gut microbiome colonization and development in neonatal ruminants: Strategies, prospects, and opportunities.** *Animal Nutrition* 2021.
2. Thaiss CA, Zmora N, Levy M, Elinav E: **The microbiome and innate immunity.** *Nature* 2016, **535:**65-74.
3. Wang Y, Dong Q, Hu S, Zou H, Wu T, Shi J, Zhang H, Sheng Y, Sun W, Kong X: **Decoding microbial genomes to understand their functional roles in human complex diseases.** *iMeta* 2022**:**e14.
4. Nicholson JK, Holmes E, Kinross J, Burcelin R, Gibson G, Jia W, Pettersson S: **Host-gut microbiota metabolic interactions.** *Science* 2012, **336:**1262-1267.
5. Buffie CG, Pamer EG: **Microbiota-mediated colonization resistance against intestinal pathogens.** *Nat Rev Immunol* 2013, **13:**790-801.
6. Schwaiger K, Storch J, Bauer C, Bauer J: **Development of selected bacterial groups of the rectal microbiota of healthy calves during the first week postpartum.** *J Appl Microbiol* 2020, **128:**366-375.
7. Takino T, Kato-Mori Y, Motooka D, Nakamura S, Iida T, Hagiwara K: **Postnatal changes in the relative abundance of intestinal Lactobacillus spp. in newborn calves.** *J Vet Med Sci* 2017, **79:**452-455.
8. Song Y, Malmuthuge N, Steele MA, Guan LL: **Shift of hindgut microbiota and microbial short chain fatty acids profiles in dairy calves from birth to pre-weaning.** *FEMS Microbiol Ecol* 2018, **94**.
9. Haley BJ, Kim SW, Salaheen S, Hovingh E, Van Kessel JAS: **Differences in the Microbial Community and Resistome Structures of Feces from Preweaned Calves and Lactating Dairy Cows in Commercial Dairy Herds.** *Foodborne Pathog Dis* 2020, **17:**494-503.
10. Yang W, Yu T, Huang X, Bilotta AJ, Xu L, Lu Y, Sun J, Pan F, Zhou J, Zhang W, et al: **Intestinal microbiota-derived short-chain fatty acids regulation of immune cell IL-22 production and gut immunity.** *Nat Commun* 2020, **11:**4457.
11. Visconti A, Le Roy CI, Rosa F, Rossi N, Martin TC, Mohney RP, Li W, de Rinaldis E, Bell JT, Venter JC, et al: **Interplay between the human gut microbiome and host metabolism.** *Nat Commun* 2019, **10:**4505.
12. Baumler AJ, Sperandio V: **Interactions between the microbiota and pathogenic**

bacteria in the gut. *Nature* 2016, **535:**85-93.
13. Whiteley M, Diggle SP, Greenberg EP: **Progress in and promise of bacterial quorum sensing research.** *Nature* 2017, **551:**313-320.
14. Chen L, Garmaeva S, Zhernakova A, Fu J, Wijmenga C: **A system biology perspective on environment-host-microbe interactions.** *Hum Mol Genet* 2018, **27:**R187-R194.
15. Chen L, Collij V, Jaeger M, van den Munckhof ICL, Vich Vila A, Kurilshikov A, Gacesa R, Sinha T, Oosting M, Joosten LAB, et al: **Gut microbial co-abundance networks show specificity in inflammatory bowel disease and obesity.** *Nature Communications* 2020, **11:**4018.
16. Langmead B, Trapnell C, Pop M, Salzberg SL: **Ultrafast and memory-efficient alignment of short DNA sequences to the human genome.** *Genome Biol* 2009, **10:**R25.
17. Langmead B, Wilks C, Antonescu V, Charles R: **Scaling read aligners to hundreds of threads on general-purpose processors.** *Bioinformatics* 2019, **35:**421-432.
18. Truong DT, Franzosa EA, Tickle TL, Scholz M, Weingart G, Pasolli E, Tett A, Huttenhower C, Segata N: **MetaPhlAn2 for enhanced metagenomic taxonomic profiling.** *Nat Methods* 2015, **12:**902-903.
19. Franzosa EA, McIver LJ, Rahnavard G, Thompson LR, Schirmer M, Weingart G, Lipson KS, Knight R, Caporaso JG, Segata N, Huttenhower C: **Species-level functional profiling of metagenomes and metatranscriptomes.** *Nature Methods* 2018, **15:**962-968.
20. Bateman A, Martin MJ, O'Donovan C, Magrane M, Apweiler R, Alpi E, Antunes R, Ar-Ganiska J, Bely B, Bingley M, et al: **UniProt: a hub for protein information.** *Nucleic Acids Research* 2015, **43:**D204-D212.
21. Caspi R, Billington R, Ferrer L, Foerster H, Fulcher CA, Keseler IM, Kothari A, Krummenacker M, Latendresse M, Mueller LA, et al: **The MetaCyc database of metabolic pathways and enzymes and the BioCyc collection of pathway/genome databases.** *Nucleic Acids Research* 2016, **44:**D471-D480.
22. Caspi R, Billington R, Fulcher CA, Keseler IM, Kothari A, Krummenacker M, Latendresse M, Midford PE, Ong Q, Ong WK, et al: **The MetaCyc database of metabolic pathways and enzymes.** *Nucleic Acids Research* 2018, **46:**D633-D639.
23. Kaminski J, Gibson MK, Franzosa EA, Segata N, Dantas G, Huttenhower C: **High-Specificity Targeted Functional Profiling in Microbial Communities with**

ShortBRED. *PLoS Comput Biol* 2015, **11:**e1004557.

24. Jia B, Raphenya AR, Alcock B, Waglechner N, Guo P, Tsang KK, Lago BA, Dave BM, Pereira S, Sharma AN, et al: **CARD 2017: expansion and model-centric curation of the comprehensive antibiotic resistance database.** *Nucleic Acids Res* 2017, **45:**D566-D573.
25. Liu B, Zheng D, Jin Q, Chen L, Yang J: **VFDB 2019: a comparative pathogenomic platform with an interactive web interface.** *Nucleic Acids Res* 2019, **47:**D687-D692.
26. Li Y, Shen Y, Niu J, Guo Y, Pauline M, Zhao X, Li Q, Cao Y, Bi C, Zhang X, et al: **Effect of active dry yeast on lactation performance, methane production, and ruminal fermentation patterns in early-lactating Holstein cows.** *J Dairy Sci* 2021, **104:**381-390.
27. Van Soest PJ, Robertson JB, Lewis BA: **Methods for dietary fiber, neutral detergent fiber, and nonstarch polysaccharides in relation to animal nutrition.** *J Dairy Sci* 1991, **74:**3583-3597.
28. Rice EM, Aragona KM, Moreland SC, Erickson PS: **Supplementation of sodium butyrate to postweaned heifer diets: Effects on growth performance, nutrient digestibility, and health.** *J Dairy Sci* 2019, **102:**3121-3130.
29. Larson LL, Owen FG, Albright JL, Appleman RD, Lamb RC, Muller LD: **Guidelines toward more uniformity in measuring and reporting calf experimental data.** *Journal of Dairy Science* 1977, **60:**989-991.
30. Shen JS, Chai Z, Song LJ, Liu JX, Wu YM: **Insertion depth of oral stomach tubes may affect the fermentation parameters of ruminal fluid collected in dairy cows.** *Journal of Dairy Science* 2012, **95:**5978-5984.
31. Shen YZ, Ding LY, Chen LM, Xu JH, Zhao R, Yang WZ, Wang HR, Wang MZ: **Feeding corn grain steeped in citric acid modulates rumen fermentation and inflammatory responses in dairy goats.** *Animal* 2019, **13:**301-308.
32. Rhine ED, Sims GK, Mulvaney RL, Pratt EJ: **Improving the Berthelot reaction for determining ammonium in soil extracts and water.** *Soil Science Society of America Journal* 1998, **62:**473-480.
33. Friedman J, Alm EJ: **Inferring Correlation Networks from Genomic Survey Data.** *Plos Computational Biology* 2012, **8**.
34. Schwarzer G: **meta: An R package for meta-analysis.** *R News* 2007, **7:**6.
35. Barbato G, Barini EM, Genta G, Levi R: **Features and performance of some outlier**

detection methods. *Journal of Applied Statistics* 2011, **38:**2133-2149.

36. Aitchison J: **The statistical analysis of compositional data.** *Journal of the Royal Statistical Society: Series B (Methodological)* 1982, **44:**139-160.

37. Benjamini Y, Hochberg Y: **Controlling the False Discovery Rate - a Practical and Powerful Approach to Multiple Testing.** *Journal of the Royal Statistical Society Series B-Methodological* 1995, **57:**289-300.

38. Yassour M, Jason E, Hogstrom LJ, Arthur TD, Tripathi S, Siljander H, Selvenius J, Oikarinen S, Hyoty H, Virtanen SM, et al: **Strain-Level Analysis of Mother-to-Child Bacterial Transmission during the First Few Months of Life.** *Cell Host Microbe* 2018, **24:**146-154.

39. Gacesa R, Kurilshikov A, Vich Vila A, Sinha T, Klaassen MAY, Bolte LA, Andreu-Sánchez S, Chen L, Collij V, Hu S, et al: **The Dutch Microbiome Project defines factors that shape the healthy gut microbiome.** *bioRxiv* 2020**:**2020.2011.2027.401125.

40. Hussein L, Singh RB: **Developments on the Applications and the Suitability of Functional Fermented Sour Sobya as a Viable Source of Novel Probiotics in the Managements of Gastrointestinal Disorders and Blood Lipid Profiles.** In *The Role of Functional Food Security in Global Health.* Elsevier; 2019: 579-602

41. Gaucher C, Boudier A, Bonetti J, Clarot I, Leroy P, Parent M: **Glutathione: Antioxidant Properties Dedicated to Nanotechnologies.** *Antioxidants (Basel)* 2018, **7.**

42. Cummings NE, Williams EM, Kasza I, Konon EN, Schaid MD, Schmidt BA, Poudel C, Sherman DS, Yu D, Arriola Apelo SI, et al: **Restoration of metabolic health by decreased consumption of branched-chain amino acids.** *J Physiol* 2018, **596:**623-645.

43. Taya Y, Ota Y, Wilkinson AC, Kanazawa A, Watarai H, Kasai M, Nakauchi H, Yamazaki S: **Depleting dietary valine permits nonmyeloablative mouse hematopoietic stem cell transplantation.** *Science* 2016, **354:**1152-1155.

44. Zhang Y, Liu J, Chen X-q, Chen C-YO: **Ubiquinol is superior to ubiquinone to enhance Coenzyme Q10 status in older men.** *Food & function* 2018, **9:**5653-5659.

45. Shan LL, Wang B, Gao GZ, Cao WG, Zhang YK: **L-Arginine supplementation improves antioxidant defenses through L-arginine/nitric oxide pathways in exercised rats.** *Journal of Applied Physiology* 2013, **115:**1146-1155.

46. Zhou Y, Ji XW, Chen JJ, Fu YY, Huang JW, Guo R, Zhou JH, Cen JK, Zhang QH, Chu AN, et al: **Short-chain fatty acid butyrate: A novel shield against chronic gastric ulcer.** *Experimental and Therapeutic Medicine* 2021, **21**.

47. Xu WL, Zhang DJ, Wu YF, Qin LX, Huang GQ, Li J, Li L, Li XB: **Cotton PRP5 gene encoding a proline-rich protein is involved in fiber development.** *Plant Mol Biol* 2013, **82:**353-365.

48. Tabata S, Yamamoto M, Goto H, Hirayama A, Ohishi M, Kuramoto T, Mitsuhashi A, Ikeda R, Haraguchi M, Kawahara K, et al: **Thymidine catabolism promotes NADPH oxidase-derived reactive oxygen species (ROS) signalling in KB and yumoto cells.** *Sci Rep* 2018, **8:**6760.

49. Oliver MF: **Reduction of serum-lipid and uric-acid levels by an orally active androsterone.** *Lancet* 1962, **1:**1321-1323.

50. Malmuthuge N, Griebel PJ, Guan le L: **The Gut Microbiome and Its Potential Role in the Development and Function of Newborn Calf Gastrointestinal Tract.** *Front Vet Sci* 2015, **2:**36.

51. Kim ET, Lee SJ, Kim TY, Lee HG, Atikur RM, Gu BH, Kim DH, Park BY, Son JK, Kim MH: **Dynamic Changes in Fecal Microbial Communities of Neonatal Dairy Calves by Aging and Diarrhea.** *Animals (Basel)* 2021, **11**.

52. Clemmons BA, Voy BH, Myer PR: **Altering the Gut Microbiome of Cattle: Considerations of Host-Microbiome Interactions for Persistent Microbiome Manipulation.** *Microb Ecol* 2019, **77:**523-536.

53. Palma-Hidalgo JM, Jimenez E, Popova M, Morgavi DP, Martin-Garcia AI, Yanez-Ruiz DR, Belanche A: **Inoculation with rumen fluid in early life accelerates the rumen microbial development and favours the weaning process in goats.** *Anim Microbiome* 2021, **3:**11.

54. Malmuthuge N, Liang GX, Guan LL: **Regulation of rumen development in neonatal ruminants through microbial metagenomes and host transcriptomes.** *Genome Biology* 2019, **20**.

55. Wang K, Liao M, Zhou N, Bao L, Ma K, Zheng Z, Wang Y, Liu C, Wang W, Wang J, et al: **Parabacteroides distasonis Alleviates Obesity and Metabolic Dysfunctions via Production of Succinate and Secondary Bile Acids.** *Cell Rep* 2019, **26:**222-235 e225.

56. Sun W, Wang Y, Zhang H, Sheng Y, Fan J, Gu M, Tian Y, Zhang Y, Ma H, Yin X:

eHypertension: A prospective longitudinal multi- omics essential hypertension cohort. Wiley Online Library; 2022.

Figure1

Click here to access/download;Figure;Figure1.pdf

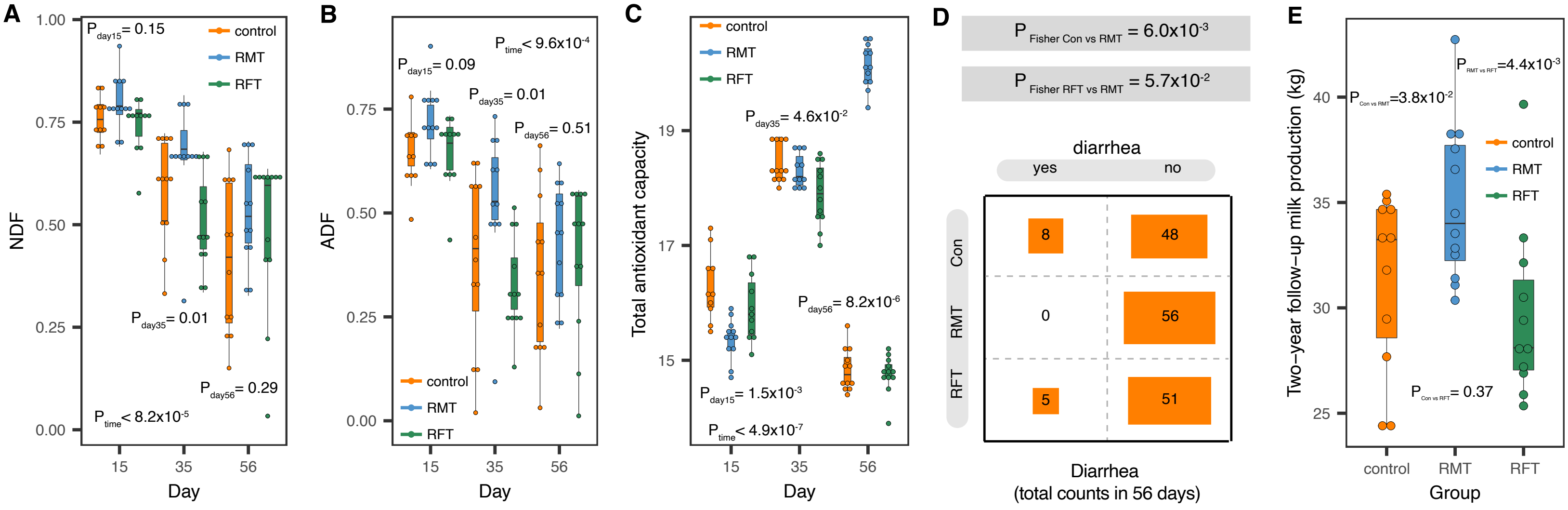

Figure2 Click here to access/download;Figure;Figure2.pdf

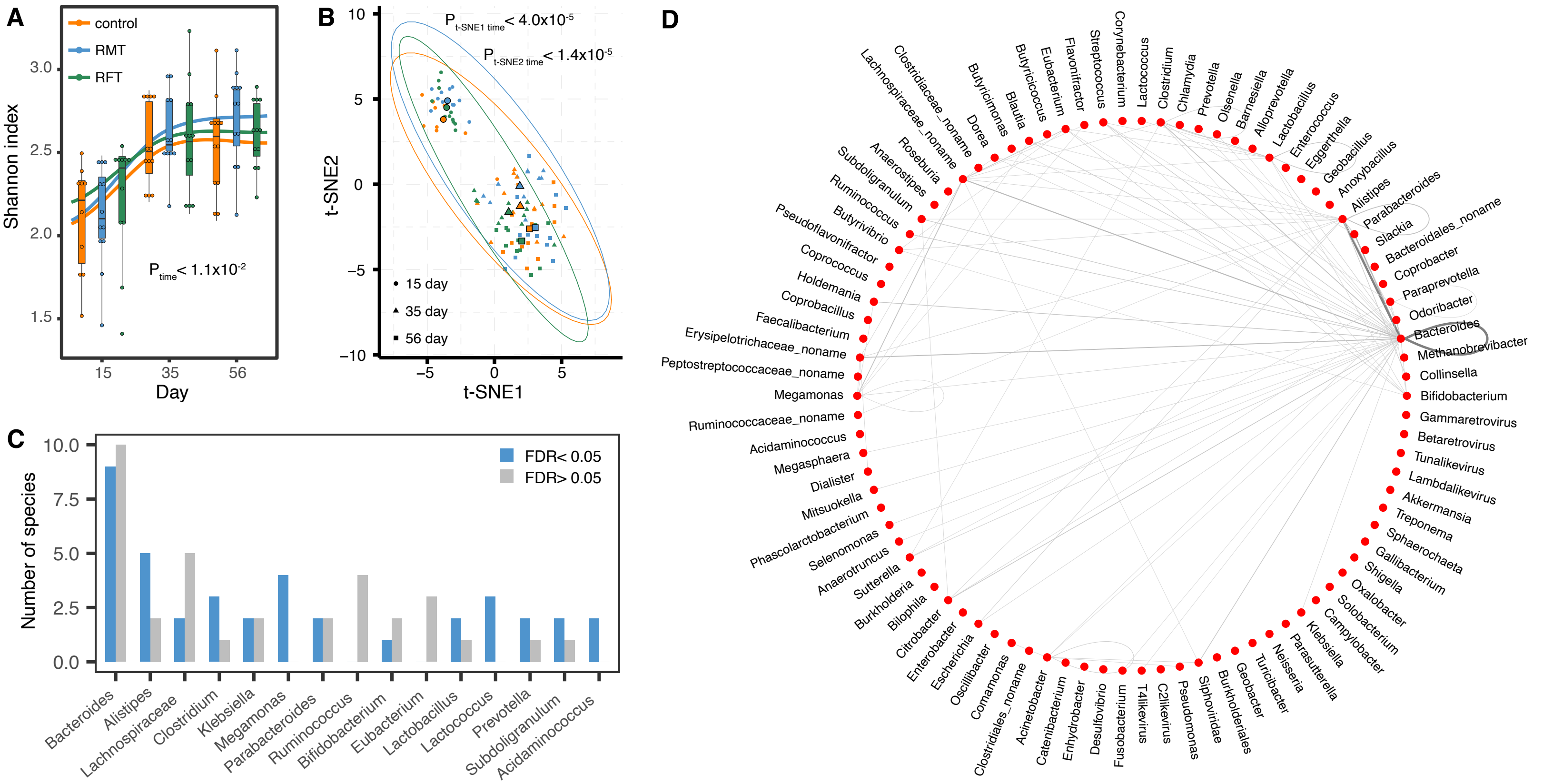

Figure3

Click here to access/download;Figure;Figure3.pdf

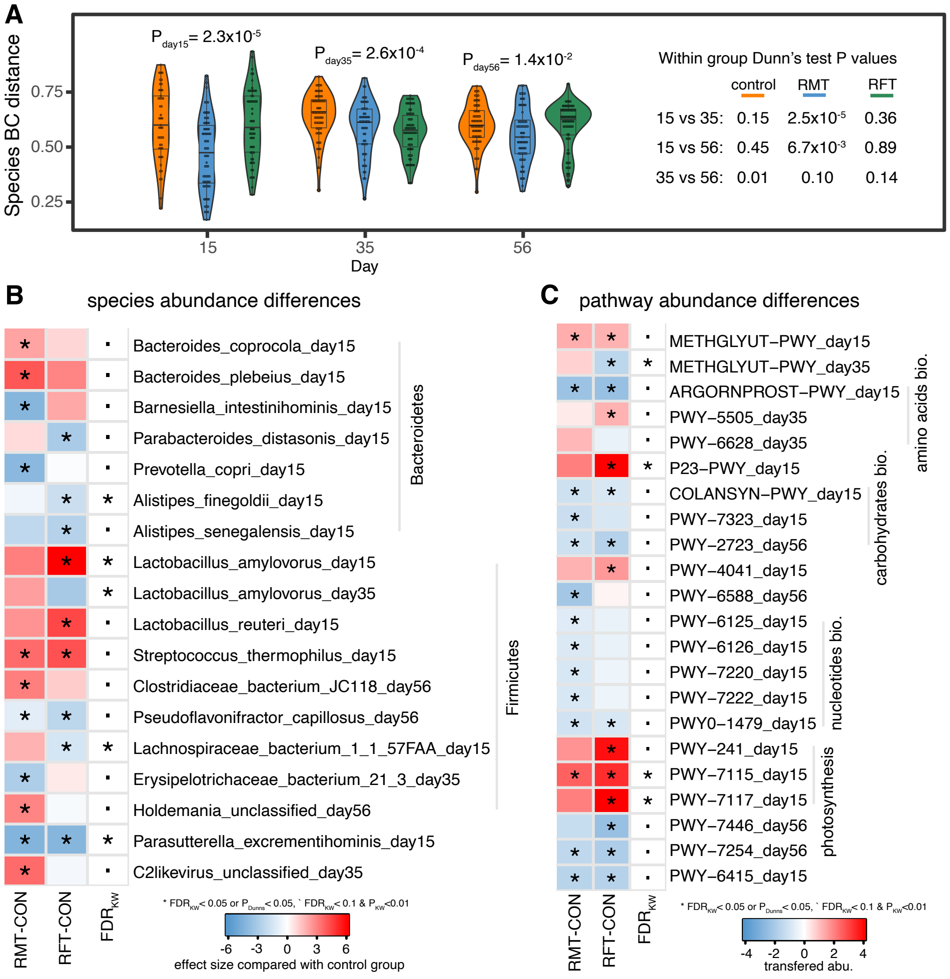

Figure4

Click here to access/download;Figure;Figure4.pdf

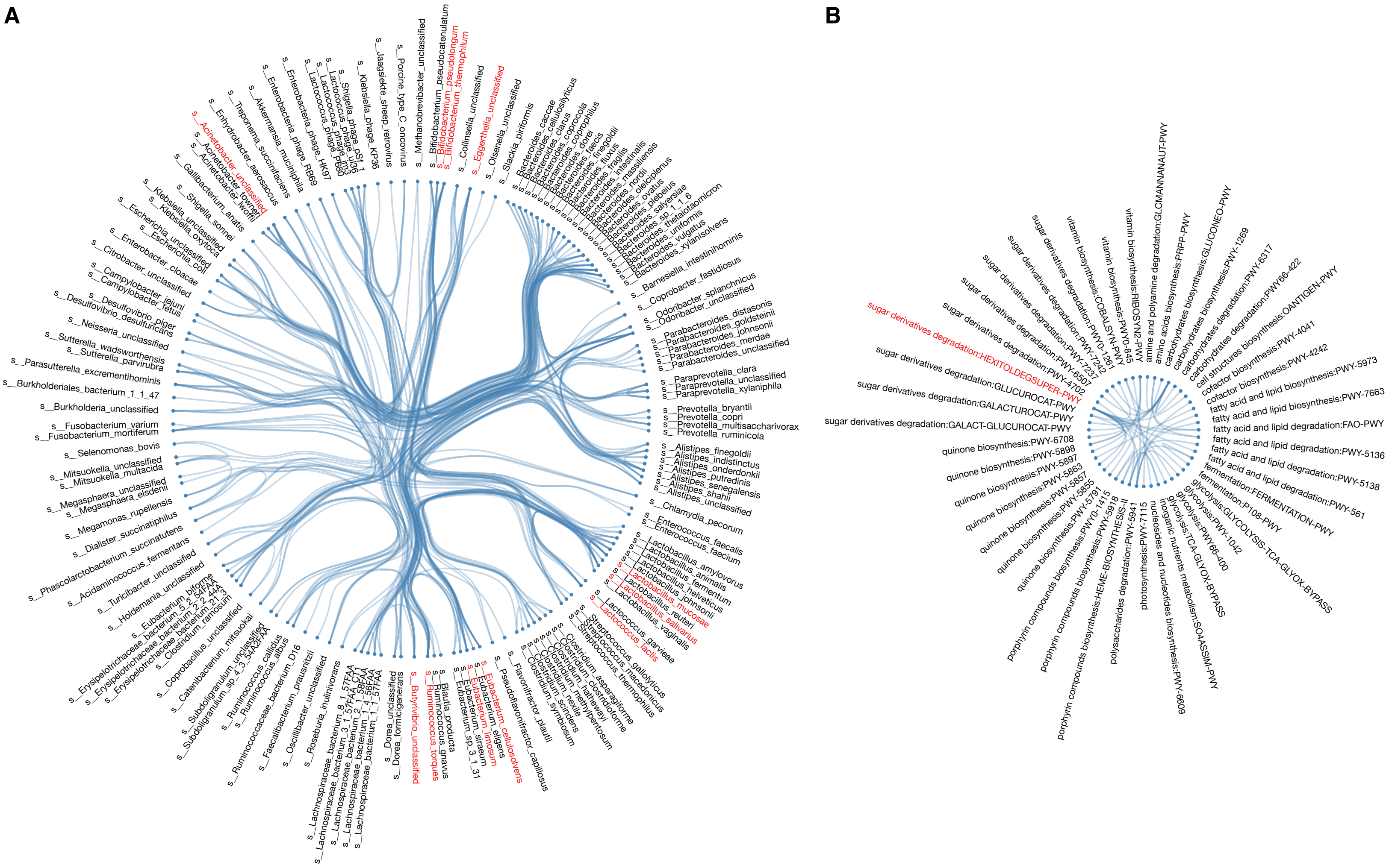

Figure5

Click here to access/download;Figure;Figure5.pdf

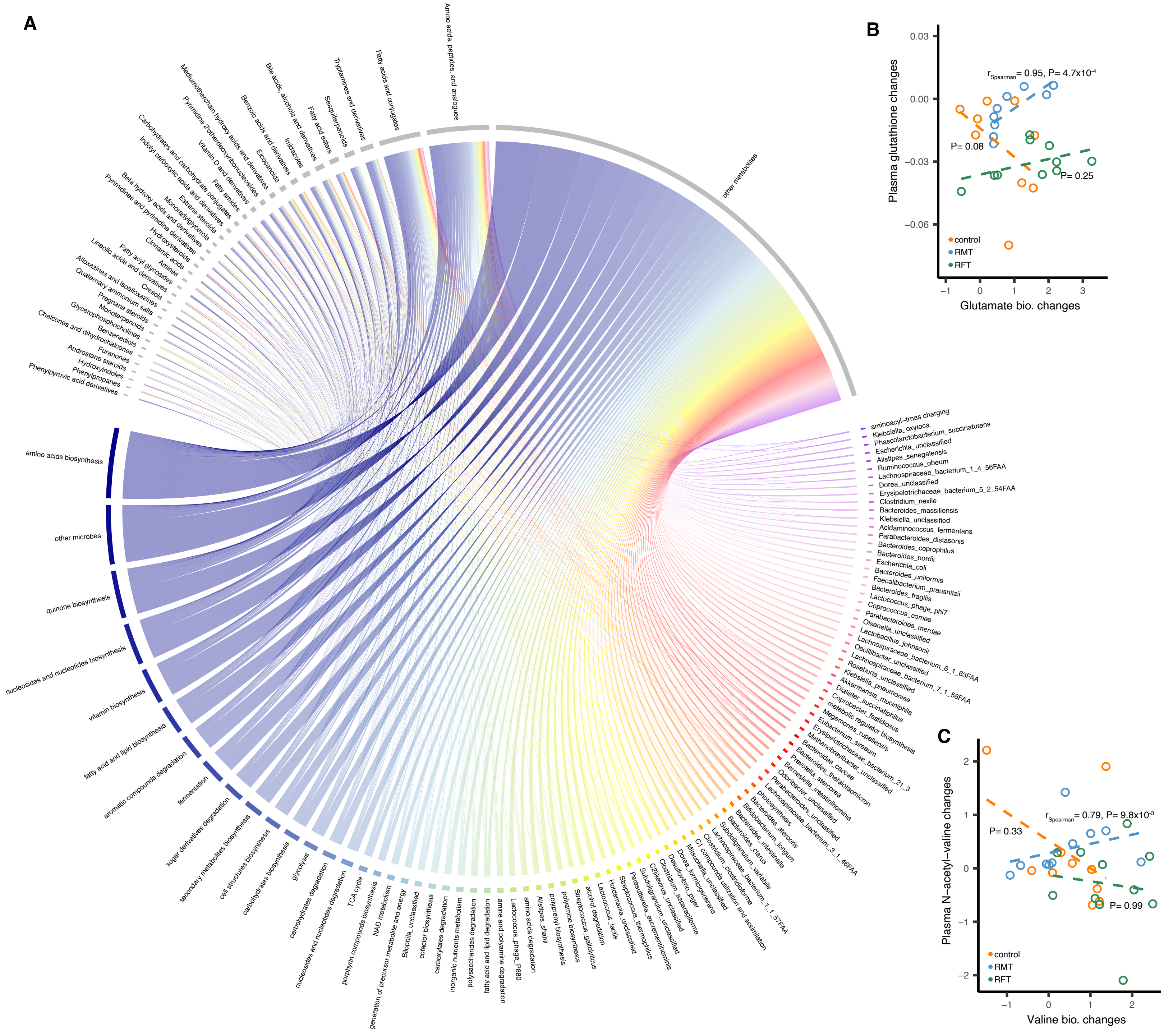

Figure6 Click here to access/download;Figure;Figure6.pdf

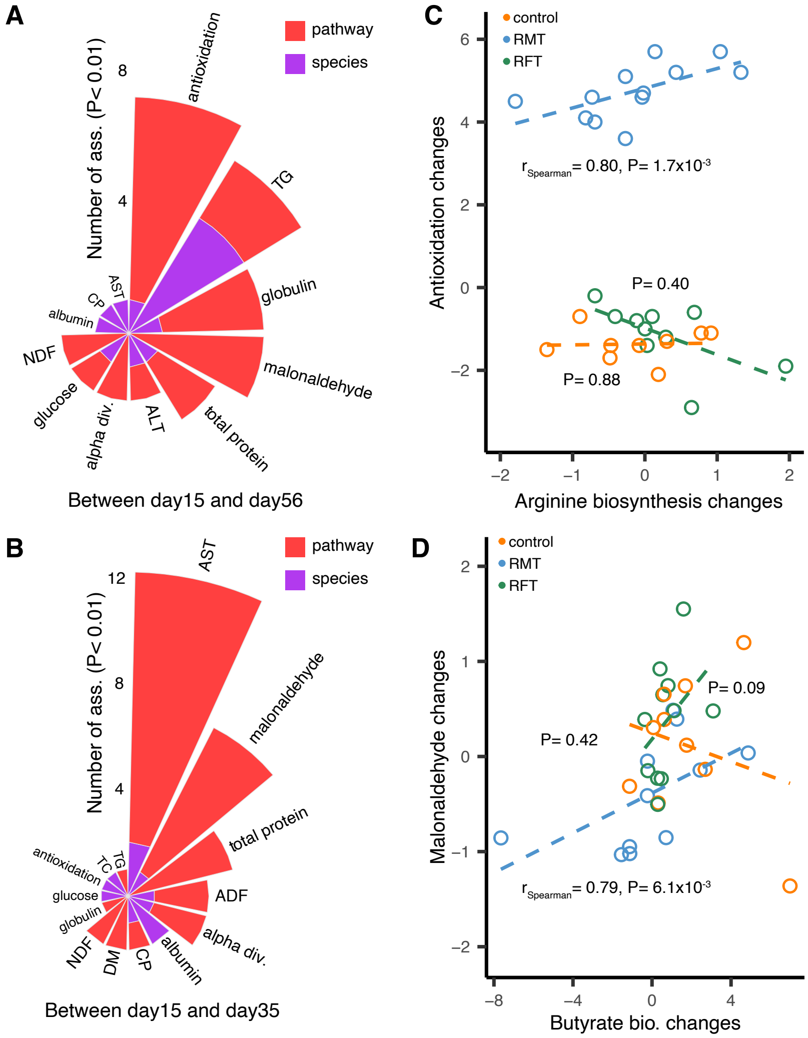

Figure7 Click here to access/download;Figure;Figure7.pdf

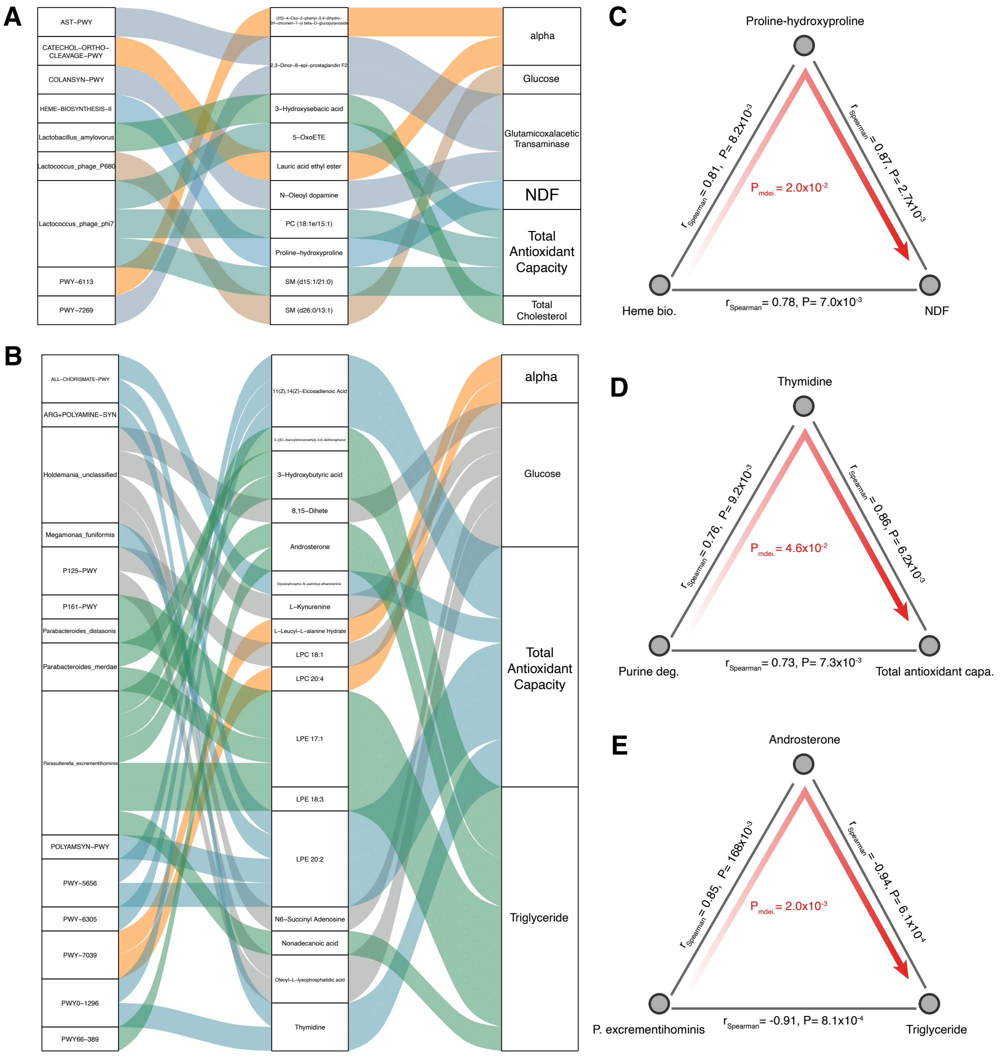

FigureS1

Click here to access/download
Supplementary Material
FigureS1.pdf

FigureS2

Click here to access/download
Supplementary Material
FigureS2.pdf

FigureS3

Click here to access/download
Supplementary Material
FigureS3.pdf

FigureS4

Click here to access/download
Supplementary Material
FigureS4.pdf

FigureS5

Click here to access/download
Supplementary Material
FigureS5.pdf

Click here to access/download
Supplementary Material
SuppTables.xlsx
