# Supplementary material for: Early microbial intervention reshapes phenotypes of newborn Bos taurus through metabolic regulations
Source: Gigascience. 2024 Jan 13;13:giad118. doi: 10.1093/gigascience/giad118 (PMC10787367; doi:10.1093/gigascience/giad118)
Supplement: giad118_GIGA-D-23-00086_Revision_2 [file giad118_giga-d-23-00086_revision_2.pdf]

GigaScience

Early microbial intervention reshapes phenotypes of newborn Bos taurus through metabolic regulations

--Manuscript Draft--

Manuscript Number:	GIGA-D-23-00086R2
Full Title:	Early microbial intervention reshapes phenotypes of newborn Bos taurus through metabolic regulations
Article Type:	Research
Funding Information:	
Abstract:	Background<br><br>The rumen of neonatal calves has limited functionality and establishing intestinal microbiota may play a crucial role in their health and performance. We aim to explore the temporal colonization of the gut microbiome and the benefits of early microbial intervention in newborn calves. We evaluated the impact of microbiota transplantation (MT) on metabolism and later milk production performance.<br><br>Results<br><br>We followed 36 newborn calves for two months and found that the composition and ecological interactions of their gut microbiomes likely reached maturity one month after birth. Temporal changes in the gut microbiome of newborn calves are widely associated with changes in their physiological statuses, such as growth and fiber digestion. Importantly, we observed that MT reshapes the gut microbiome of newborns by altering the abundance and interaction of Bacteroides species, as well as amino acid pathways, such as arginine biosynthesis. Two-year follow-up of those calves further showed that MT improves their later milk production. Notably, MT improves fiber digestion, antioxidant capacity of newborns while reducing diarrhea. MT also contributes to significant changes in the metabolomic landscape, and with putative causal mediation analysis, we suggest that altered gut microbial composition in newborns may influence physiological status through microbial-derived metabolites.<br><br>Conclusions<br><br>Our study provides metagenomic and metabolomic atlas of the temporal development of the gut microbiome in newborn calves. MT can alter the gut microbiome of newborns, leading to improved physiological status and later milk production. The data may help develop strategies to manipulate the gut microbiota during early life, which may be relevant to the health and production of newborn calves.
Corresponding Author:	Lianmin Chen, Ph.D.<br>Nanjing Medical University<br>Nanjing, Jiangsu CHINA
Corresponding Author Secondary Information:	
Corresponding Author's Institution:	Nanjing Medical University
Corresponding Author's Secondary Institution:	
First Author:	Yizhao Shen
First Author Secondary Information:	
Order of Authors:	Yizhao Shen
	Yan Li
	Tingting Wu
	Quanbin Dong

Powered by Editorial Manager® and ProduXion Manager® from Aries Systems Corporation

	Qiufeng Deng
	Lu Liu
	Yanfei Guo
	Yufeng Cao
	Qiufeng Li
	Jing Shi
	Huayiyang Zou
	Yuwen Jiao
	Luoyang Ding
	Jianguo Li
	Yanxia Gao
	Shixian Hu
	Yifeng Wang
	Lianmin Chen
**Order of Authors Secondary Information:**	
**Response to Reviewers:**	Detailed reply has been attached.
**Additional Information:**	
**Question**	**Response**
Are you submitting this manuscript to a special series or article collection?	No
**Experimental design and statistics**<br><br>Full details of the experimental design and statistical methods used should be given in the Methods section, as detailed in our Minimum Standards Reporting Checklist. Information essential to interpreting the data presented should be made available in the figure legends.<br><br>Have you included all the information requested in your manuscript?	Yes
**Resources**<br><br>A description of all resources used, including antibodies, cell lines, animals and software tools, with enough information to allow them to be uniquely identified, should be included in the Methods section. Authors are strongly encouraged to cite Research Resource	Yes

Identifiers (RRIDs) for antibodies, model organisms and tools, where possible.<br><br>Have you included the information requested as detailed in our Minimum Standards Reporting Checklist?	
**Availability of data and materials**<br><br>All datasets and code on which the conclusions of the paper rely must be either included in your submission or deposited in publicly available repositories (where available and ethically appropriate), referencing such data using a unique identifier in the references and in the "Availability of Data and Materials" section of your manuscript.<br><br>Have you have met the above requirement as detailed in our Minimum Standards Reporting Checklist?	Yes

Rebuttal
Click here to access/download;Manuscript;reviewer_comments.doc

Reply to comments raised by reviewers:

Reviewer1:

The authors have made substantial improvements in addressing most of my previous comments in the revised manuscript. However, there are still a few minor typos and questions that need to be addressed.

Reply: Thanks, now we have further addressed those mistakes. For details, please see below.

1) Figure 2 (line 547-553)
The data presented on 2C and 2D were at the genus level but not the species level.
Please revise the legends of panel C and D, and also legends for all main/sup figures.

Reply: Apologies for the confusion. Figures 2C and D indeed represent the summary of differential species within specific genera and the differential species co-abundances at the genus level resolution. We have added additional details to the figure legends to clarify this and avoid any misunderstandings. Additionally, we have thoroughly reviewed and revised all main and supplementary figures along with their legends.

2) Figure 3C
Please modify the color key as it does not represent the transformed abundances but the effect size). Please also add the full name of each pathway as readers could hardly get useful information from the pathway IDs. e.g., PWY-6588 -pyruvate fermentation to acetone.

Reply: Thanks, we have corrected the typos. However, the full names of the pathways are too lengthy to fit within the figure. Therefore, we have included the complete pathway names in the figure legends.

Line 68-70. The authors mentioned that "In addition, comparison of the gut microbial composition between calves (8 weeks after birth) and lactating cows showed that Bacteroidetes and Verrumicrobia were more abundant in calves, while Firmicutes, Spirochaetes, Deinococcusthermus, Lentisphaerae, Planctomycetes, and Chlorofexi were more abundant in cows [9]." There is a lack of their own data to generally describe the gut microbial composition of calves at different time points (birth~8 weeks). For instance, the top 5 phyla, as well as the top 10/20 abundant genera at different time points across different groups. Any there any differences /similarity between their results and previous results sampling after 8 weeks?

Reply: We observed that the abundance of 29 species increased with time (P<0.05), including 5 species from *Bacteroides*, 3 species from *Alistipes*, and 3 species from *Lachnospiraceae*. Meanwhile, the abundance of 23 species decreased with time (P<0.05), including 4 species from *Clostridium*, 3 species from *Bacteroides*, and 3 species from *Megamonas* (**Table S3**). This information has now been included in the main text (**L288**).
We concur with the reviewer's observation that our study lacks microbiome profile data beyond the 8-week mark. Comparison with existing studies, primarily from rumen samples, does show some consistency in the top abundant phyla, including Firmicutes, Proteobacteria, and Euryarchaeota. However, due to the absence of gut microbiome profiles from later years, we cannot conclusively assert that the gut microbiome of these newborn calves is similar to that of adult cows. This limitation is also pertinent to the concerns raised by the reviewer (see answers to below questions).

In this study, the authors examined the gut microbial differences between RMT, RFT and controls of newborn calves and reported that their gut microbiomes likely reached maturity one month after birth". However, all of the samples collected in this study are pre-weaning calves. The authors also stated in the discussion that "The period from birth to weaning is important for rumen microbial colonization and adaptation". Likewise, breastfeed duration has shown strong impacts in shaping the gut microbiota of human infants. Thus, it is still unknown whether the gut microbiota of calves will further develop after weaning (possibly Yes). In this study, the authors examined the gut microbial differences between RMT, RFT, and control groups of newborn calves. They reported that the gut microbiomes of these

calves likely reached maturity one month after birth. However, it's important to note that all samples collected in this study are from pre-weaning calves. In the discussion, the authors pointed the significance of the period from birth to weaning in terms of rumen microbial colonization and adaptation. Similarly, the duration of breastfeeding has been shown to strongly impacts on the gut microbiota of human infants, particularly on the Bifidobacterium members. However, it remains uncertain whether the gut microbiota of calves will continue to evolve after weaning. This question warrants further investigation. The authors should carefully to state with "maturity".

Reply: Thank you for bringing this to our attention. We completely agree with the reviewer that the original claim stating 'the gut microbiome of newborn calves likely reaches maturity two months after birth' lacked rigor and supporting evidence. In the current version, we do not provide further evidence that the gut microbiome compositions of these newborn calves remain similar to those in later years. Consequently, we have removed/modified those claims from the main text.

~~studying the maturity of the gut microbiome in newborn calves is important because it may indicate a suitable time frame for reshaping the gut microbiome through targeted intervention before maturation.~~

L292: Taken together, these results suggested that the gut microbial diversity and composition of newborn calves undergo dynamic changes during the first month of life.

~~Thus, it is important to determine at what time the gut microbiome of newborn calves reaches maturity. Here, with a longitudinal study design, our data showed that the gut microbiome of newborn calves likely reaches maturity one month after birth, as indicated by the identical microbial diversity, composition and interactions between Day 35 and Day 56 after birth.~~

Manuscript Click here to access/download;Manuscript;maintext_edLC.docx

Early microbial intervention reshapes phenotypes of newborn *Bos taurus* through metabolic regulations

Yizhao Shen[1†], Yan Li[1†], Tingting Wu[2,3,4†], Quanbin Dong[3,4†], Qiufeng Deng[4], Lu Liu[4], Yanfei Guo[1], Yufeng Cao[1], Qiufeng Li[1], Jing Shi[4], Huayiyang Zou[4], Yuwen Jiao[2], Luoyang Ding[5], Jianguo Li[1,6,7], Yanxia Gao[1,6,7]*, Shixian Hu[8]*, Yifeng Wang[3,4]* & Lianmin Chen[2,4,9]*

[1] College of Animal Science and Technology, Hebei Agricultural University, Baoding, China

[2] Changzhou Medical Center, The Affiliated Changzhou No.2 People's Hospital of Nanjing Medical University, Nanjing Medical University, Changzhou, China

[3] Cardiovascular Research Center, The Affiliated Suzhou Hospital of Nanjing Medical University, Suzhou Municipal Hospital, Gusu School, Nanjing Medical University, Suzhou, China

[4] Department of Cardiology, Nanjing Medical University, The First Affiliated Hospital of Nanjing Medical University, Nanjing, China

[5] College of Animal Science and Technology, Yangzhou University, Yangzhou, China

[6] Hebei Technology Innovation Center of Cattle and Sheep Embryo, Baoding, China

[7] Hebei Research Institute of Dairy Industry Technology, Shijiazhuang, China

[8] Institute of Precision Medicine, The First Affiliated Hospital, Sun Yat-sen University, Guangzhou, Guangdong, China

[9] Lead Contact

[†]These authors contributed equally

*Correspondence: yxgaohebau@126.com, dhu.sxhu@hotmail.com, yifengwang@njmu.edu.cn & lianminchen@njmu.edu.cn

ORCID iDs:

Yizhao Shen [0000-0003-1678-7808]; Yan Li [0000-0003-3687-9048]; Tingting Wu; Quanbin Dong [0000-0002-0849-8136]; Qiufeng Deng; Lu Liu; Yanfei Guo; Yufeng Cao; Qiufeng Li; Jing Shi; Huayiyang Zou; Yuwen Jiao; Luoyang Ding; Jianguo Li [0000-0001-7090-1574]; Yanxia Gao [0000-0002-9436-693X]; Shixian Hu; Yifeng Wang; Lianmin Chen [0000-0003-0660-3518];

ABSTRACT

Background

The rumen of neonatal calves has limited functionality and establishing intestinal microbiota may play a crucial role in their health and performance. We aim to explore the temporal colonization of the gut microbiome and the benefits of early microbial intervention in newborn calves. We evaluated the impact of microbiota transplantation (MT) on metabolism and later milk production performance.

Results

We followed 36 newborn calves for two months and found that the composition and ecological interactions of their gut microbiomes likely reached maturity one month after birth. Temporal changes in the gut microbiome of newborn calves are widely associated with changes in their physiological statuses, such as growth and fiber digestion. Importantly, we observed that MT reshapes the gut microbiome of newborns by altering the abundance and interaction of *Bacteroides* species, as well as amino acid pathways, such as arginine biosynthesis. Two-year follow-up of those calves further showed that MT improves their later milk production. Notably, MT improves fiber digestion, antioxidant capacity of newborns while reducing diarrhea. MT also contributes to significant changes in the metabolomic landscape, and with putative causal mediation analysis, we suggest that altered gut microbial composition in newborns may influence physiological status through microbial-derived metabolites.

Conclusions

Our study provides metagenomic and metabolomic atlas of the temporal development of the gut microbiome in newborn calves. MT can alter the gut microbiome of newborns, leading to improved physiological status and later milk production. The data may help develop strategies to manipulate the gut microbiota during early life, which may be relevant to the health and production of newborn calves.

Key words: gut microbiome, metagenomics, metabolomics, neonatal calf

Data Description

We followed up on the temporal dynamics of the gut microbiome (104 samples from 3 time points) and plasma metabolome (140 samples from 4 time points) in 36 newborn calves during the first two months of life, establishing their relationships with health status and growth/production performance. The metagenomic sequencing data used for the analysis presented in this study is available from the European Nucleotide Archive (ENA) under accession id PRJEB42631. The metabolic profiles are available from the Metabolomics Workbench under accession id PR001871.

Introduction

The colonization and development of the gut microbiota of newborn calves are crucial for the health and performance of the calves later in life [1]. This phenomenon is mainly attributed to the fact that these resident microbes support many functions, including the maturation of the immune system [2, 3], the utilization of nutrients [4], and the prevention of pathogen colonization [5]. Therefore, elucidating the developmental dynamics of gut microbial taxonomy and functionality during early life is important for understanding the relationships between the microbiome and host status and for eventual designing intervention strategies to achieve higher production rates and better health at later stages.

Recent studies have assessed temporal changes in the microbial taxonomic composition of newborn calves during the first week of life [6-8]. For instance, a significant increase in the relative abundance of *Lactobacillus reuteri* was observed during the first week after birth [6]. This fact seems to be very important for calf intestinal health because *L. reuteri* is known to exert bactericidal effects against bacterial pathogens and anti-infective effects against rotaviruses and *Cryptosporidium parvum in vitro* [6]. In addition, comparison of the gut microbial composition between calves (8 weeks after birth) and lactating cows showed that *Bacteroidetes* and *Verrumicrobia* were more abundant in calves, while *Firmicutes*, *Spirochaetes*, *Deinococcusthermus*, *Lentisphaerae*, *Planctomycetes*, and *Chlorofexi* were more abundant in cows [9]. These observations laid the foundation for targeted mechanistic investigations of the consequences of microbiome colonization for calf health and production.

Nevertheless, several important topics related to the temporal development of the gut microbiome in newborn calves remain unexplored. First, in addition to taxonomy, the functional composition of the gut microbiota can also undergo dynamic changes over time. Microbial functional changes, such as changes in short-chain fatty acid and amino acid metabolic pathways, due to both internal and external disruptions are implicated in the development of immunity and other systems [10, 11]. However, investigations on the temporal dynamics of gut microbial functionalities in newborn calves are still lacking. Second, the gut microbiome is an ecosystem in which microbes can compete for or exchange nutrients, signaling

molecules, or immune evasion mechanisms through complicated ecological interactions that are far from fully understood [12-14]. These interactions can be identified by co-abundance network analysis and have been shown to be related to human diseases, including obesity and inflammatory bowel diseases [15]. Investigating temporal changes in microbial interactions during the early life of newborns can enhance our understanding of gut microbial development from an ecological perspective. Third, a favorable microbiome may promote nutrient utilization and immune responses, but it is not clear whether microbial intervention during early life could improve the digestion, health status of newborn calves, as well as their later milk production performance.

To answer the above questions, we conducted the track dairy cattle study (trackDC) in which 36 newborn calves were randomly assigned into three groups: a control group, a rumen microbiota transplantation group (RMT) and an autoclaved rumen fluid transplantation group (RFT). All the newborn calves from the three groups were followed for 2 months after birth, and intensive phenotype (growth, digestion and fermentation), blood indicator, plasma metabolome and stool metagenomic analyses were conducted (**Figure S1**). In addition, the milk production performance of the calves was recorded during the two-year follow-up period. We not only investigated the temporal development of the gut microbiome at a metagenomic resolution but also evaluated whether MT could influence the phenotypes of newborn calves, including their milk production performance later in life.

Methods

Animals

The Track Dairy Cattle (trackDC) study is a longitudinal study in northern China that aims to track newborn calves to assess the development of gut microbiota during early life that contributes to cattle health and production. The study was approved by the institutional ethics review board of Hebei Agricultural University (ref. YS19003). In this study, 36 newborn calves were randomly assigned to three groups and followed for two months after birth. The groups included a control group (CON), a rumen microbiota transplantation group (RMT), and a rumen fluid transplantation group (RFT) and intensive data has been collected (**Figure S1**). The newborn calves were trained to feed milk using a bucket and then transferred to individual calf hutches. Starter was provided ad libitum three days after birth and once daily in the morning thereafter. Pasteurized whole milk was fed twice daily at 0800 and 1800 h using a bucket, and the calves were weaned 56 days after birth. RMT and RFT were performed by veterinarians, where the ruminal fluid used in RMT and RFT was collected from a healthy cattle (4-year-old, 600kg, in the dry period,) with a permanent rumen cannula 2 hours after the morning feed. Fresh ruminal fluid was mixed with raw milk and fed to the calves in the RMT group immediately after collection. For the RFT

group, the ruminal fluid was autoclaved before feeding. A volume of 50 mL, 80 mL, and 110 mL of ruminal fluid was fed from day 7 to day 11, day 21 to day 25, and day 42 to day 46, respectively. Fecal and blood samples were collected at 15, 35, and 56 days after birth. Besides, the milk production performance has also been recorded during the two-year follow-up.

Metagenomic data generation and preprocessing

Fecal samples from newborn calves were collected from rectum by stimulation of the anus and stored in liquid nitrogen after well mixed by calf. Aliquots were then made and stored at -80 °C until further processing after transferred to the laboratory. Fecal DNA isolation was performed using the QIAamp Fast DNA Stool Mini Kit (Qiagen, cat.51604). After DNA extraction, fecal DNA was used for library preparation and whole genome shotgun sequencing were performed on the Illumina NovaSeq-6000 platform (RRID:SCR_020150). From the raw metagenomic sequencing data, low-quality reads were discarded by the sequencing facility, and reads belonging to calf and human contaminations were removed by mapping the data to the reference genomes using Bowtie2 (RRID:SCR_016368; v.2.1.0) [16, 17]. After filtering, on average, 36.8 million (sd= 3.6 million) paired reads per sample were obtained for subsequent analysis.

Microbial taxonomies

Microbial taxonomic profiles were generated using MetaPhlAn2 (version 2.7.2) [18]. MetaPhlAn2 relies on nearly one million unique clade-specific marker genes identified from approximately 17,000 reference genomes, allowing unambiguous taxonomic assignments, accurate estimation of organismal relative abundance and species-level resolution for bacteria, archaea, eukaryotes and viruses. Microbial species present in more than 10% of the samples were included for further analyses. This yielded a list of 125 species that accounted for 99% of the original species abundance.

Microbial pathways

Microbial pathways were determined using HUMAnN2 (RRID:SCR_016280) [19]. HUMAnN2 reported the abundances of gene families from the UniProt Reference Clusters [20] (UniRef90), which were further mapped to microbial pathways from the MetaCyc metabolic pathway database [21, 22]. In total, we identified 345 pathways that were present in at least 10% of samples, retaining 100% of the original functional composition.

Microbial antibiotic resistance genes

The abundance of microbial antibiotic resistance genes in metagenomics was determined using

shortBRED (version 0.9.5) [23], with markers generated from the CARD database of bacterial antibiotic resistance genes[24] (01/11/2018 version). In brief, ShortBRED is a platform for identifying a set of protein sequences from a target database (i.e., ResFinder), clustering them into families, building consensus sequences to represent the families, and then reducing these consensus sequences to a set of unique identifying strings (markers). The platform then searches for these markers in metagenomic data and determines the presence and abundance of the protein families of interest. We classified the abundance of 148 antibiotic resistance genes that were present in at least 10% of the samples.

Microbial virulence genes

The abundance of microbial virulence genes was detected using shortBRED (version 0.9.5) [23] and markers generated from virulence factors of the pathogenic bacteria database (VFDB, core dataset of DNA sequences, version: November 2018) [25]. Then, we classified the abundance of 55 virulence genes that are present in at least 10% of the samples.

Growth

The initial body weight was measured immediately after birth, and the final body weight was measured on Day 56 after birth before morning feeding. Body size, including withers height, body length, heart girth, abdominal circumference, and shank circumference, was measured on Day 0 and Day 56 after birth. The starter offered to and refused by each calf were recorded daily during the experimental period. The starter offered was collected by week and refusal was collected daily, pooled by calf weekly. Both offered and refused starter samples were oven-dried at 55 °C for 48 h weekly to determine the dry matter (DM) content. The daily starter DM intake was calculated as the difference between daily starter DM offered and starter DM refused.

Digestibility and fecal score

Feed digestibility was determined using acid detergent insoluble ash as an internal marker [26]. Briefly, fecal, starter and milk samples were collected from Day 13 to Day 15, Day 33 to Day 35 d and Day 54 to Day 56, and then, the samples from each calf were pooled, dried at 55 °C for 48 h, and then ground through a 1-mm screen for further analyses. The contents of dry matter (DM, method 930.15) and crude protein (CP, method 996.11) in the starter, milk and fecal samples were determined according to AOAC International. The contents of neutral detergent fiber (NDF) and acid detergent fiber (ADF) in the starter and feces were measured using heat stable α-amylase and sodium sulfite as described by Van Soest et al. [27]. The apparent total tract digestibility was estimated as described by Rice et al. [28]. The fecal score was monitored and recorded once daily after morning feed on every calf, using a 4-level scoring system,

as described by Larson et al. [29].

Blood biomarkers

Plasma samples were used to analyze the concentrations of blood urea nitrogen (BUN), glucose, total cholesterol and triglycerides, and serum samples were used to analyze the concentrations of total protein, albumin, alkaline phosphatase, aspartate aminotransferase (AST), alanine aminotransferase (ALT), total antioxidant capacity and malonaldehyde. All the blood biomarkers were analyzed using commercial kits from Nanjing Jiancheng Bioengineering Institute (Nanjing, China). The interassay coefficients of variation were lower than 10%, and the intra-assay coefficients of variation were lower than 12%.

Ruminal volatile fatty acids and ammonia

Ruminal fluid was collected at Day 56 using an oral stomach tube before morning feeding [30]. Ruminal pH was measured immediately after collection using a pH mater (Starter 300, Ohaus Instruments Co. Ltd., Shanghai, China). Two subsamples of 5 mL were transferred into 10 mL screw-lid centrifuge tubes after filtering through 4-layer cheesecloth. One subsample was mixed with 1 mL of 25% (wt/vol) HPO_3 for volatile fatty acid (VFA) analysis, and another subsample was mixed with 1% (wt/vol) H_2SO_4 for ammonia analysis. The concentration of ruminal VFAs was measured using gas chromatography (GC-14B, Shimadzu, Japan; 30 m × 0.32 mm × 0.25 mm; column temperature, 110 °C; injector temperature, 180 °C; and detector temperature, 180 °C) [31]. The concentration of ruminal ammonia was determined as described by Rhine et al. [32].

Un-targeted plasma metabolome

Plasma samples resuspended with prechilled 80% methanol. Then the samples were incubated on ice for 5 min and centrifuged at 15,000 g, 4°C for 20 min. The supernatant was injected into the LC-MS/MS system (a ThermoFisher Vanquish UHPLC system coupled with an Orbitrap Q ExactiveTMHF mass spectrometer). The raw data files generated by UHPLC-MS/MS were processed using the Compound Discoverer 3.1 (CD3.1, ThermoFisher) to perform peak alignment, peak picking, and quantitation for each metabolite. The normalized data was used to predict the molecular formula based on additive ions, molecular ion peaks and fragment ions. And then peaks were matched with the mzCloud，mzVaultand MassList database to obtain the accurate qualitative and relative quantitative results. The annotation of metabolites using the KEGG database, HMDB database and LIPIDMaps database.

Microbial diversity

The microbial alpha (Shannon index) and beta (Bray-Curtis dissimilarity) diversities were calculated at

the species level by using the R (v3.6.0) package *vegan*.

Microbial composition dissimilarity

To compare the differences in overall microbial species composition between and within calves in each group at different time points, dimensionality reduction was carried out by using the t-distributed stochastic neighbor embedding (t-SNE) algorithm with the R package *Rtsne*. Microbiome compositional differences between groups and time points were assessed based on one the first and second t-SNE components.

Microbial species and pathway co-abundance networks

Microbial species and pathway co-abundance networks were identified by using the SparCC algorithm [33]. In detail, species composition data from MetaPhlan2 were converted to predicted read counts by multiplying relative abundances by the total sequence counts [15] and then subjected to SparCC. For pathway analysis, the read counts from HUMAnN2 cells were directly used for SparCC. Significant co-abundance was controlled at the $P< 0.01$ level using 100 times resampling.

Heterogeneity of microbial co-abundances

To assess the variability of networks between groups and different time points, we conducted Cochran-Q tests to assess the heterogeneity of effect sizes and directions for each co-abundance (correlation coefficient generated by SparCC). Here, we treated each subgroup as one study and conducted Cochran's Q test using the metagen function from the package *meta* (v4.9.5) in R, which calculates the squared difference between individual study effects and the pooled effect using inverse variance weighting [34]. For each co-abundance, the P values from the Cochran-Q test were recorded, and co-abundances with significant heterogeneity were controlled at the FDR 0.05 level determined by BH correction.

Group specific microbial co-abundances

For heterogeneous co-abundances (Cochran-Q test FDR< 0.05), we further assessed whether these relationships showed group specificity, i.e., whether the effect size of co-abundance (SparCC correlation coefficient) in one group was very different from that in the other two. We adopted interquartile ranges based on the outlier detection method [35]. The interquartile range (IQR) was calculated based on the effect size of co-abundances in each group, and we assessed whether the smallest or largest effect size fell outside of Q1-0.75IQR or Q3+0.75IQR. If only one met the condition, we called this co-abundance specific and assigned it to the corresponding group.

Differential phenotypic, microbial and metabolic features

The relative abundances of both species and pathway datasets were centered log-ratio transformed, followed by inverse-rank transformation, before subsequent analysis [36]. No transformation was applied to phenotypic data. The ranked-based Kruskal test was then applied to assess 1) whether features within certain groups showed differences between different time points and 2) whether features within certain time points showed differences between different groups. The false discovery rate (FDR) was calculated by using the Benjamini-Hochberg (BH) method [37].

Microbial changes linked to phenotypic and metabolic changes

We calculated microbial and phenotypic changes between Day 15 and Day 35 and between Days 15 and 56. We further linked microbial changes to host phenotypic and metabolic changes with Spearman correlation. Association with $P< 0.01$ were considered significant.

Mediation linkage inference

For phenotypic and metabolic associations to the same microbial feature, we first checked whether the human phenotype was associated with the metabolite using Spearman correlation ($P<0.01$). Next, mediation analysis was carried out using the mediate function from the R package mediation (version 4.5.0) to infer the causal role of the microbiome in contributing to the host phenotype through metabolites.

Results

The Track Dairy Cattle study

To investigate the temporal development of the calf gut microbiome and the potential benefits of early microbial intervention in newborn calves, we collected fecal samples from 36 newborn calves in three groups: a control group, a rumen microbiota transplantation group (RMT), and an autoclaved rumen fluid transplantation group (RFT), as part of the Track Dairy Cattle study (trackDC). Twelve newborn calves were randomly assigned to each group, and we recorded intensive phenotypes, including growth, digestion, ruminal fermentation, and blood measurements at 1, 15, 35, and 56 days after birth (**Figure S1**, **Table S1**). We observed that 14 out of 17 traits did not exhibit differences between the groups on day 1, indicating a high degree of comparability in the baseline (**Table S1**). We also observed 29 temporal differences and three differences between the groups (FDR < 0.05, **Table S2**). For instance, the digestion rates of acid detergent fiber (ADF) and neutral detergent fiber (NDF) showed temporal differences and were significantly higher in the RMT group than in the other groups, indicating that RMT can promote the digestion of fiber by newborn calves (**Figure 1A-B**, **Table S2**). Moreover, we also observed potential beneficial effects of RMT, as the blood levels of total antioxidant capacity were the highest in the RMT group compared to the other groups (**Figure 1C**, **Table S2**). By comparing the incidence of diarrhea in the three groups, we found that the RMT group had significantly fewer cases of diarrhea (**Figure 1D**). Notably, the two-year follow-up showed that the RMT group had significantly higher milk production than the other groups (**Figure 1E**). Taken together, these results indicate the beneficial effects of early microbial interventions on the health and growth of newborn calves, as well as their later milk production performance.

Temporal variations in the gut microbial composition and ecological interaction of newborn calves

To describe the temporal development of the calf gut microbiome, we first evaluated the microbial composition and diversity. A rapid increase in the microbial alpha diversity was observed between 15 and 35 days in all three groups ($P_{\text{Kruskal test}} < 1.1\times10^{-2}$), but there was no significant difference between 35 and 56 days ($P_{\text{Kruskal test}} > 0.05$, **Figure 2A**). In addition, dimensionality reduction using the t-distributed stochastic neighbor embedding (t-SNE) algorithm further showed that the microbial composition at Day 15 was significantly different from that at Days 35 and 56 ($P_{\text{Kruskal test}} < 4.0\times10^{-5}$), while no difference was observed between Days 35 and 56 ($P_{\text{Kruskal test}} > 0.05$, **Figure 2B**).

For individual microbial species and pathways, we observed that the abundance of 65 out of 125 species (52.0%) and 174 out of 345 pathways (50.4%) were significantly different among the three time points (FDR < 0.05, Kruskal test, **Table S3–4**). Importantly, the temporal development of the gut microbiome

was mainly reflected in *Bacteroides* species and the amino acid and nucleotide biosynthesis pathways. In detail, 9 out of 65 differential species were from the genus *Bacteroides* (**Figure 2C**, **Table S3**), and 50 out of 174 pathways were related to amino acid and nucleotide biosynthesis (**Table S4**). In addition, temporal changes in 80 microbial antibiotic resistance genes and 34 virulence genes were observed (FDR< 0.05, **Table S5**). Interestingly, 57 out of 80 differential microbial antibiotic resistance genes were from *Escherichia coli* (**Table S5**), a widely recognized pathogenic species. Notably, when comparing the mean abundances of microbial species and pathways between time points, we observed that microbial species abundances at Day 35 and Day 56 were more similar than those at Day 15, while pathway abundances remained relatively stable throughout (**Figure S2**). We observed that the abundance of 29 species increased with time (P<0.05), including 5 species from *Bacteroides*, 3 species from *Alistipes*, and 3 species from *Lachnospiraceae*. Meanwhile, the abundance of 23 species decreased with time (P<0.05), including 4 species from *Clostridium*, 3 species from *Bacteroides,* and 3 species from *Megamonas* (**Table S3**). Taken together, these results suggested that the gut microbial diversity and composition of newborn calves undergo dynamic changes during the first month of life.

In addition to the microbial composition, we further investigated whether microbial interactions, in terms of microbial species and pathway co-abundances, also exhibited differences between different time points in newborn calves. By using the SparCC algorithm [33], we established microbial co-abundance relationships in each subgroup separately and identified 2,393 unique species co-abundances and 38,964 pathway co-abundances (P_{SparCC}< 0.01, **Table S6–7**). To assess whether microbial co-abundance strengths could be different depending on the time after birth, we assessed to what extent the correlation coefficients were variable across subgroups and observed that on average, 42.3% (ranging from 40.5% to 43.3%) of the species co-abundances and 2.9% (ranging from 0.2% to 7.0%) of the pathway co-abundances showed heterogeneity between different time points (Cochran-Q test, FDR< 0.05, **Figure S3A**, **Table S6–7**). We next summarized the number of differential co-abundances between species from the same genus or from different genera (**Figure 2E**). The genus with the most heterogeneous co-abundances was *Bacteroides*, and many variable co-abundances were observed not only between different *Bacteroides* species but also between *Bacteroides* species and species from other genera such as *Alistipes* (**Figure 2E**). A similar observation was found for the pathway co-abundances, particularly for the nucleotides and amino acid biosynthesis pathways, which showed variability not only within themselves but also with respect to various pathways related to nucleotide biosynthesis (**Figure S3B**). These results indicate that the gut microbiome of newborn calves also undergoes dynamic temporal changes in species interactions after birth, while pathway interactions were relatively stable over time.

Early microbial transplantation reshapes the gut microbiome composition of newborn calves

As the gut microbial composition likely reaches maturity one month after birth, the accumulating evidence of the importance of the gut microbiota for overall newborn development indicates the need for early modification of the microbiota. Here, we carried out RMT by the oral infusion of fresh ruminal fluid collected from healthy adult cattle. To overcome the potential bias of metabolites in ruminal fluid, which may also influence the development of gut microbiota, we included a group of calves infused with sterilized ruminal fluid. By calculating the inter-calf Bray-Curtis dissimilarity based on the abundances of all the microbial species, we observed that inter-calf dissimilarities within the RMT group were always the lowest among those of all three groups at different time points ($P_{Kruskal\ test} < 1.4\times10^{-2}$, FDR< 0.05, **Figure 3A**). This observation is proof of concept that RMT can reshape the gut microbiome composition of newborn calves, as within group gut taxonomical compositions appear more identical in the RMT group (**Figure 3A**). When comparing individual microbial species and pathways, the relative abundance of 4 species and pathways showed a significant difference at FDR < 0.05 between groups (Kruskal test, **Table S3–4**). We observed two species from *Bacteroides* genus and many nucleotide, amino acid and carbohydrate biosynthesis pathways (e.g., arginine, ornithine and proline interconversion and superpathway of GDP-mannose-derived O-antigen building blocks biosynthesis pathways) were differential abundant in the RMT group (FDR < 0.1, **Figure 3B-C**). Notably, differential microbial abundances between groups were mainly observed at Day 15, and most of them were driven by RMT (**Figure 3B-C**), suggesting that RMT had pronounced effects in reshaping the gut microbial composition of newborn calves in the early days of life.

Microbial interactions show specificity in the transplantation group

For microbial co-abundances, we have also checked to what extent the correlation coefficients were variable between groups, and the numbers were 40.4% (ranging from 37.1% to 43.5%) and 0.3% (ranging from 0.1% to 0.6%) for species and pathway co-abundances, respectively (Cochran-Q test, FDR< 0.05, **Figure S4A**, **Table S6–7**). These results indicate that microbial interventions can alter the interactions that may potentially contribute to the development of host phenotypes. Interestingly, heterogeneous species co-abundances characterized by comparing different groups were widely distributed in many genera (**Figure S4B**), and these co-abundances were far more complex than those characterized by comparing temporal differences between different time points (**Figure S3A**). However, this was not the case in the pathway co-abundances (**Figure S4C**).

As many of the species and pathway co-abundances showed heterogeneity between groups at different time points, we further analyzed whether those heterogeneous co-abundance relationships were driven

by a particular group, i.e., whether the co-abundance strength in one group was very different from those in the other two groups at each time point. In general, we identified 633 and 101 unique group-specific species and pathway co-abundances, respectively (**Table S6–7**). Notably, 248 out of 633 (39.3%) species co-abundances and 42 out of 101 (41.6%) pathway co-abundances showed specificity for the RMT group (**Figure 4A-B**, **Table S6–7**), indicating that RMT can also alter the gut microbiome of newborn calves at the ecological level.

For those group specific co-abundances, we further evaluated potential hub species and pathways by calculating their hub score (**Table S8–9**). Interestingly, we observed a substantial amount of RMT-specific species co-abundances related to *Bifidobacterium* (**Figure 4A**), a common genus that colonized calves early in life [38, 39]. In the meanwhile, those species also have high hub scores (**Table S8**). For instance, *Bifidobacterium thermophilum* was one of the species with the most RMT-specific co-abundances (10 in total, **Table S6**), and with a high hub score of 0.98 (**Table S8**). *B. thermophilum* constitutes 80% of the infant microbiota and less than 10% of the human adult microbiota, and the presence of *Bifidobacterium* in the gut is often associated with health-promoting effects [40]. In addition, RMT-specific pathway co-abundances mainly involved sugar derivative degradation and quinone biosynthesis pathways (**Figure 4B**), but only hexitol degradation pathway had a high hub score of 1.00 (**Table S9**). The sugar derivative degradation pathway-related co-abundances showed specificity for the RMT group, which was reasonable as we observed that the digestion rates of NDF and ADF were relatively higher in the RMT group than in the other two groups (**Figure 1A-B**).

Microbial changes associated with plasma metabolites in newborn calves

To understand the potential mechanisms by which the gut microbiota could influence host physiology, we thought that metabolites are an important class of molecules that are involved in the host-microbe interaction. By profiling plasma levels of 736 metabolites at different time points using untargeted LC-MS (**Table S10**), we observed that the plasma metabolome shifts with time in newborn calves (**Figure S5**) and 50.3% of individual metabolites (370 in total) showed significant differences between groups in at least one time point with FDR <0.05 (Kruskal test, **Table S11**).

We then checked metabolic changes specifically in relation to changes in microbial composition between Day 15 and Day 56 and between Day 15 and Day 35. In total, we observed 17,602 associations between microbial and metabolite changes, and 5,221 of them were RMT specific (Spearman correlation, $r_{absolute} > 0.7$, $P < 0.01$, **Figure 5A**, **Table S12–13**). Notably, various metabolites that associated with the microbiome are already known to be related to the gut microbiome, including animal essential amino acids, bile acids, organic acids and others [3]. For instance, increased abundance of microbial L-

glutamate biosynthesis pathway (PWY-5505) associated with the increased levels of plasma glutathione (r_{RMT}= 0.95, P_{RMT}= 4.7×10^{-4}, **Figure 5B**), a tripeptide compound consisting of glutamate attached via its side chain to the N-terminus of cysteinyl glycine. Glutathione is an antioxidant that prevent oxidative damage through the reduction of methemoglobin and peroxides [41]. We also observed that increased abundance of microbial L-valine biosynthesis pathway (VALSYN-PWY) associated with the increased levels of plasma N-acetyl-valine (r_{RMT}= 0.79, P_{RMT}= 9.8×10^{-3}, **Figure 5C**). Valine is a branched-chain amino acid that cannot be biosynthesized by animals and plays important roles in insulin resistance and hematopoietic stem cell self-renewal [42, 43]. Taken together, those results suggested that RMT induced temporal changes of the gut microbiome in newborn calves may contribute their metabolic changes.

Microbial changes associated with the phenotypes of newborn calves

To examine the role of gut microbiota colonization in newborn calves, we explored the associations between microbial compositional changes and host phenotypes. To this end, we first calculated the microbial differences between Day 15 and Day 56 and between Day 15 and Day 35. Next, microbial changes were associated with their corresponding phenotypic changes, including changes in growth, digestion, fermentation and blood indicators (**Table S1**). In general, we observed 381 significant associations that involved 79 species, 185 pathways and 16 phenotypes (Spearman correlation, $r_{absolute}$> 0.7, P< 0.01, **Table S14–15**). Interestingly, we observed that 66.7% and 50.0% differential species and pathways between groups also had at least one significant association to temporal changes in phenotypes (**Table S14–15**). Such species mainly including *Lactobacillus* (*Lactobacillus amylovorus* and *Lactobacillus reuteri*) and *Alistipes* species (*Alistipes finegoldii* and *Alistipes senegalensis*), while pathways mainly including guanosine and adenosine nucleotides, as well as L-ascorbate biosynthesis. Most of the microbial associations to host phenotypes were related to changes in serum cholesterol, total protein, globulin and albumin levels, suggesting the importance of gut microbial development to newborn calf lipid metabolism and immunity. For instance, changes in the abundance of the microbial saturated fatty acid elongation pathway were associated with changes in serum cholesterol levels ($r_{Spearman}$= -0.85, P= 1.6×10^{-3}, **Table S14**). In addition, we observed that changes in serum globulin were associated with 4 prokaryotic ubiquinol biosynthesis pathways ($r_{Spearman}$> 0.79, P< 6.1×10^{-3}, **Table S15**). Ubiquinols can promote coenzyme activities to enhance lipophilic antioxidants and thus simulate host immunity [44].

Notably, 77 out of 381 associations were RMT specific (i.e., only showed significance in the RMT group or change in the opposite direction when compared with other groups, $r_{absolute}$> 0.7, P< 0.01, **Table S14–15**), and 54 associations were related to microbial pathways (**Figure 6A-B**). Among those associations,

many were related to serum aspartate transaminase (AST), malonaldehyde, total antioxidation capacity, and digestion of fiber (ADF and NDF). This suggests that RMT may potentially influence liver health, energy homeostasis, antioxidation and digestion, as reflected by changes the traits listed above (**Table S3–4**). For example, we observed that an increased abundance of microbial arginine biosynthesis was also associated with serum antioxidation capacity ($r_{Spearman}$= 0.80, P= 1.7×10^{-3}, **Figure 6C**). Arginine can effectively reduce oxidative stress through the arginine/nitric oxide pathways [45]. We also observed that an increased abundance of microbial butyrate biosynthesis was associated with serum malonaldehyde levels ($r_{Spearman}$= 0.79, P= 6.1×10^{-3}, **Figure 6D**). Supplementation with butyrate induced a marked shift in superoxide dismutase and catalase activities, along with a decrease in malonaldehyde levels, thereby attenuating oxidative stress [46]. These results suggest that RMT may potentially promote phenotypes of newborn calves by modulating microbial functionalities.

RMT alters phenotypic changes of newborn calves through metabolites

Since microbial changes can be linked to changes in both phenotypes and metabolites in newborn calves, we hypothesized that microbial impacts on host phenotypes may mediate by metabolites. To evaluate whether metabolites can mediate the microbial impact on host phenotypes, we applied mediation analysis focusing on 46 microbial features that are associated with both phenotypic and metabolic changes, which revealed 40 mediation linkages ($P_{mediation} < 0.05$, **Figure 7A-B**, **Table S16–17**). Those linkages were related to microbial impact on various phenotypes including fiber digestion, antioxidant capacity, lipid and glucose metabolism via a variable category of metabolites (**Figure 7A-B**). For example, we showed that the microbial heme biosynthesis pathway may contribute to an increase in NDF digestion by increasing plasma proline-hydroxyproline levels ($P_{mediation} = 0.02$, **Figure 7C**). Proline-rich proteins were known as negative regulator that participates in modulating fiber [47]. For antioxidant capacity, we showed that the microbial purine degradation pathway may contribute to an increase in antioxidant capacity by increasing plasma thymidine levels ($P_{mediation} = 0.02$, **Figure 7D**). Thymidine catabolism may promote NADPH oxidase-derived reactive oxygen species to induce oxidative stress [48]. We also found that a Proteobacteria species *Parasutterella excrementihominis* may contribute to the decrease in plasma triglyceride by increasing plasma androsterone levels ($P_{mediation} = 0.02$, **Figure 7E**). Androsterone is an effective lipid-lowing agent [49]. In summary, these results suggest that microbial changes induced by RMT may reshape phenotypes of newborn calves through modulating host metabolism.

Discussion

A diverse microbial population colonizes the mammalian gastrointestinal tract during/after birth, and increasing evidence now suggests that this complex microbiome plays a crucial role in the development

of the mucosal immune system and influences newborn health [1, 50]. Recent studies have tracked the temporal changes of the gastrointestinal microbiota in newborn calves during the first several weeks after birth at a 16S rRNA resolution, which is limited on taxonomic composition [6-8, 51]. However, the key to understanding the importance of microbial development to the host is to investigate whether within-calf microbial differences can be associated with changes in various phenotypes. We therefore systematically characterized the microbial changes at both the taxonomic and functional levels by using fecal metagenomic sequencing data from 36 newborn calves in the trackDC study.

Previous investigations on the temporal development (within 2 months) of the microbial composition of newborn calves with 16S rRNA sequencing have revealed a list of microbial genera that exhibit significant temporal differences, including *Enterococcus*, *Lactobacillus*, *Escherichia*, *Bifidobacterium*, *Clostridium*, etc. [6-8, 51]. Our in-depth metagenomic sequencing of the gut microbiome extends this observation at the species resolution, which enhances the current understanding. In addition, characterization of the temporal changes in microbial pathway abundances further showed that microbial functionalities, such as amino acid, organic acid and carbohydrate metabolism, undergo dynamic changes in newborn calves.

This result was mainly attributed to the fact that microbe colonization of the gastrointestinal tract and their functionalities contribute to changing the ruminant digestive system from a monogastric system to a fully functional foregut rumen fermenter system, with an ability to digest fibrous feed, postweaning. The period from birth to weaning is important for rumen microbial colonization and adaptation. Once the development and maturation of the rumen and the microbiome are complete, it is difficult to permanently manipulate or change the rumen ecosystem due to microbial adaptation and resilience to external mediators [52]. In early life, a favorable microbiome can be implanted via dietary or management interventions and have potentially a long-lasting effect [1, 53, 54].

Here, we showed that the gut microbiome of newborn calves can be altered by RMT at both taxonomic and functional levels, as indicated by species and pathway abundances, respectively. For instance, we observed a higher abundance of the beneficial species *Parabacteroides distasonis* in the RMT group, and this species can alleviate metabolic dysfunctions by generating succinate and secondary bile acids, which activate the intestinal gluconeogenesis pathway and farnesoid X receptor, in the gut [55]. In addition, we showed that microbial interactions in terms of co-abundances showed heterogeneity between groups and characterized many RMT-specific species and pathway co-abundances. The diverse microbial communities in the gut make up a complicated ecosystem in which microbes can exchange or compete for nutrients, signaling molecules, or immune evasion mechanisms through ecological interactions that

are far from fully understood [12, 13]. Our analyses show that microbial alterations by RMT may not be driven solely by differences in abundance level; it may also reflect shifts in microbial interactions that are mirrored in co-abundance analyses. Particularly when applied to metagenomics sequence data, pathway-based co-abundance networks provide further insights into the functional alterations caused by RMT, as many RMT-specific pathway co-abundances have been identified.

Characterization of the temporal changes in the gut microbiome is crucial for understanding the role of the gut microbiome in phenotypic development of newborn calves. By linking microbial changes to phenotypic changes in newborn calves in different groups, we observed thousands of associations between the microbiome, phenotypes and metabolism, including digestion, lipid metabolism, and immunity. Interestingly, many of those associations were only present in the RMT group. For example, RMT may increase arginine production, which further enhances the antioxidation capacity. Another example is that increased butyrate production in response to the increased amount of malonaldehyde in the RMT group. With mediation analysis by linking microbial, phenotypic and metabolic changes, our analysis suggests that microbial changes induced by RMT may reshape phenotypes of newborn calves through modulating host metabolism. Thus, our longitudinal analysis of microbial association with calf phenotypes and blood indicators revealed functional insights and putative causality of the role of the gut microbiome in newborn calf health status. These observations are of great importance for guiding further studies to develop strategies that may be used to manipulate the early microbiome to improve production and health during the time when newborn calves are most susceptible to enteric disease.

We acknowledge several limitations in the present study. Firstly, the trackDC study sampled fecal and blood samples from 36 newborn calves during the first two months after birth, making it the first metagenomics and metabolomics-based longitudinal study with the largest sample size to date. However, despite our random assignment of calves to various groups, the absence of baseline fecal microbiome profiles could potentially introduce bias to the observed differences. And the conducted analysis, such as mediation analysis may under power. Therefore, replicating these findings in independent studies with larger sample sizes like human cohort studies [56] could significantly enhance the robustness of our observations and emphasize their biological significance. Secondly, we employed various intervention volumes with the intention of mitigating potential bias arising from the natural growth of the gastrointestinal tract in newborn calves over time. However, it's important to note that this approach could introduce new biases, as we did not directly measure the actual size of their gastrointestinal tracts. Thirdly, the reported results are association-based, which means that the underlying causalities and mechanisms of action remain unexplored. Functional studies are thus essential to further reveal the

underlying mechanisms of the reported associations. Finally, we primarily focused on the gut microbiome of newborn calves. However, given the fact that the temporal development of their rumen microbiome is also important but not easily sampled, further studies linking the gut and rumen microbiomes in newborn calves may provide a more systematic understanding of the importance of the microbiome in newborn calves.

Availability of source code and requirements

- Project name: The trackDC study
- Project home page: https://github.com/MicrobiomeCardioMetaLab/trackDC.abundance_project
- Operating system(s): Platform independent
- Programming language: R
- Other requirements: none.
- Any restrictions to use by non-academics: licence GPL-3
- RRID: SCR_020150; SCR_016368; SCR_016280.

Data availability

The metagenomic sequencing data used for the analysis presented in this study are available from the European Nucleotide Archive (ENA) under accession id PRJEB42631. The metabolic profiles are available from the Metabolomics Workbench under accession id PR001871. All supporting data and materials are available in the *GigaScience* GigaDB database [57].

List of abbreviations

MT, microbiota transplantation; trackDC, the track dairy cattle study; RMT, rumen microbiota transplantation group; RFT, autoclaved rumen fluid transplantation group; CON, control group; DM, dry matter; CP, crude protein; NDF, neutral detergent fiber; ADF, acid detergent fiber; BUN, blood urea nitrogen; AST, aspartate aminotransferase; ALT, alanine aminotransferase; VAF, volatile fatty acid; FDR, false discovery rate.

Acknowledgements

We thank the management staff of the trackDC for their supports and the High-Performance Computing Centre of Nanjing Medical University for providing computational resources. This project was funded by the National Natural Science Foundation of China (NSFC, 32302756, 32394052 & 32270077); The Earmarked Fund (CARS36); Hebei Dairy Cattle Innovation Team of Modern Agro-industry Technology Research System (HBCT2023180207); The Top Talent Project of Hebei Province (6012018); Precision Animal Husbandry Discipline Group Construction Project of Hebei Agricultural University (1090064); Natural Science Foundation of Hebei (C2022204248 & C2022204247); Natural Science Foundation of Jiangsu (BK20220709); Jiangsu Shuangchuang Project (Medical Expert & JSSCBS20221815); Nanjing Medical University, Changzhou Medical Centre Grant (CMCM202204); Nanjing Medical University (303073572NC21 & YNRCZN0301); Development of Jiangsu Higher Education Institutions Priority Academic Program (PAPD). The funders had no role in the study design, data collection and analysis, decision to publish, or preparation of the manuscript.

Author contributions

Y.S. and L.C. conceptualized and managed the study. Y.S., Y.L., Q.D, L.L., Y.G., Y.C., Q.L., J.S., H.Z., Y.G., L.D., J.L., Y.G., S.H., Y.W., and L.C. collected the samples and generated the data. Y.S. and L.C. analysed the data. Y.S. and L.C. drafted the manuscript. Y.S., Y.L., T.W., Q.D., Q.D, L.L., Y.G., Y.C., Q.L., J.S., H.Z., Y.G., L.D., J.L., Y.G., S.H., Y.W., and L.C. reviewed and edited the manuscript.

Competing Interests

The authors declare no competing interests.

Figure Legends

Figure 1. Temporal and microbial transplantation induced variations of phenotypes in newborn calves. A. Temporal changes of neutral detergent fiber (NDF) digestibility. P values from Kruskal test are shown accordingly. **B.** Temporal changes of acid detergent fiber (ADF) digestibility. P values from Kruskal test are shown accordingly. **C.** Temporal changes of plasma total antioxidant capacity. P values from Kruskal test are shown accordingly. **D.** Occurrence of diarrhea between groups in the whole experiential period (56 days). P values from Fisher exact test are shown accordingly. **E.** Differences in the later milk production of those calves during the two-year follow-up. P values from Wilcoxon test are shown accordingly.

Figure 2. Temporal variations of the gut microbiome in newborn calves. A. Temporal changes of the Shannon index based on species level abundance. P values from Kruskal test are shown accordingly. **B.** Temporal changes of the gut microbial composition on species level abundance. P values from Kruskal test are shown accordingly. **C.** Number of species showed temporal differences within different genus. **D.** Summary of differential microbial species co-abundances between time points at genus level. Each line represents differential species co-abundances between species from either the same or different genera. The width and darkness of the lines represent the relative number of differential co-abundances.

Figure 3. Microbiota transplantation reshapes the gut microbiome composition of newborn calves. A. Within group microbial compositional similarity. The Bray-Curtis (BC) distance represents the dissimilarity of microbial species composition between two samples. P values from Kruskal and Dunn's tests are shown. **B.** Differential species abundance between groups. The darkness of color represents the effect size of microbial abundance when comparing with the control group. P and FDR values from Kruskal and Dunn's tests are shown. **C.** Differential pathway abundance between groups. METHGLYUT-PWY:superpathway of methylglyoxal degradation; RGORNPROST-PWY:arginine, ornithine and proline interconversion; PWY-5505:L-glutamate and L-glutamine biosynthesis; PWY-6628:superpathway of L-phenylalanine biosynthesis; P23-PWY:reductive TCA cycle I; COLANSYN-PWY:colanic acid building blocks biosynthesis; PWY-7323:superpathway of GDP-mannose-derived O-antigen building blocks biosynthesis; PWY-2723:trehalose degradation V; PWY-4041:gamma;-glutamyl cycle; PWY-6588:pyruvate fermentation to acetone; PWY-6125:superpathway of guanosine nucleotides de novo biosynthesis II; PWY-6126:superpathway of adenosine nucleotides de novo biosynthesis II; PWY-7220:adenosine deoxyribonucleotides de novo biosynthesis II; PWY-7222:guanosine deoxyribonucleotides de novo biosynthesis II; PWY0-1479:tRNA processing; PWY-241:C4 photosynthetic carbon assimilation cycle, NADP-ME type; PWY-7115:C4 photosynthetic carbon

assimilation cycle, NAD-ME type; PWY-7117:C4 photosynthetic carbon assimilation cycle, PEPCK type; PWY-7446:sulfoquinovose degradation I; PWY-7254:TCA cycle VII (acetate-producers); PWY-6415:L-ascorbate biosynthesis V. The darkness of color represents the effect size of microbial abundance when comparing with the control group. P and FDR values from Kruskal and Dunn's tests are shown.

Figure 4. Microbiota transplantation specific species and pathway co-abundances. A. 248 microbial intervention specific species co-abundances. Each dot indicates one species while each line represents a microbial intervention specific correlation between two species. Species in red are hub nodes with hub score larger than 0.5. **B.** 42 microbial intervention specific pathway co-abundances. Each dot indicates one pathway while each line represents a microbial intervention specific correlation between two pathways. Pathway in red is the hub node with hub score larger than 0.5.

Figure 5. Microbial compositional changes associated with plasma metabolic changes. A. Overview of 5,221 microbial intervention-specific microbe-metabolite associations between day 15 and day 35, day 15 and day 56. The associated microbial factors are colored gray, and the associated metabolites are colored by other colors. **B.** Positive association between plasma glutathione and microbial glutamate biosynthesis pathway changes in the microbial intervention group between day 15 and day 35. **C.** Positive association between plasma N-acetyl-valine and microbial valine biosynthesis pathway changes in the microbial intervention group between day 20 and day 35. Spearman correlation is applied to assess the associations between microbial and phenotypic changes.

Figure 6. Microbial compositional changes associated with phenotypic changes in newborn calves. A. Microbial changes between day 15 and day 56 associated with the corresponding phenotypic changes. **B.** Microbial changes between day 15 and day 35 associated with the corresponding phenotypic changes. **C.** Positive association between antioxidation capacity and microbial arginine biosynthesis pathway changes in the microbial intervention between day 15 and day 56. **D.** Positive association between blood malonaldehyde and microbial butyrate biosynthesis pathway changes in the microbial intervention group between day 15 and day 35.

Figure 7. Mediation linkages among the gut microbial, metabolic and phenotypic changes. A. Sankey plot showing the 11 significant mediation effects of plasma metabolites between day 15 and day 35. **B.** Sankey plot showing the 29 significant mediation effects of plasma metabolites between day 15 and day 56. The left panel shows the microbial factors, the middle panel shows the plasma metabolites, and the right panel shows the phenotypes. The curved lines across panels indicate the mediation effects,

while the colors correspond to different phenotypes. **C.** Proline-hydroxyproline mediates the effect of microbial heme biosynthesis pathway on NDF digestion. **D.** Thymidine mediates the effect of microbial purine degradation pathway on total antioxidant capacity. **E.** Androsterone mediates the effect of *Parasutterella excrementihominis* on triglyceride.

Figure S1. Study design and overview of the data that has been collected during the first two months.

Figure S2. Comparison of microbial feature abundances between groups at different time points. Spearman correlation is applied to assess the associations of microbial abundances between different time points.

Figure S3. Temporal differences in microbial co-abundance networks. A. Summary of microbial species and pathway co-abundances in each group at different time points. The number of significant co-abundances has been listed and the heterogeneity of co-abundances is assessed by Cochran-Q test. **B.** Summary of differential microbial pathway co-abundances between time points at metabolic category level. Each line represents differential pathway co-abundances between pathways from either the same or different metabolic categories. The width and darkness of the lines represent the relative number of differential co-abundances.

Figure S4. Differential microbial co-abundance networks between groups. A. Summary of microbial species and pathway co-abundances in each group at different time points. The number of significant co-abundances has been listed and the heterogeneity of co-abundances is assessed by Cochran-Q test. **B.** Summary of differential microbial species co-abundances between groups at genus level. Each line represents differential species co-abundances between species from either the same or different genera. The width and darkness of the lines represent the relative number of differential co-abundances. **C.** Summary of differential microbial pathway co-abundances between groups. Each line represents differential pathway co-abundances between pathways from either the same or different metabolic categories. The width and darkness of the lines represent the relative number of differential co-abundances.

Figure S5. The composition of plasma metabolites shows temporal and group differences. P values from Kruskal test are shown accordingly.

Table Legends

Table S1. Summary of phenotypes collected in this study

Table S2. Phenotypic differences between groups and time points

Table S3. Differential microbial species abundances between groups and time points

Table S4. Differential microbial pathway abundances between groups and time points

Table S5. Differential microbial antibiotic resistance and virulence gene abundances between groups and time points

Table S6. Microbial species co-abundances

Table S7. Microbial pathway co-abundances

Table S8. Hub scores of species in co-abundance networks

Table S9. Hub scores of pathways in co-abundance networks

Table S10. Summary of plasma metabolome

Table S11. Differential plasma metabolites between groups

Table S12. Microbial changes between day15 and day35 associated with metabolic changes

Table S13. Microbial changes between day15 and day56 associated with metabolic changes

Table S14. Microbial changes between day15 and day35 associated with phenotypic changes

Table S15. Microbial changes between day15 and day56 associated with phenotypic changes

Table S16. Metabolites mediate microbial impacts on phenotypic changes between day15 and day35

Table S17. Metabolites mediate microbial impacts on phenotypic changes between day15 and day56

References

1. Arshad MA, Hassan F-u, Rehman MS, Huws SA, Cheng Y, Din AU: **Gut microbiome colonization and development in neonatal ruminants: Strategies, prospects, and opportunities.** *Animal Nutrition* 2021. 7(3), 883-895.
2. Thaiss CA, Zmora N, Levy M, Elinav E: **The microbiome and innate immunity.** *Nature* 2016, **535:**65-74.
3. Wang Y, Dong Q, Hu S, Zou H, Wu T, Shi J, Zhang H, Sheng Y, Sun W, Kong X: **Decoding microbial genomes to understand their functional roles in human complex diseases.** *iMeta* 2022**:**e14.
4. Nicholson JK, Holmes E, Kinross J, Burcelin R, Gibson G, Jia W, Pettersson S: **Host-gut microbiota metabolic interactions.** *Science* 2012, **336:**1262-1267.
5. Buffie CG, Pamer EG: **Microbiota-mediated colonization resistance against intestinal pathogens.** *Nat Rev Immunol* 2013, **13:**790-801.
6. Schwaiger K, Storch J, Bauer C, Bauer J: **Development of selected bacterial groups of the rectal microbiota of healthy calves during the first week postpartum.** *J Appl Microbiol* 2020, **128:**366-375.
7. Takino T, Kato-Mori Y, Motooka D, Nakamura S, Iida T, Hagiwara K: **Postnatal changes in the relative abundance of intestinal Lactobacillus spp. in newborn calves.** *J Vet Med Sci* 2017, **79:**452-455.
8. Song Y, Malmuthuge N, Steele MA, Guan LL: **Shift of hindgut microbiota and microbial short chain fatty acids profiles in dairy calves from birth to pre-weaning.** *FEMS Microbiol Ecol* 2018, **94**(3):fix179.
9. Haley BJ, Kim SW, Salaheen S, Hovingh E, Van Kessel JAS: **Differences in the Microbial Community and Resistome Structures of Feces from Preweaned Calves and Lactating Dairy Cows in Commercial Dairy Herds.** *Foodborne Pathog Dis* 2020, **17:**494-503.
10. Yang W, Yu T, Huang X, Bilotta AJ, Xu L, Lu Y, Sun J, Pan F, Zhou J, Zhang W, et al: **Intestinal microbiota-derived short-chain fatty acids regulation of immune cell IL-22 production and gut immunity.** *Nat Commun* 2020, **11:**4457.
11. Visconti A, Le Roy CI, Rosa F, Rossi N, Martin TC, Mohney RP, Li W, de Rinaldis E, Bell JT, Venter JC, et al: **Interplay between the human gut microbiome and host metabolism.** *Nat Commun* 2019, **10:**4505.
12. Baumler AJ, Sperandio V: **Interactions between the microbiota and pathogenic**

bacteria in the gut. *Nature* 2016, **535:**85-93.

13. Whiteley M, Diggle SP, Greenberg EP: **Progress in and promise of bacterial quorum sensing research.** *Nature* 2017, **551:**313-320.

14. Chen L, Garmaeva S, Zhernakova A, Fu J, Wijmenga C: **A system biology perspective on environment-host-microbe interactions.** *Hum Mol Genet* 2018, **27:**R187-R194.

15. Chen L, Collij V, Jaeger M, van den Munckhof ICL, Vich Vila A, Kurilshikov A, Gacesa R, Sinha T, Oosting M, Joosten LAB, et al: **Gut microbial co-abundance networks show specificity in inflammatory bowel disease and obesity.** *Nature Communications* 2020, **11:**4018.

16. Langmead B, Trapnell C, Pop M, Salzberg SL: **Ultrafast and memory-efficient alignment of short DNA sequences to the human genome.** *Genome Biol* 2009, **10:**R25.

17. Langmead B, Wilks C, Antonescu V, Charles R: **Scaling read aligners to hundreds of threads on general-purpose processors.** *Bioinformatics* 2019, **35:**421-432.

18. Truong DT, Franzosa EA, Tickle TL, Scholz M, Weingart G, Pasolli E, Tett A, Huttenhower C, Segata N: **MetaPhlAn2 for enhanced metagenomic taxonomic profiling.** *Nat Methods* 2015, **12:**902-903.

19. Franzosa EA, McIver LJ, Rahnavard G, Thompson LR, Schirmer M, Weingart G, Lipson KS, Knight R, Caporaso JG, Segata N, Huttenhower C: **Species-level functional profiling of metagenomes and metatranscriptomes.** *Nature Methods* 2018, **15:**962-968.

20. Bateman A, Martin MJ, O'Donovan C, Magrane M, Apweiler R, Alpi E, Antunes R, Ar-Ganiska J, Bely B, Bingley M, et al: **UniProt: a hub for protein information.** *Nucleic Acids Research* 2015, **43:**D204-D212.

21. Caspi R, Billington R, Ferrer L, Foerster H, Fulcher CA, Keseler IM, Kothari A, Krummenacker M, Latendresse M, Mueller LA, et al: **The MetaCyc database of metabolic pathways and enzymes and the BioCyc collection of pathway/genome databases.** *Nucleic Acids Research* 2016, **44:**D471-D480.

22. Caspi R, Billington R, Fulcher CA, Keseler IM, Kothari A, Krummenacker M, Latendresse M, Midford PE, Ong Q, Ong WK, et al: **The MetaCyc database of metabolic pathways and enzymes.** *Nucleic Acids Research* 2018, **46:**D633-D639.

23. Kaminski J, Gibson MK, Franzosa EA, Segata N, Dantas G, Huttenhower C: **High-Specificity Targeted Functional Profiling in Microbial Communities with**

ShortBRED. *PLoS Comput Biol* 2015, **11:**e1004557.

24. Jia B, Raphenya AR, Alcock B, Waglechner N, Guo P, Tsang KK, Lago BA, Dave BM, Pereira S, Sharma AN, et al: **CARD 2017: expansion and model-centric curation of the comprehensive antibiotic resistance database.** *Nucleic Acids Res* 2017, **45:**D566-D573.
25. Liu B, Zheng D, Jin Q, Chen L, Yang J: **VFDB 2019: a comparative pathogenomic platform with an interactive web interface.** *Nucleic Acids Res* 2019, **47:**D687-D692.
26. Li Y, Shen Y, Niu J, Guo Y, Pauline M, Zhao X, Li Q, Cao Y, Bi C, Zhang X, et al: **Effect of active dry yeast on lactation performance, methane production, and ruminal fermentation patterns in early-lactating Holstein cows.** *J Dairy Sci* 2021, **104:**381-390.
27. Van Soest PJ, Robertson JB, Lewis BA: **Methods for dietary fiber, neutral detergent fiber, and nonstarch polysaccharides in relation to animal nutrition.** *J Dairy Sci* 1991, **74:**3583-3597.
28. Rice EM, Aragona KM, Moreland SC, Erickson PS: **Supplementation of sodium butyrate to postweaned heifer diets: Effects on growth performance, nutrient digestibility, and health.** *J Dairy Sci* 2019, **102:**3121-3130.
29. Larson LL, Owen FG, Albright JL, Appleman RD, Lamb RC, Muller LD: **Guidelines toward more uniformity in measuring and reporting calf experimental data.** *Journal of Dairy Science* 1977, **60:**989-991.
30. Shen JS, Chai Z, Song LJ, Liu JX, Wu YM: **Insertion depth of oral stomach tubes may affect the fermentation parameters of ruminal fluid collected in dairy cows.** *Journal of Dairy Science* 2012, **95:**5978-5984.
31. Shen YZ, Ding LY, Chen LM, Xu JH, Zhao R, Yang WZ, Wang HR, Wang MZ: **Feeding corn grain steeped in citric acid modulates rumen fermentation and inflammatory responses in dairy goats.** *Animal* 2019, **13:**301-308.
32. Rhine ED, Sims GK, Mulvaney RL, Pratt EJ: **Improving the Berthelot reaction for determining ammonium in soil extracts and water.** *Soil Science Society of America Journal* 1998, **62:**473-480.
33. Friedman J, Alm EJ: **Inferring Correlation Networks from Genomic Survey Data.** *Plos Computational Biology* 2012, **8**(9):e1002687.
34. Schwarzer G: **meta: An R package for meta-analysis.** *R News* 2007, **7:**3:40-5.
35. Barbato G, Barini EM, Genta G, Levi R: **Features and performance of some outlier**

detection methods. *Journal of Applied Statistics* 2011, **38:**2133-2149.

36. Aitchison J: **The statistical analysis of compositional data.** *Journal of the Royal Statistical Society: Series B (Methodological)* 1982, **44:**139-160.

37. Benjamini Y, Hochberg Y: **Controlling the False Discovery Rate - a Practical and Powerful Approach to Multiple Testing.** *Journal of the Royal Statistical Society Series B-Methodological* 1995, **57:**289-300.

38. Yassour M, Jason E, Hogstrom LJ, Arthur TD, Tripathi S, Siljander H, Selvenius J, Oikarinen S, Hyoty H, Virtanen SM, et al: **Strain-Level Analysis of Mother-to-Child Bacterial Transmission during the First Few Months of Life.** *Cell Host Microbe* 2018, **24:**146-154.

39. Gacesa R, Kurilshikov A, Vich Vila A, Sinha T, Klaassen MAY, Bolte LA, Andreu-Sánchez S, Chen L, Collij V, Hu S, et al: **The Dutch Microbiome Project defines factors that shape the healthy gut microbiome.** *bioRxiv* 2020**:**2020.2011.2027.401125.

40. Hussein L, Singh RB: **Developments on the Applications and the Suitability of Functional Fermented Sour Sobya as a Viable Source of Novel Probiotics in the Managements of Gastrointestinal Disorders and Blood Lipid Profiles.** In *The Role of Functional Food Security in Global Health.* Elsevier; 2019: 579-602

41. Gaucher C, Boudier A, Bonetti J, Clarot I, Leroy P, Parent M: **Glutathione: Antioxidant Properties Dedicated to Nanotechnologies.** *Antioxidants (Basel)* 2018, **7 (5):62**.

42. Cummings NE, Williams EM, Kasza I, Konon EN, Schaid MD, Schmidt BA, Poudel C, Sherman DS, Yu D, Arriola Apelo SI, et al: **Restoration of metabolic health by decreased consumption of branched-chain amino acids.** *J Physiol* 2018, **596:**623-645.

43. Taya Y, Ota Y, Wilkinson AC, Kanazawa A, Watarai H, Kasai M, Nakauchi H, Yamazaki S: **Depleting dietary valine permits nonmyeloablative mouse hematopoietic stem cell transplantation.** *Science* 2016, **354:**1152-1155.

44. Zhang Y, Liu J, Chen X-q, Chen C-YO: **Ubiquinol is superior to ubiquinone to enhance Coenzyme Q10 status in older men.** *Food & function* 2018, **9:**5653-5659.

45. Shan LL, Wang B, Gao GZ, Cao WG, Zhang YK: **L-Arginine supplementation improves antioxidant defenses through L-arginine/nitric oxide pathways in exercised rats.** *Journal of Applied Physiology* 2013, **115:**1146-1155.

46. Zhou Y, Ji XW, Chen JJ, Fu YY, Huang JW, Guo R, Zhou JH, Cen JK, Zhang QH, Chu AN, et al: **Short-chain fatty acid butyrate: A novel shield against chronic gastric ulcer.** *Experimental and Therapeutic Medicine* 2021, **21(4), pp.1-1.**.

47. Xu WL, Zhang DJ, Wu YF, Qin LX, Huang GQ, Li J, Li L, Li XB: **Cotton PRP5 gene encoding a proline-rich protein is involved in fiber development.** *Plant Mol Biol* 2013, **82:**353-365.

48. Tabata S, Yamamoto M, Goto H, Hirayama A, Ohishi M, Kuramoto T, Mitsuhashi A, Ikeda R, Haraguchi M, Kawahara K, et al: **Thymidine catabolism promotes NADPH oxidase-derived reactive oxygen species (ROS) signalling in KB and yumoto cells.** *Sci Rep* 2018, **8:**6760.

49. Oliver MF: **Reduction of serum-lipid and uric-acid levels by an orally active androsterone.** *Lancet* 1962, **1:**1321-1323.

50. Malmuthuge N, Griebel PJ, Guan le L: **The Gut Microbiome and Its Potential Role in the Development and Function of Newborn Calf Gastrointestinal Tract.** *Front Vet Sci* 2015, **2:**36.

51. Kim ET, Lee SJ, Kim TY, Lee HG, Atikur RM, Gu BH, Kim DH, Park BY, Son JK, Kim MH: **Dynamic Changes in Fecal Microbial Communities of Neonatal Dairy Calves by Aging and Diarrhea.** *Animals (Basel)* 2021, **11(4):1113.**.

52. Clemmons BA, Voy BH, Myer PR: **Altering the Gut Microbiome of Cattle: Considerations of Host-Microbiome Interactions for Persistent Microbiome Manipulation.** *Microb Ecol* 2019, **77:**523-536.

53. Palma-Hidalgo JM, Jimenez E, Popova M, Morgavi DP, Martin-Garcia AI, Yanez-Ruiz DR, Belanche A: **Inoculation with rumen fluid in early life accelerates the rumen microbial development and favours the weaning process in goats.** *Anim Microbiome* 2021, **3:**1-21.

54. Malmuthuge N, Liang GX, Guan LL: **Regulation of rumen development in neonatal ruminants through microbial metagenomes and host transcriptomes.** *Genome Biology* 2019, **20** (1):1-6.

55. Wang K, Liao M, Zhou N, Bao L, Ma K, Zheng Z, Wang Y, Liu C, Wang W, Wang J, et al: **Parabacteroides distasonis Alleviates Obesity and Metabolic Dysfunctions via Production of Succinate and Secondary Bile Acids.** *Cell Rep* 2019, **26:**222-235 e225.

56. Sun W, Wang Y, Zhang H, Sheng Y, Fan J, Gu M, Tian Y, Zhang Y, Ma H, Yin X:

eHypertension: A prospective longitudinal multi- omics essential hypertension cohort. Wiley Online Library; 2022. e22.

57. Shen Y; Li Y; Wu T; Dong Q; Deng Q; Liu L; Guo Y; Cao Y; Li Q; Shi J; Zou H; Jiao Y; Ding L; Li J; Gao Y; Hu S; Wang Y; Chen L. Supporting data for "Early microbial intervention reshapes phenotypes of newborn Bos taurus through metabolic regulations" GigaScience Database 2023. http://dx.doi.org/10.5524/102484

Figure1

Click here to access/download;Figure;Figure1.pdf

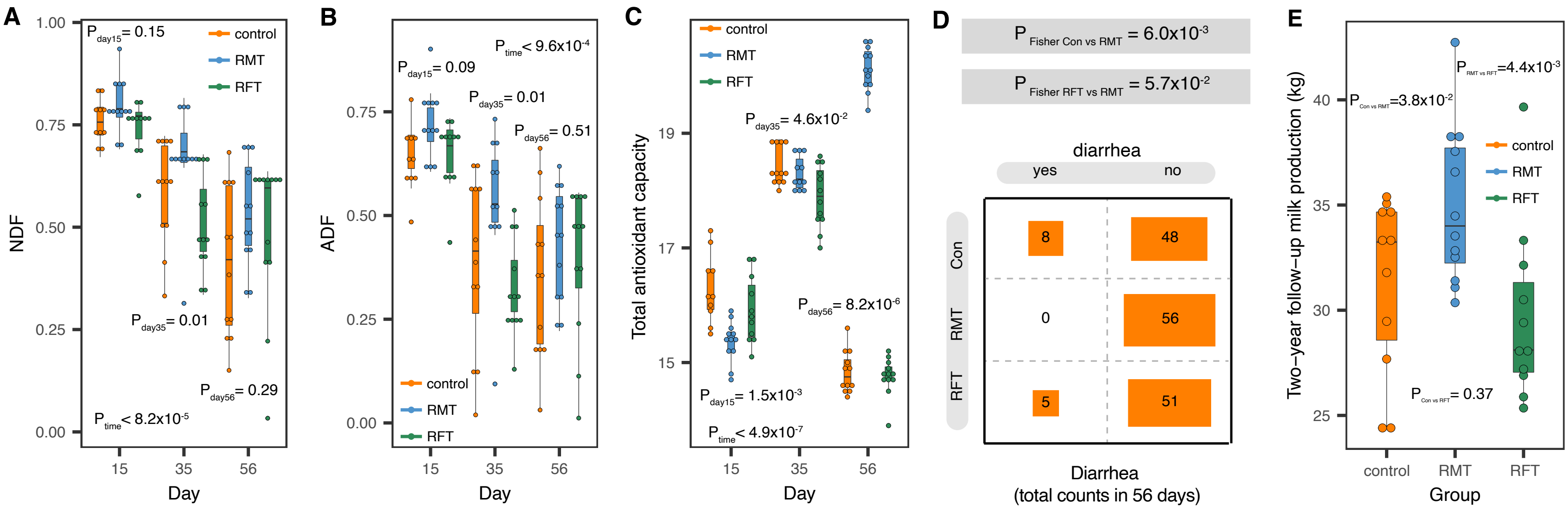

Figure2

Click here to access/download;Figure;Figure2.pdf

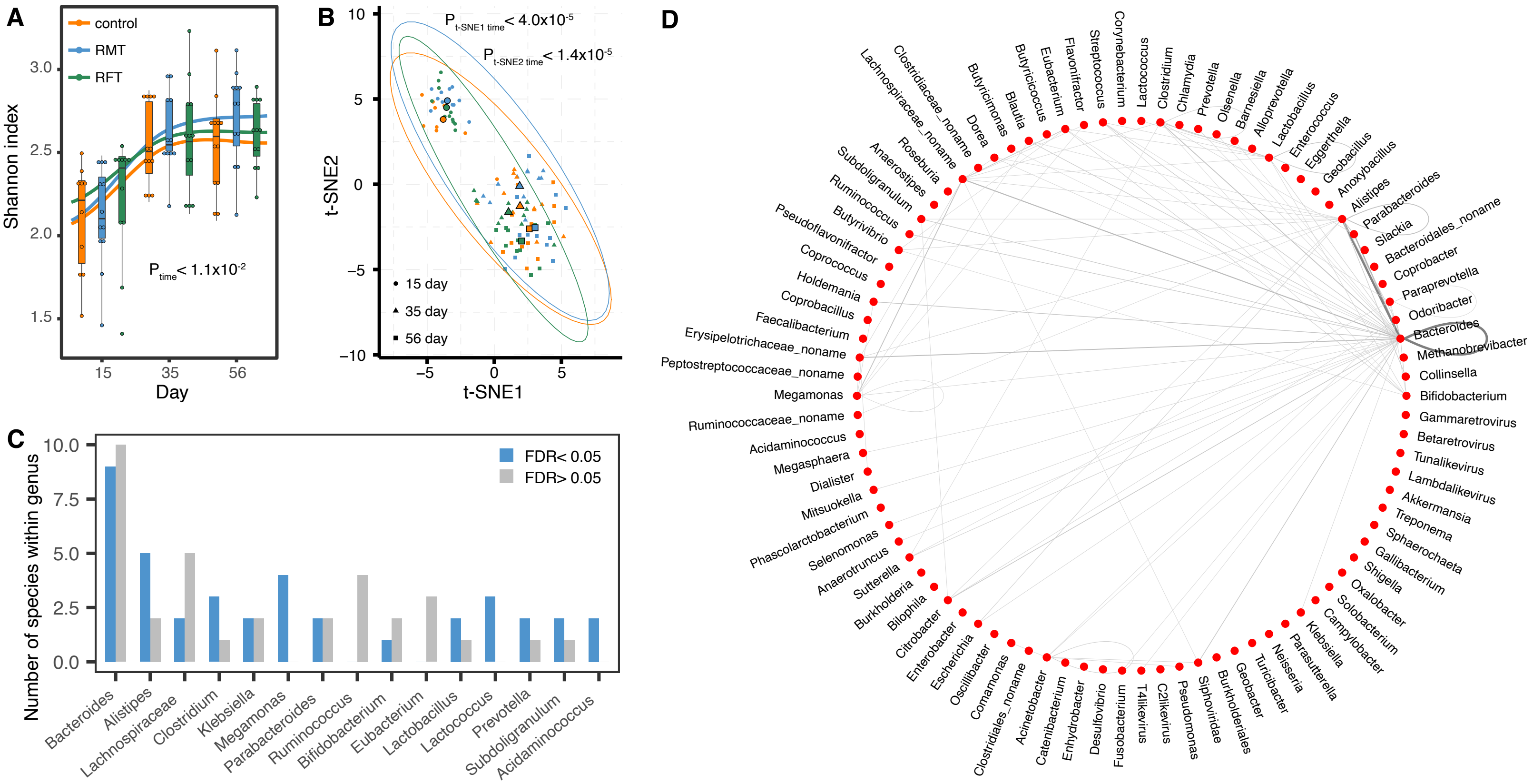

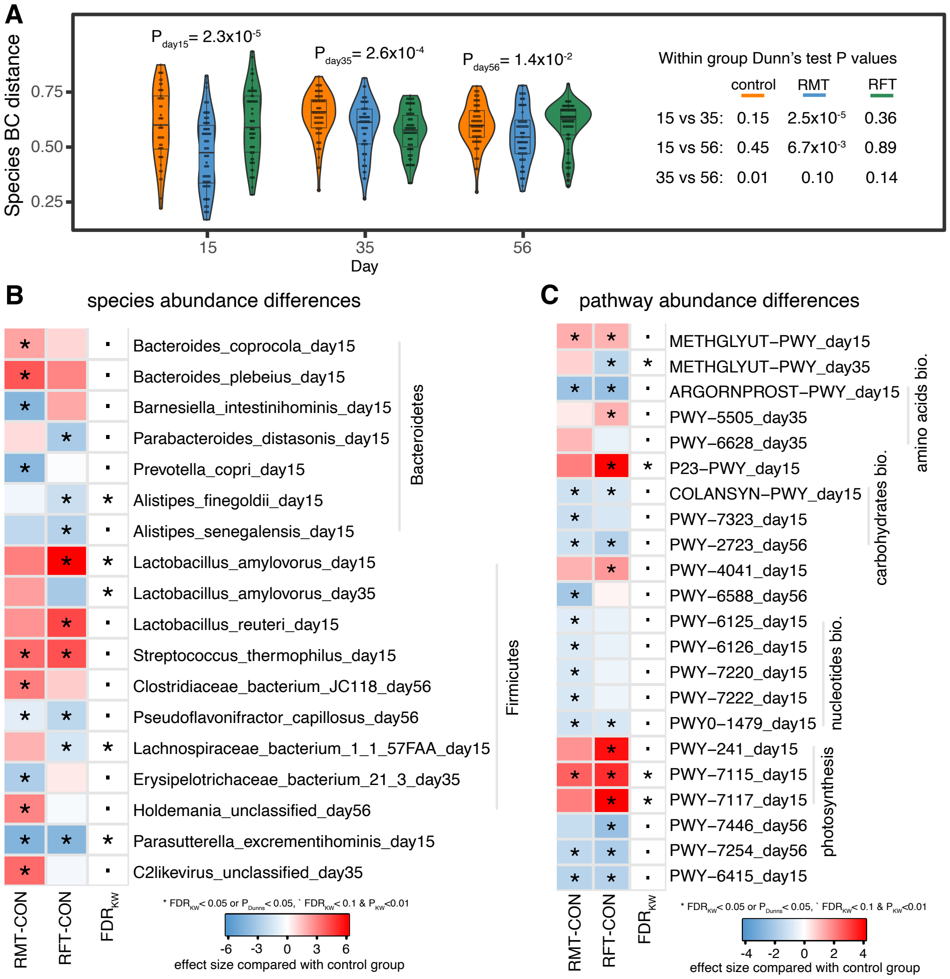
A
Species BC distance
Day
Within group Dunn's test P values
control RMT RFT
15 vs 35: 0.15 2.5x10-5 0.36
15 vs 56: 0.45 6.7x10-3 0.89
35 vs 56: 0.01 0.10 0.14
B
species abundance differences
Bacteroidetes
Firmicutes
Bacteroides_coprocola_day15
Bacteroides_plebeius_day15
Barnesiella_intestinihominis_day15
Parabacteroides_distasonis_day15
Prevotella_copri_day15
Alistipes_finegoldii_day15
Alistipes_senegalensis_day15
Lactobacillus_amylovorus_day15
Lactobacillus_amylovorus_day35
Lactobacillus_reuteri_day15
Streptococcus_thermophilus_day15
Clostridiaceae_bacterium_JC118_day56
Pseudoflavonifractor_capillosus_day56
Lachnospiraceae_bacterium_1_1_57FAA_day15
Erysipelotrichaceae_bacterium_21_3_day35
Holdemania_unclassified_day56
Parasutterella_excrementihominis_day15
C2likevirus_unclassified_day35
RMT-CON
RFT-CON
effect size compared with control group
C
pathway abundance differences
amino acids bio.
carbohydrates bio.
nucleotides bio.
photosynthesis
METHGLYUT–PWY_day15
METHGLYUT–PWY_day35
ARGORNPROST–PWY_day15
PWY–5505_day35
PWY–6628_day35
P23–PWY_day15
COLANSYN–PWY_day15
PWY–7323_day15
PWY–2723_day56
PWY–4041_day15
PWY–6588_day56
PWY–6125_day15
PWY–6126_day15
PWY–7220_day15
PWY–7222_day15
PWY0–1479_day15
PWY–241_day15
PWY–7115_day15
PWY–7117_day15
PWY–7446_day56
PWY–7254_day56
PWY–6415_day15
RMT-CON
RFT-CON
effect size compared with control group

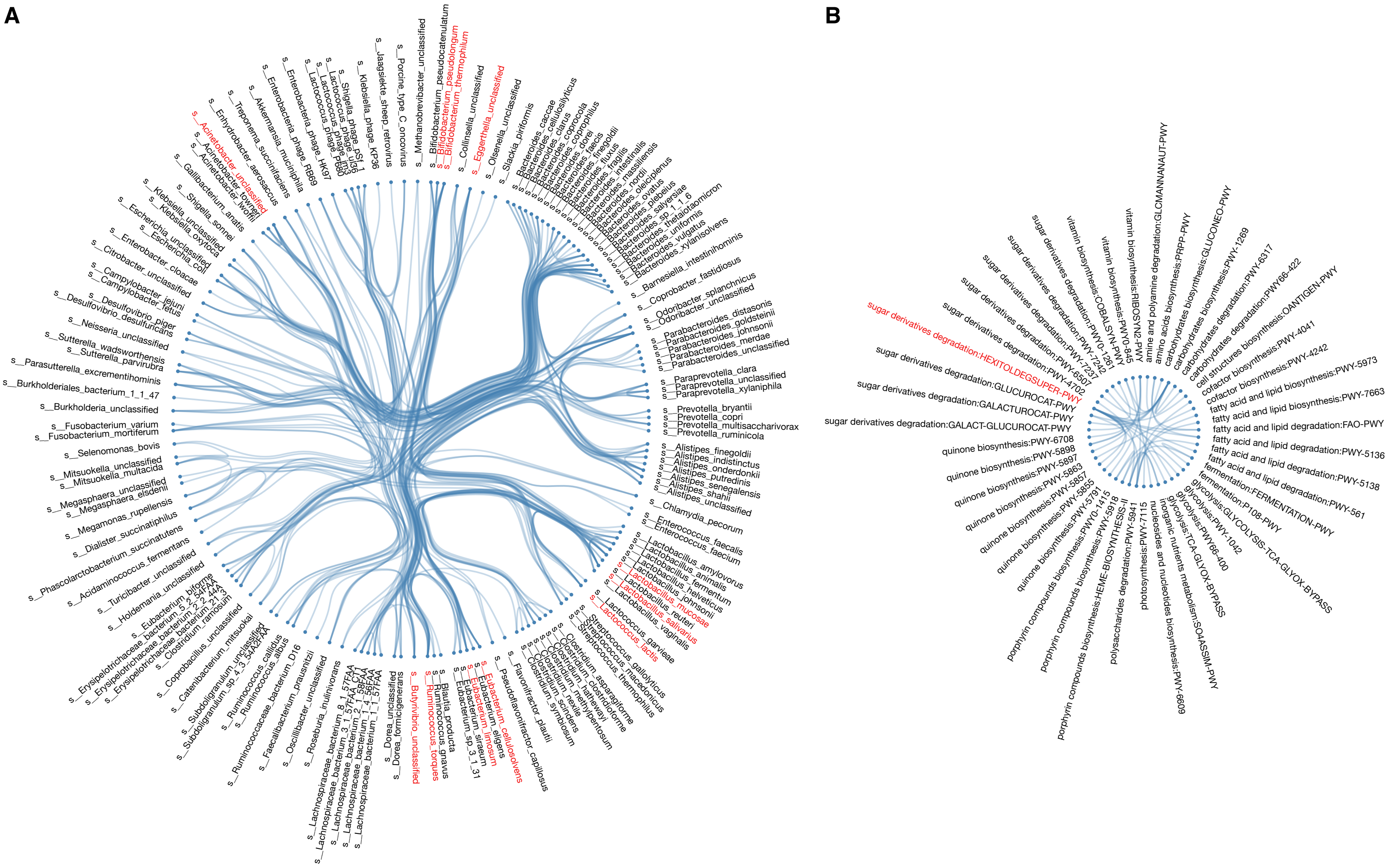
A
B
s__Acinetobacter_unclassified
s__Bifidobacterium_pseudolongum
s__Bifidobacterium_thermophilum
s__Eggerthella_unclassified
s__Collinsella_unclassified
s__Olsenella_unclassified
s__Slackia_piriformis
s__Bifidobacterium_pseudocatenulatum
s__Methanobrevibacter_unclassified
s__Porcine_type_C_oncovirus
s__Jaagsiekte_sheep_retrovirus
s__Klebsiella_phage_KP36
s__Shigella_phage_pSf_1
s__Lactococcus_phage_ul36
s__Lactococcus_phage_P680
s__Enterobacteria_phage_HK97
s__Enterobacteria_phage_RB69
s__Akkermansia_muciniphila
s__Treponema_succinifaciens
s__Enhydrobacter_aerosaccus
s__Acinetobacter_towneri
s__Acinetobacter_lwoffii
s__Gallibacterium_anatis
s__Shigella_sonnei
s__Klebsiella_unclassified
s__Klebsiella_oxytoca
s__Escherichia_unclassified
s__Escherichia_coli
s__Enterobacter_unclassified
s__Enterobacter_cloacae
s__Citrobacter_unclassified
s__Campylobacter_jejuni
s__Campylobacter_fetus
s__Desulfovibrio_piger
s__Desulfovibrio_desulfuricans
s__Neisseria_unclassified
s__Sutterella_wadsworthensis
s__Sutterella_parvirubra
s__Parasutterella_excrementihominis
s__Burkholderiales_bacterium_1_1_47
s__Burkholderia_unclassified
s__Fusobacterium_varium
s__Fusobacterium_mortiferum
s__Selenomonas_bovis
s__Mitsuokella_unclassified
s__Mitsuokella_multacida
s__Megasphaera_unclassified
s__Megasphaera_elsdenii
s__Megamonas_rupellensis
s__Dialister_succinatiphilus
s__Phascolarctobacterium_succinatutens
s__Acidaminococcus_fermentans
s__Turicibacter_unclassified
s__Holdemania_unclassified
s__Eubacterium_biforme
s__Erysipelotrichaceae_bacterium_5_2_54FAA
s__Erysipelotrichaceae_bacterium_2_2_44A
s__Erysipelotrichaceae_bacterium_21_3
s__Clostridium_ramosum
s__Coprobacillus_unclassified
s__Catenibacterium_mitsuokai
s__Subdoligranulum_unclassified
s__Subdoligranulum_sp_4_3_54A2FAA
s__Ruminococcus_callidus
s__Ruminococcus_albus
s__Ruminococcaceae_bacterium_D16
s__Faecalibacterium_prausnitzii
s__Oscillibacter_unclassified
s__Roseburia_inulinivorans
s__Lachnospiraceae_bacterium_8_1_57FAA
s__Lachnospiraceae_bacterium_3_1_57FAA_CT1
s__Lachnospiraceae_bacterium_2_1_58FAA
s__Lachnospiraceae_bacterium_2_1_46FAA
s__Lachnospiraceae_bacterium_1_1_57FAA
s__Dorea_unclassified
s__Dorea_formicigenerans
s__Butyrivibrio_unclassified
s__Ruminococcus_torques
s__Ruminococcus_gnavus
s__Blautia_producta
s__Eubacterium_sp_3_1_31
s__Eubacterium_siraeum
s__Eubacterium_limosum
s__Eubacterium_eligens
s__Eubacterium_cellulosolvens
s__Pseudoflavonifractor_capillosus
s__Flavonifractor_plautii
s__Clostridium_symbiosum
s__Clostridium_spiroforme
s__Clostridium_nexile
s__Clostridium_hathewayi
s__Clostridium_clostridioforme
s__Clostridium_asparagiforme
s__Clostridium_methylpentosum
s__Streptococcus_thermophilus
s__Streptococcus_macedonicus
s__Streptococcus_gallolyticus
s__Lactococcus_lactis
s__Lactococcus_garvieae
s__Lactobacillus_vaginalis
s__Lactobacillus_salivarius
s__Lactobacillus_reuteri
s__Lactobacillus_mucosae
s__Lactobacillus_johnsonii
s__Lactobacillus_helveticus
s__Lactobacillus_fermentum
s__Lactobacillus_animalis
s__Lactobacillus_amylovorus
s__Enterococcus_faecium
s__Enterococcus_faecalis
s__Chlamydia_pecorum
s__Alistipes_unclassified
s__Alistipes_shahii
s__Alistipes_senegalensis
s__Alistipes_putredinis
s__Alistipes_onderdonkii
s__Alistipes_indistinctus
s__Alistipes_finegoldii
s__Prevotella_ruminicola
s__Prevotella_multisaccharivorax
s__Prevotella_copri
s__Prevotella_bryantii
s__Paraprevotella_xylaniphila
s__Paraprevotella_unclassified
s__Paraprevotella_clara
s__Parabacteroides_unclassified
s__Parabacteroides_merdae
s__Parabacteroides_johnsonii
s__Parabacteroides_goldsteinii
s__Parabacteroides_distasonis
s__Odoribacter_unclassified
s__Odoribacter_splanchnicus
s__Coprobacter_fastidiosus
s__Barnesiella_intestinihominis
s__Bacteroides_xylanisolvens
s__Bacteroides_vulgatus
s__Bacteroides_uniformis
s__Bacteroides_thetaiotaomicron
s__Bacteroides_sp_1_1_6
s__Bacteroides_salyersiae
s__Bacteroides_plebeius
s__Bacteroides_ovatus
s__Bacteroides_oleiciplenus
s__Bacteroides_nordii
s__Bacteroides_massiliensis
s__Bacteroides_intestinalis
s__Bacteroides_fragilis
s__Bacteroides_fluxus
s__Bacteroides_finegoldii
s__Bacteroides_faecis
s__Bacteroides_dorei
s__Bacteroides_coprophilus
s__Bacteroides_coprocola
s__Bacteroides_clarus
s__Bacteroides_cellulosilyticus
s__Bacteroides_caccae
sugar derivatives degradation:HEXITOLDEGSUPER-PWY
sugar derivatives degradation:GLUCUROCAT-PWY
sugar derivatives degradation:GALACTUROCAT-PWY
sugar derivatives degradation:GALACT-GLUCUROCAT-PWY
sugar derivatives degradation:PWY-7242
sugar derivatives degradation:PWY-6507
sugar derivatives degradation:PWY-7237
sugar derivatives degradation:PWY-4702
sugar derivatives degradation:PWY0-1261
vitamin biosynthesis:COBALSYN-PWY
vitamin biosynthesis:PWY0-845
vitamin biosynthesis:RIBOSYN2-PWY
amine and polyamine degradation:GLCMANNANAUT-PWY
amino acids biosynthesis:PRPP-PWY
carbohydrates biosynthesis:GLUCONEO-PWY
carbohydrates biosynthesis:PWY-1269
carbohydrates degradation:PWY-6317
carbohydrates degradation:PWY66-422
cell structures biosynthesis:OANTIGEN-PWY
cofactor biosynthesis:PWY-4041
cofactor biosynthesis:PWY-4242
fatty acid and lipid biosynthesis:PWY-5973
fatty acid and lipid biosynthesis:PWY-7663
fatty acid and lipid degradation:FAO-PWY
fatty acid and lipid degradation:PWY-5136
fatty acid and lipid degradation:PWY-5138
fatty acid and lipid degradation:PWY-561
fermentation:FERMENTATION-PWY
fermentation:P108-PWY
glycolysis:GLYCOLYSIS-TCA-GLYOX-BYPASS
glycolysis:PWY-1042
glycolysis:PWY66-400
glycolysis:TCA-GLYOX-BYPASS
inorganic nutrients metabolism:SO4ASSIM-PWY
nucleosides and nucleotides biosynthesis:PWY-6609
photosynthesis:PWY-7115
polysaccharides degradation:PWY-5941
porphyrin compounds biosynthesis:HEME-BIOSYNTHESIS-II
porphyrin compounds biosynthesis:PWY-5918
porphyrin compounds biosynthesis:PWY0-1415
quinone biosynthesis:PWY-5791
quinone biosynthesis:PWY-5855
quinone biosynthesis:PWY-5857
quinone biosynthesis:PWY-5863
quinone biosynthesis:PWY-5897
quinone biosynthesis:PWY-5898
quinone biosynthesis:PWY-6708

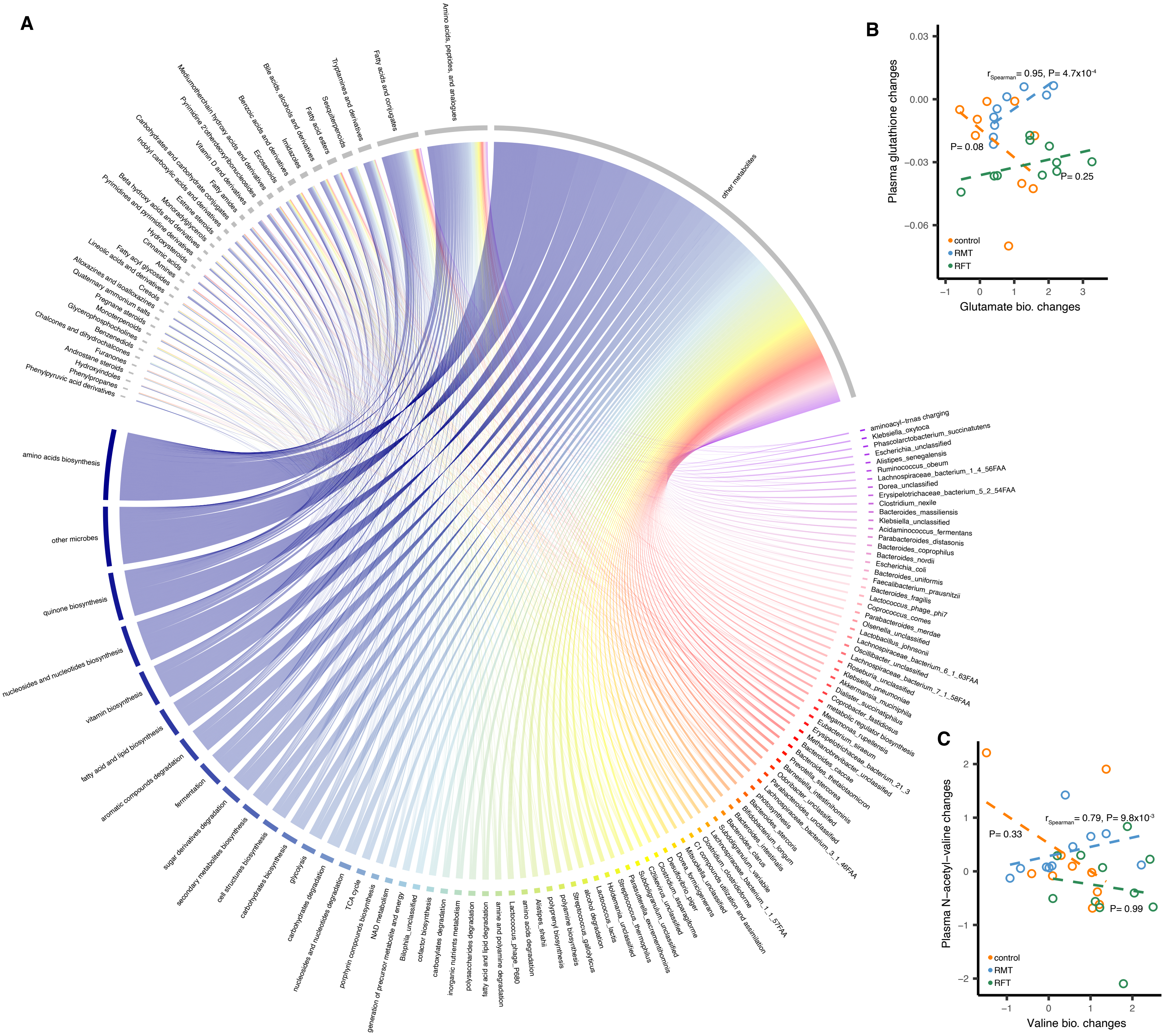
A
Amino acids, peptides, and analogues
Fatty acids and conjugates
Tryptamines and derivatives
Sesquiterpenoids
Fatty acid esters
Bile acids, alcohols and derivatives
Imidazoles
Benzoic acids and derivatives
Eicosanoids
Mediumotherchain hydroxy acids and derivatives
Pyrimidine 2'otherdeoxyribonucleosides
Vitamin D and derivatives
Fatty amides
Carbohydrates and carbohydrate conjugates
Indolyl carboxylic acids and derivatives
Estrane steroids
Monoradylglycerols
Beta hydroxy acids and derivatives
Pyrimidines and pyrimidine derivatives
Hydroxysteroids
Cinnamic acids
Amines
Fatty acyl glycosides
Lineolic acids and derivatives
Cresols
Alloxazines and isoalloxazines
Quaternary ammonium salts
Pregnane steroids
Monoterpenoids
Glycerophosphocholines
Benzenediols
Chalcones and dihydrochalcones
Furanones
Androstane steroids
Hydroxyindoles
Phenylpropanes
Phenylpyruvic acid derivatives
other metabolites
amino acids biosynthesis
other microbes
quinone biosynthesis
nucleosides and nucleotides biosynthesis
vitamin biosynthesis
fatty acid and lipid biosynthesis
aromatic compounds degradation
fermentation
sugar derivatives degradation
secondary metabolites biosynthesis
cell structures biosynthesis
carbohydrates biosynthesis
glycolysis
carbohydrates degradation
nucleosides and nucleotides degradation
TCA cycle
porphyrin compounds biosynthesis
NAD metabolism
generation of precursor metabolite and energy
Bilophila_unclassified
cofactor biosynthesis
carboxylates degradation
inorganic nutrients metabolism
polysaccharides degradation
fatty acid and lipid degradation
amine and polyamine degradation
Lactococcus_phage_P680
amino acids degradation
Alistipes_shahii
polyprenyl biosynthesis
polyamine biosynthesis
Streptococcus_gallolyticus
alcohol degradation
Lactococcus_lactis
Holdemania_unclassified
Streptococcus_thermophilus
Parasutterella_excrementihominis
C2Hkvirus_unclassified
Subdoligranulum_unclassified
Clostridium_asparagiforme
Desulfovibrio_piger
Dorea_formicigenerans
Mitsuokella_unclassified
C1 compounds utilization and assimilation
Clostridium_clostridioforme
Lachnospiraceae_bacterium_1_1_57FAA
Subdoligranulum_variabile
Bacteroides_clarus
Bifidobacterium_longum
Bacteroides_intestinalis
Bacteroides_stercoris
photosynthesis
Lachnospiraceae_bacterium_3_1_46FAA
Odoribacter_unclassified
Parabacteroides_unclassified
Barnesiella_intestinihominis
Prevotella_stercorea
Bacteroides_thetaiotaomicron
Bacteroides_caccae
Methanobrevibacter_unclassified
Erysipelotrichaceae_bacterium_21_3
Eubacterium_siraeum
Megamonas_rupellensis
metabolic regulator biosynthesis
Coprobacter_fastidiosus
Dialister_succinatiphilus
Akkermansia_muciniphila
Klebsiella_pneumoniae
Roseburia_unclassified
Lachnospiraceae_bacterium_7_1_58FAA
Oscillibacter_unclassified
Lachnospiraceae_bacterium_6_1_63FAA
Lactobacillus_johnsonii
Olsenella_unclassified
Parabacteroides_merdae
Coprococcus_comes
Lactococcus_phage_phi7
Bacteroides_fragilis
Faecalibacterium_prausnitzii
Bacteroides_uniformis
Escherichia_coli
Bacteroides_nordii
Bacteroides_coprophilus
Parabacteroides_distasonis
Acidaminococcus_fermentans
Klebsiella_unclassified
Bacteroides_massiliensis
Clostridium_nexile
Erysipelotrichaceae_bacterium_5_2_54FAA
Dorea_unclassified
Lachnospiraceae_bacterium_1_4_56FAA
Ruminococcus_obeum
Alistipes_senegalensis
Escherichia_unclassified
Phascolarctobacterium_succinatutens
Klebsiella_oxytoca
aminoacyl-trnas charging
B
Plasma glutathione changes
Glutamate bio. changes
r_Spearman = 0.95, P= 4.7x10^-4
P= 0.08
P= 0.25
0.03
0.00
−0.03
−0.06
−1
0
1
2
3
control
RMT
RFT
C
Plasma N−acetyl−valine changes
Valine bio. changes
r_Spearman = 0.79, P= 9.8x10^-3
P= 0.33
P= 0.99
2
1
0
−1
−2
−1
0
1
2
control
RMT
RFT

Figure6

Click here to access/download;Figure;Figure6.pdf

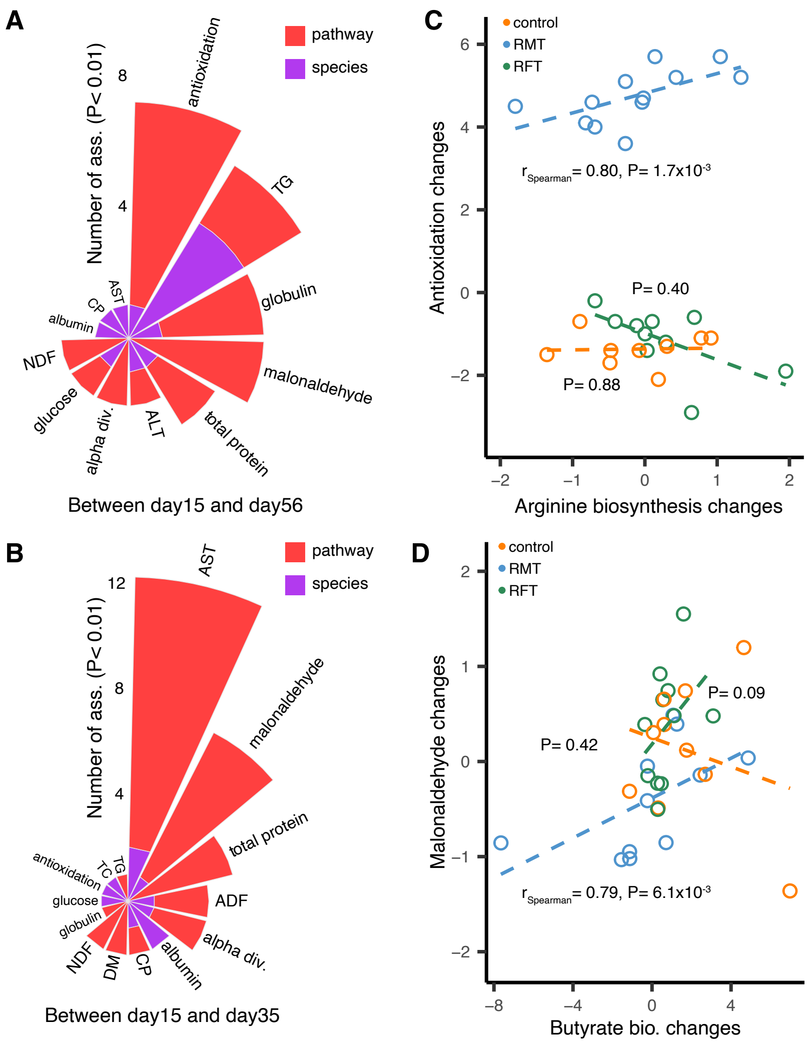

Click here to access/download;Figure;Figure7.pdf

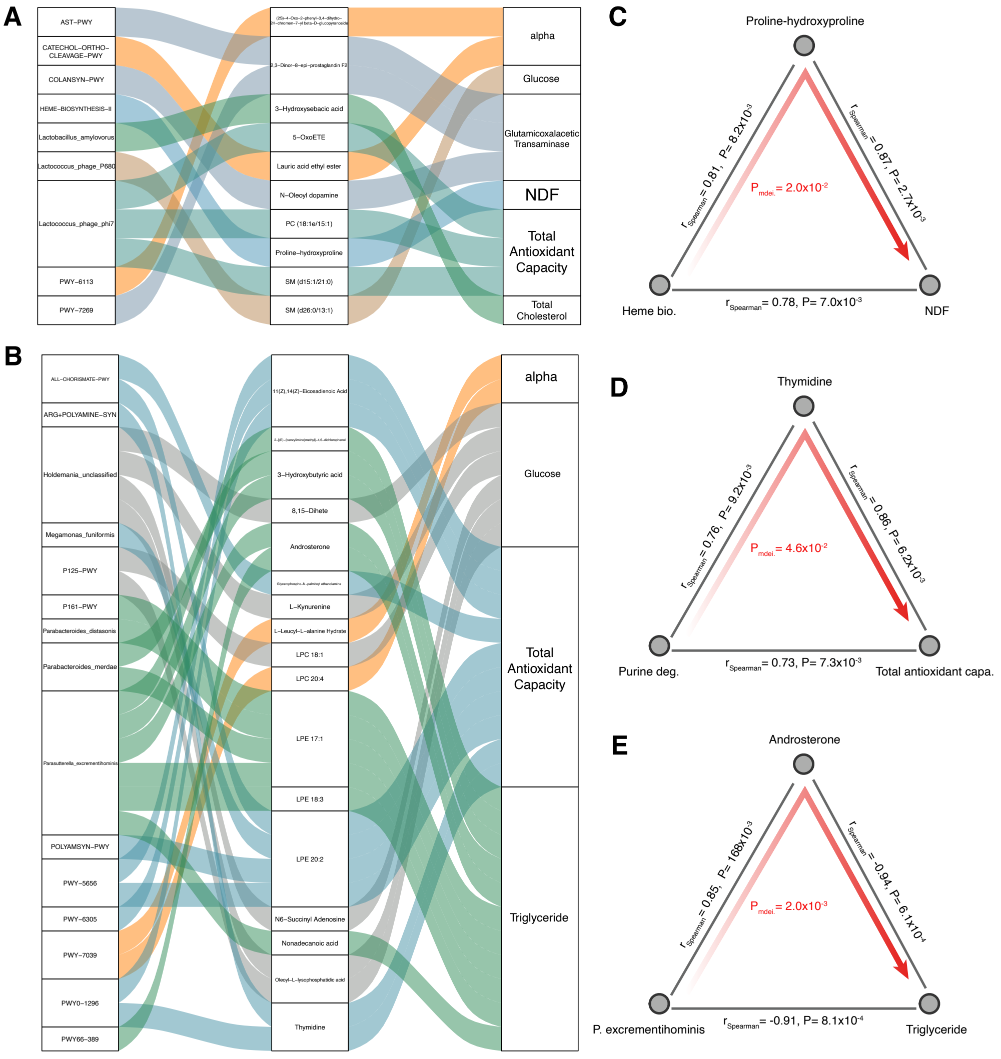

FigureS1

Click here to access/download

Supplementary Material

FigureS1.pdf

FigureS2

Click here to access/download
Supplementary Material
FigureS2.pdf

FigureS3

Click here to access/download
Supplementary Material
FigureS3.pdf

FigureS4

Click here to access/download
Supplementary Material
FigureS4.pdf

FigureS5

Click here to access/download
Supplementary Material
FigureS5.pdf

Click here to access/download
Supplementary Material
SuppTables.xlsx
