# Supplementary material for: Early microbial intervention reshapes phenotypes of newborn Bos taurus through metabolic regulations
Source: Gigascience. 2024 Jan 13;13:giad118. doi: 10.1093/gigascience/giad118 (PMC10787367; doi:10.1093/gigascience/giad118)
Supplement: giad118_Response_to_Reviewer_Comments_Original_Submission [file giad118_response_to_reviewer_comments_original_submission.pdf]

Author's Response To Reviewer Comments

detailed responses have been uploaded.
