# Supplementary material for: Early microbial intervention reshapes phenotypes of newborn Bos taurus through metabolic regulations
Source: Gigascience. 2024 Jan 13;13:giad118. doi: 10.1093/gigascience/giad118 (PMC10787367; doi:10.1093/gigascience/giad118)
Supplement: giad118_Reviewer_1_Report_Original_Submission — Huanzi Zhong, Ph.D. -- 5/29/2023 Reviewed [file giad118_reviewer_1_report_original_submission.pdf]

Reviewer Report

Title: Early microbial intervention reshapes phenotypes of newborn Bos taurus through metabolic regulations

Version: Original Submission **Date:** 5/29/2023

Reviewer name: Huanzi Zhong, Ph.D.

Reviewer Comments to Author:

In this manuscript, the authors applied shotgun metagenomics and metabolomics to investigate the temporal development of gut microbiota (GM) in neonatal calves and the effects of rumen microbiota transplantation (RMT) on the calves' GM, metabolism, and later milk production performance. The manuscript is well-written. The study is carefully designed, with an extensive collection of omics data from fecal samples, blood samples, ruminal fluid, and the calves themselves. Comprehensive analyses linking host characteristics, fecal microbial features, and blood metabolites are conducted. The descriptions of the metagenomic methods and statistical approaches are clear and understandable. I have the following suggestions to improve the manuscript.

Methods:

1ï¼‰ Line 102-Line 118. Two different transplantation methods (RMT and RFT) were applied in this study. The RMT group was treated with fresh ruminal fluid and the RFT group was treated with autoclaved ruminal fluid. Additionally, the intervention volume and feeding period varied a lot during the whole experiment, (e.g., a volume of 50 mL, 80 mL, and 110 mL of 116 ruminal fluid was fed from day 7 to day 11, day 21 to day 25, and day 42 to day 46, respectively).

I suggest the author should revise Figure S1 to include more details, specifically highlighting the differences between the two transplantation methods, the intervention periods, and the volume of transplantation. This addition, together with the sampling and measurements, would enhance the clarity of the experimental pipeline for readers.

2ï¼‰ Line 112-Line 128. Most previous metagenomic studies have investigated the rumen microbiota and revealed a highly complex microbial community within this specific environment. For this study, the fecal samples of calves yielded an average of 36.8 million (sd= 3.6 million) paired reads per sample. It is important to note that this sequencing depth is significantly lower than what is typically required for rumen samples in ruminant studies. which is much lower than the that required by the rumen samples of ruminants. Therefore, it is unclear whether the authors evaluated the sequencing depth saturation specifically for the fecal samples and whether they consider the obtained depth to be sufficient for their research.

Result:

First, one of the major concerns of the design is the lack of baseline samples before intervention. As shown in the Fig. S1, the first sampling point of the feces was day 15 while the first intervention period was from day 7- day 11. None of baseline microbial data could be acquired to show whether the three groups have overall similar or different fecal microbial community. The second concern could be the lack of rumen microbiota of the fresh ruminal fluid from the healthy donor. Without the data, we are not

able to answer how the specific donor affected the receptors' gut microbiota, thus we could hardly genialize the observations/conclusions to larger animal populations. The authors should discuss both as the possible limitations in the manuscript.
1ï¼‰ Line 257-273. The authors should add some descriptions to show that there were no baseline differences in host characterizes measured at day 1. Line267-269, the results showed that the plasma total antioxidant capacity increased from day 15 to day 35 while dramatically decreased from day 35 to day 56, even reached a level lower than day 15. Why? Could the authors provide any explanations on this temporal changes? Comparisons between different timepoints (day 15, 35 and 56) within each group would be helpful to improve the conclusions for this part (Early microbial interventions using RMT could provide beneficial effects on the health and growth of newborn calves, as well as their later milk production performance).
PS, in FigS6, they showed few baseline differences in plasma metabolites between the three groups. I would suggest authors to put some baseline results in the first section.
2ï¼‰ Line 274-312. The authors dedicated a lot of effort to elucidate the temporal variations in the gut microbial composition of calves from day 15 to day 56. Overall, their findings are reliable. Across all three intervention groups, they observed significant differences in the gut microbiota between day 15 and either day 35 or day 56, while the gut microbial structure between day 35 and day 56 showed no significant differences (or much smaller differences). However, all of these results are presented in supplementary figures or tables, which makes it inconvenient for readers to access comprehensive information. I would suggest that the authors consolidate the relevant results from the supplementary figures/tables as a main figure (e.gï¼ŒFig S2, Fig S3-specise-level based results, Fig S4B and related Stable3-5) to clearly describe the directions of specific temporal changes, e.g, the RA changes of Bacteroides members and the amino acid and nucleotide biosynthesis pathways (increase or decrease), instead of give the number of microbial features differed between timepoints, and the changes of networks for Bacteroides members.
3ï¼‰ Line 290-292. Data from Fig S3 and Fig S4 both suggested that unlike species community, the microbial functional pathways between three timepoints were highly similar (rho>0.9 and ~3% heterogeneity). SO, I suggest that the authors modify their results "Notably, when comparing the mean abundances of microbial species and pathways between time points, we observed that microbial abundances at Day 35 and Day 56 were more similar than those at Day 15 (Figure S3). " Because in Fig SC, the association results at the pathway level did not support the text "pathway abundances at Day 35 and Day 56 were more similar than those at Day 15". You should cite it together with Fig S4A (Line 299-304) to indicate that the overall pathway RA matrix and pathway interactions were relatively stable over time.
4ï¼‰ Line 313-331. Fig.2. All conclusions were based on between-group comparisons. However, from this figure, we could hardly get the conclusion that "differential microbial abundances between groups were mainly observed at Day 15, and most of them were driven by RMT". If we look at the data, we did see that in the RMT group, the species BC distance increased from day 15 to day 35 and then decreased from day 35 to day 56. But that patterns seems did not exist in other groups. Could the authors please provide between-timepoint comparisons (BC distance, species changes in the RMT group) within each intervention group together in this figure (instead of putting it in the Stables) to complete their findings? Also, a Dunn's post-hoc test would be needed after the KW test (Fig. 4A-C).

For the Fig. 4B-C, the authors utilize the transformed RA as the color key for the heatmap, making it difficult for readers to discern the differences (enrichment directions) among the three intervention groups. I would suggest that they consider using effect size (compared to the control group) as a more suitable measure for the figure. This approach would enhance the clarity of the heatmap and facilitate a clearer understanding of the differences between the control and intervention groups.
5ï¼‰ Line 332-359. The authors described the differences in species or pathway co-abundance networks between groups, primarily focusing on the number of unique connections within the RMT group. Could they conduct further analysis to identify potential hub nodes (hub species/pathways) under different conditions? Additionally, highlighting the hub nodes in the figures would greatly enhance the clarity and aid readers in understanding the results more effectively. This additional information would provide valuable insights into the central species or pathways that play critical roles in the observed differences.
6ï¼‰ Line 360-399. The main issue is the interpretation of association results. As the authors also discussed in their manuscript, they might overinterpret their association results: "we observed that an increased abundance of Megamonas funiformis may potentially promote the antioxidation capacity of newborn calves" "An increased abundance of M. funiformis may contribute to arginine biosynthesis (rSpearman= 0.65, P=Figure 4E), indicating that RMT may increase M. funiformis abundance to potentially promote arginine production, which further enhances the antioxidation capacity." We further…., indicating that increased abundance of R. gnavus may potentially promote butyrate production in response to the increased amount of malonaldehyde in the RMT group. These results suggest that RMT may potentially promote phenotypes of newborn calves by modulating microbial functionalities."
From a logical perspective, the functions attributed to a particular species or taxon should be supported by evidence from potential coding genes and pathways within their genomes (considered as first-level evidence). Therefore, it is recommended that the authors avoid solely attributing changes in metabolites to their associations with the gut microbiota, specifically relying on correlations between alterations in the relative abundances of microbial species and blood metabolites.
Furthermore, it is worth noting that the increased RAs of M. funiformis and R. gnavus have previously been reported to be enriched in humans with obesity or inflammatory bowel disease (IBD). Considering this, it raises questions as to why the authors concluded that these microbial changes might be beneficial in the RMT group. Although MP2/MP3 has the potential to annotate the microbial community in human samples well, it remains uncertain to what extent it can accurately represent the complexity of the gastrointestinal microbial community in ruminants. This is also why most current published studies prefer to employ an assembly-based strategy to investigate this type of microbial community.
It is advisable for the authors to exercise caution and refrain the results for this particular section. (e.g., the changes in several specific blood metabolites should reflect more directly to a beneficial outcome of the RMT group. Think you could spend more words on these points.)
7ï¼‰
Line 360-399. Microbial changes associated with the phenotypes of newborn calves
Line 400-421. Microbial changes associated with plasma metabolites in newborn calves
I would suggest combining the two sections and adjusting their order as follows: First, for the relationship between changes in gut microbiota and metabolites. Then, explore the relationships

between changes in gut microbiota and phenotypes. Currently, in the section titled "Microbial changes associated with the phenotypes of newborn calves," the authors have already integrated some information regarding the changes in metabolites and gut microbiota. By reorganizing the sections in this manner, the flow of information will be improved, providing a clearer understanding of the relationships between three parties.
8ï¼‰ Line 422-439. The main concern for this section is the relatively small samples size for mediation analysis. It is crucial to conduct a power analysis to determine the minimum sample size required for mediation analysis.
9ï¼‰ Discussion: Please revise it according to the above suggestions.

Methods

Are the methods appropriate to the aims of the study, are they well described, and are necessary controls included? Choose an item.

Conclusions

Are the conclusions adequately supported by the data shown? Choose an item.

Reporting Standards

Does the manuscript adhere to the journal's guidelines on minimum standards of reporting? Choose an item.

Choose an item.

Statistics

Are you able to assess all statistics in the manuscript, including the appropriateness of statistical tests used? Choose an item.

Quality of Written English

Please indicate the quality of language in the manuscript: Choose an item.

Declaration of Competing Interests

Please complete a declaration of competing interests, considering the following questions:

- Have you in the past five years received reimbursements, fees, funding, or salary from an organisation that may in any way gain or lose financially from the publication of this manuscript, either now or in the future?
- Do you hold any stocks or shares in an organisation that may in any way gain or lose financially from the publication of this manuscript, either now or in the future?

- Do you hold or are you currently applying for any patents relating to the content of the manuscript?
- Have you received reimbursements, fees, funding, or salary from an organization that holds or has applied for patents relating to the content of the manuscript?
- Do you have any other financial competing interests?
- Do you have any non-financial competing interests in relation to this paper?

If you can answer no to all of the above, write 'I declare that I have no competing interests' below. If your reply is yes to any, please give details below.

I declare that I have no competing interests.

I agree to the open peer review policy of the journal. I understand that my name will be included on my report to the authors and, if the manuscript is accepted for publication, my named report including any attachments I upload will be posted on the website along with the authors' responses. I agree for my report to be made available under an Open Access Creative Commons CC-BY license (http://creativecommons.org/licenses/by/4.0/). I understand that any comments which I do not wish to be included in my named report can be included as confidential comments to the editors, which will not be published.

Choose an item.

To further support our reviewers, we have joined with Publons, where you can gain additional credit to further highlight your hard work (see: https://publons.com/journal/530/gigascience). On publication of this paper, your review will be automatically added to Publons, you can then choose whether or not to claim your Publons credit. I understand this statement.

Yes Choose an item.
