# Supplementary material for: Early microbial intervention reshapes phenotypes of newborn Bos taurus through metabolic regulations
Source: Gigascience. 2024 Jan 13;13:giad118. doi: 10.1093/gigascience/giad118 (PMC10787367; doi:10.1093/gigascience/giad118)
Supplement: giad118_Reviewer_1_Report_Revision_1 — Huanzi Zhong, Ph.D. -- 9/14/2023 Reviewed [file giad118_reviewer_1_report_revision_1.pdf]

Reviewer Report

Title: Early microbial intervention reshapes phenotypes of newborn Bos taurus through metabolic regulations

Version: Revision 1 **Date:** 9/14/2023

Reviewer name: Huanzi Zhong, Ph.D.

Reviewer Comments to Author:

The authors have made substantial improvements in addressing most of my previous comments in the revised manuscript. However, there are still a few minor typos and questions that need to be addressed.
1) Figure 2 (line 547-553)
The data presented on 2C and 2D were at the genus level but not the species level.
Please revise the legends of panel C and D, and also legends for all main/sup figures.
2) Figure 3C
Please modify the color key as it does not represent the transformed abundances but the effect size).
Please also add the full name of each pathway as readers could hardly get useful information from the pathway IDs. e.g., PWY-6588 -pyruvate fermentation to acetone.
Line 68-70. The authors mentioned that "In addition, comparison of the gut microbial composition between calves (8 weeks after birth) and lactating cows showed that Bacteroidetes and Verrumicrobia were more abundant in calves, while Firmicutes, Spirochaetes, Deinococcusthermus, Lentisphaerae, Planctomycetes, and Chlorofexi were more abundant in cows [9]."
There is a lack of their own data to generally describe the gut microbial composition of calves at different time points (birth~8 weeks). For instance, the top 5 phyla, as well as the top 10/20 abundant genera at different time points across different groups. Any there any differences /similarity between their results and previous results sampling after 8 weeks?
Other concerns:
"In this study, the authors examined the gut microbial differences between RMT, RFT and controls of newborn calves and reported that their gut microbiomes likely reached maturity one month after birth". However, all of the samples collected in this study are pre-weaning calves. The authors also stated in the discussion that "The period from birth to weaning is important for rumen microbial colonization and adaptation". Likewise, breastfeed duration has shown strong impacts in shaping the gut microbiota of human infants. Thus, it is still unknown whether the gut microbiota of calves will further develop after weaning (possibly Yes).
"In this study, the authors examined the gut microbial differences between RMT, RFT, and control groups of newborn calves. They reported that the gut microbiomes of these calves likely reached maturity one month after birth. However, it's important to note that all samples collected in this study are from pre-weaning calves. In the discussion, the authors pointed the significance of the period from birth to weaning in terms of rumen microbial colonization and adaptation. Similarly, the duration of breastfeeding has been shown to strongly impacts on the gut microbiota of human infants, particularly on the Bifidobacterium members. However, it remains uncertain whether the gut microbiota of calves

will continue to evolve after weaning. This question warrants further investigation. The authors should carefully to state with "maturity".

Methods

Are the methods appropriate to the aims of the study, are they well described, and are necessary controls included? Choose an item.

Conclusions

Are the conclusions adequately supported by the data shown? Choose an item.

Reporting Standards

Does the manuscript adhere to the journal's guidelines on minimum standards of reporting? Choose an item.

Choose an item.

Statistics

Are you able to assess all statistics in the manuscript, including the appropriateness of statistical tests used? Choose an item.

Quality of Written English

Please indicate the quality of language in the manuscript: Choose an item.

Declaration of Competing Interests

Please complete a declaration of competing interests, considering the following questions:

- Have you in the past five years received reimbursements, fees, funding, or salary from an organisation that may in any way gain or lose financially from the publication of this manuscript, either now or in the future?
- Do you hold any stocks or shares in an organisation that may in any way gain or lose financially from the publication of this manuscript, either now or in the future?
- Do you hold or are you currently applying for any patents relating to the content of the manuscript?
- Have you received reimbursements, fees, funding, or salary from an organization that holds or has applied for patents relating to the content of the manuscript?
- Do you have any other financial competing interests?
- Do you have any non-financial competing interests in relation to this paper?

If you can answer no to all of the above, write 'I declare that I have no competing interests' below. If your reply is yes to any, please give details below.

'I declare that I have no competing interests.

I agree to the open peer review policy of the journal. I understand that my name will be included on my report to the authors and, if the manuscript is accepted for publication, my named report including any attachments I upload will be posted on the website along with the authors' responses. I agree for my report to be made available under an Open Access Creative Commons CC-BY license (http://creativecommons.org/licenses/by/4.0/). I understand that any comments which I do not wish to be included in my named report can be included as confidential comments to the editors, which will not be published.

Choose an item.

To further support our reviewers, we have joined with Publons, where you can gain additional credit to further highlight your hard work (see: https://publons.com/journal/530/gigascience). On publication of this paper, your review will be automatically added to Publons, you can then choose whether or not to claim your Publons credit. I understand this statement.

Yes Choose an item.
