# Supplementary material for: Early microbial intervention reshapes phenotypes of newborn Bos taurus through metabolic regulations
Source: Gigascience. 2024 Jan 13;13:giad118. doi: 10.1093/gigascience/giad118 (PMC10787367; doi:10.1093/gigascience/giad118)
Supplement: giad118_Reviewer_2_Report_Original_Submission — Zhipeng Li -- 6/1/2023 Reviewed [file giad118_reviewer_2_report_original_submission.pdf]

Reviewer Report

Title: Early microbial intervention reshapes phenotypes of newborn Bos taurus through metabolic regulations

Version: Original Submission **Date:** 6/1/2023

Reviewer name: Zhipeng Li

Reviewer Comments to Author:

In this study, Shen et al. investigated the gut microbiota colonization, and evaluated the effect of rumen microbiota and metabolites transplantation. Although many studies have examined the gut microbiota development of different ruminant, however, the RMT is interesting. The present version limited to the large part of description in difference or association. More analysis is need to answer why RMT can reshape the gut microbiota and host metabolism based on metagenome and metabolome.
L59. What is modification of nutrition? Please explain.
L77. However, investigations of the temporal dynamics of microbial functionalities of newborn calves are still lacking. Malmuthuge et al. (2019) has examined the development of rumen microbiome through metagenomes.
L85. it is not clear at what time the gut microbiome reaches maturity in calves. The authors conducted the rumen microbiota transplantation, but examined the gut microbiota maturity. Make no sense. Moreover, the gut microbiota development has reported in jejunum, cecum, and colon, such as Zhuang et al. (2020) and Fan et al. (2021).
L115. Why the author choose 50 mL, 80 mL, and 110 mL?
L138. Please define customized database.
L245. Since we observed that microbial differences between Day 35 and Day 56 were minor. This is a result before conducing statistics analysis. So rewrite the sentence.
L251. Why the author examined the human phenotype?
L277. Change x.
L320. Although the inter-calf dissimilarities within the RMT group were always the lowest among those of all three groups at different time points, however, the t-SNE results showed that the gut microbiota in the two treatment groups is not significant different, and only 4 species and pathways showed a significant difference. These limited results can not provide the solid support to reshape the gut microbiome composition.
L326-328. Differential microbial species were enriched in the Bacteroidetes and Firmicutes families, while differential pathways were mostly enriched in nucleotide, amino acid and carbohydrate biosynthesis pathways. Bacteroidetes and Firmicutes were families? Most of microbial species can be classified as the phyla Bacteroidetes and Firmicutes. Moreover, the nucleotide, amino acid and carbohydrate biosynthesis pathways were too general.
L342. many of the species and pathway co-abundances showed heterogeneity between groups at different time points. This may be Ture, as stochastic colonization in early life has long-lasting impacts on the development of animal microbiomes (Furman, 2020).

L366. 79 species, 185 pathways were identified in the associations. Is there any difference for these species and pathways among the groups? Is there any trend or distribution in these species and pathways? More analysis is need. The present results contributed to limited novel insights for understanding the relationship between gut microbiota and host phenotype.
L405. The results showed that the metabolites at day 35 was different from that at day 56. However, this result is different from the observed results in gut microbiota. Why?
The font size in figures 3, 5 and 6 is too small.

Methods

Are the methods appropriate to the aims of the study, are they well described, and are necessary controls included? Choose an item.

Conclusions

Are the conclusions adequately supported by the data shown? Choose an item.

Reporting Standards

Does the manuscript adhere to the journal's guidelines on [minimum standards of reporting?] Choose an item.

Choose an item.

Statistics

Are you able to assess all statistics in the manuscript, including the appropriateness of statistical tests used? Choose an item.

Quality of Written English

Please indicate the quality of language in the manuscript: Choose an item.

Declaration of Competing Interests

Please complete a declaration of competing interests, considering the following questions:

- Have you in the past five years received reimbursements, fees, funding, or salary from an organisation that may in any way gain or lose financially from the publication of this manuscript, either now or in the future?
- Do you hold any stocks or shares in an organisation that may in any way gain or lose financially from the publication of this manuscript, either now or in the future?
- Do you hold or are you currently applying for any patents relating to the content of the manuscript?

- Have you received reimbursements, fees, funding, or salary from an organization that holds or has applied for patents relating to the content of the manuscript?
- Do you have any other financial competing interests?
- Do you have any non-financial competing interests in relation to this paper?

If you can answer no to all of the above, write 'I declare that I have no competing interests' below. If your reply is yes to any, please give details below.

I declare that I have no competing interests

I agree to the open peer review policy of the journal. I understand that my name will be included on my report to the authors and, if the manuscript is accepted for publication, my named report including any attachments I upload will be posted on the website along with the authors' responses. I agree for my report to be made available under an Open Access Creative Commons CC-BY license (http://creativecommons.org/licenses/by/4.0/). I understand that any comments which I do not wish to be included in my named report can be included as confidential comments to the editors, which will not be published.

Choose an item.

To further support our reviewers, we have joined with Publons, where you can gain additional credit to further highlight your hard work (see: https://publons.com/journal/530/gigascience). On publication of this paper, your review will be automatically added to Publons, you can then choose whether or not to claim your Publons credit. I understand this statement.

Yes Choose an item.
